# Development and Interrogation of a Transcriptomic Resource for the Giant Triton Snail (*Charonia tritonis*)

**DOI:** 10.1007/s10126-021-10042-7

**Published:** 2021-06-30

**Authors:** AH Klein, CA Motti, AK Hillberg, T Ventura, P Thomas-Hall, T Armstrong, T Barker, P Whatmore, SF Cummins

**Affiliations:** 1 Genecology Research Centre, University of the Sunshine Coast, Maroochydore DC, QLD 4558 Australia; 2 School of Science, Technology and Engineering, University of the Sunshine Coast, Maroochydore DC, QLD 4558 Australia; 3 Australian Institute of Marine Science (AIMS), Cape Ferguson, Townsville, QLD 4810 Australia; 4 eResearch Office, Queensland University of Technology, Brisbane, QLD 4000 Australia

**Keywords:** Olfaction, Cytochrome P450, G protein-coupled receptor, Neuropeptide, Crown-of-thorns starfish

## Abstract

Gastropod molluscs are among the most abundant species that inhabit coral reef ecosystems. Many are specialist predators, along with the giant triton snail *Charonia tritonis* (Linnaeus, 1758) whose diet consists of *Acanthaster planci* (crown-of-thorns starfish), a corallivore known to consume enormous quantities of reef-building coral. *C. tritonis* are considered vulnerable due to overexploitation, and a decline in their populations is believed to have contributed to recurring *A. planci* population outbreaks. Aquaculture is considered one approach that could help restore natural populations of *C. tritonis* and mitigate coral loss; however, numerous questions remain unanswered regarding their life cycle, including the molecular factors that regulate their reproduction and development. In this study, we have established a reference *C. tritonis* transcriptome derived from developmental stages (embryo and veliger) and adult tissues. This was used to identify genes associated with cell signalling, such as neuropeptides and G protein-coupled receptors (GPCRs), involved in endocrine and olfactory signalling. A comparison of developmental stages showed that several neuropeptide precursors are exclusively expressed in post-hatch veligers and functional analysis found that FFamide stimulated a significant (20.3%) increase in larval heart rate. GPCRs unique to veligers, and a diversity of rhodopsin-like GPCRs located within adult cephalic tentacles, all represent candidate olfactory receptors. In addition, the cytochrome P450 superfamily, which participates in the biosynthesis and degradation of steroid hormones and lipids, was also found to be expanded with at least 91 genes annotated, mostly in gill tissue. These findings further progress our understanding of *C. tritonis* with possible application in developing aquaculture methods.

**Supplementary Information:**

The online version contains supplementary material available at 10.1007/s10126-021-10042-7.

## Introduction

The giant triton snail, *Charonia tritonis* (Family Ranellidae), is one of the largest gastropod snails, protected by a long, narrow shell that can measure up to half a meter in length (Hall et al. 2017a). They are found throughout the Indo-West Pacific region, primarily in tropical waters where the habitat consists of hard and sandy bottoms adjacent to shallow water reefs (Nateewathana and Aungtonya 1994). As one of the few active predators of adult crown-of-thorns starfish (*Acanthaster planci*; COTS), it is of significant ecological importance on coral reefs. Although an adult *C. tritonis* eats only 1.5 COTS per week on average (Endean 1969), its mere presence alters the behaviour of COTS (Hall et al. 2017a, b). This chemically mediated phenomenon has been referred to as “landscapes of fear” (Luttbeg and Kerby 2005; Preisser et al. 2005; Preisser and Bolnick 2008), whereby predator presence can induce changes in prey at the physiological, phenotypic and behavioural levels (Brown and Alexander 1994; Abrams 1995; Schmitz et al. 1997; Pinnegar et al. 2000; Bernot and Turner 2001; Dill et al. 2003; Witman et al. 2003; Bolnick and Preisser 2005; Toscano and Griffen 2014; Hall and Kingsford 2016; Morgan et al. 2016) and can strongly influence prey density (Luttbeg and Kerby 2005; Peckarsky et al. 2008a, b; Paterson et al. 2013). It has been postulated that the “zone of fear” produced by *C. tritonis* has the potential to prevent COTS aggregation, ultimately leading to decreased offspring due to altered reproductive capacity (Hall et al. 2017b).

As a result of overexploitation, *C. tritonis* is now considered rare across its tropical habitat. Unfortunately, a general lack of fundamental understanding of their biology has impacted our capacity to implement conservation through captive breeding and mitigate further damage caused by increasingly frequent COTS outbreaks. Basic information is known regarding *C. tritonis* mating behaviour and early life-stage development (embryonic/larval). For example, during mating, males mount the female shell, aligning their apertures to enable copulation, which can last up to 2 h (Zhang et al. 2013). It is thought that change in water temperature is a primary determinate for initiation of egg-laying (Thorson 1950), where a single female *C. tritonis* can lay between 2 to 50 egg capsules per day (over a 3-month period), each capsule containing 2000 to 2750 fertilized eggs (Hall 2017, personal communication). Embryogenesis into the first veliger stage occurs within the egg capsule (up to 60 days post-fertilization), enhancing protection from predation. Post-hatch, planktonic larvae have been maintained for over 140 days (Nugranad et al. 2001; Zhang et al. 2013); however, there has been no report of larval settlement and metamorphosis. Although this process is well understood for many commercially important molluscs, i.e. GABA (gamma-aminobutyric acid), diatoms or pregrazed conditioned plates are most commonly used to induce abalone larval settlement in hatcheries (Freeman 2001), the factor(s) required for the *C. tritonis* life cycle transition are still to be revealed.

Advances in ‘omics’ technologies devoted to gastropods (Klein et al. 2019) provide an approach to broadly investigate the molecular machinery that regulates physiology, including identifying genes involved in development and adaptation. In *C. tritonis*, an in silico neuropeptidome of the central nervous system (CNS) has provided an overview of all neuropeptide precursors (NPPs) present in adults, some of which are known to regulate growth and reproductive processes (Bose et al. 2017a). Transcriptomics also provides the means to explore the *C. tritonis* G protein-coupled receptor (GPCR) superfamily, which includes those that bind neuropeptides (NPs), as well as the various environmental chemical cues that are critical for predation and larval settlement (i.e. olfactory receptors). Regarding predation, a transcriptomic-proteomic analysis of the *C. tritonis* salivary gland has revealed putative venom and feeding-related proteins, providing insight into the source of bioactive components used by the snails to prey on adult COTS (Bose et al. 2017a, b). The molecular basis by which *C. tritonis* can detoxify the COTS toxins (e.g. saponins) could be explained through analysis of cytochrome P450 (CYP450) enzymes. CYP450 enzymes are well known for their roles in the detoxification of foreign compounds (Hannemann et al. 2007; Isin and Guengeric 2007; Mansuy 1998).

This study aimed to provide a more comprehensive gene resource that could facilitate further targeted investigations into the mechanisms that promote and regulate *C. tritonis* life histories. We present the development and overview of this resource, as well as major findings into the identification of GPCRs, NPPs and the CYP450 superfamily.

## Materials and Methods

### Broodstock and Animal Maintenance

*C. tritonis* were collected within the Great Barrier Reef under permit (G13/36390.1 and G17/38293.1) at depths up to 30 m by Cairns Marine and Pacific Marine Group. Specimens were held in artificially aerated containers and transported to the Australian Institute of Marine Science (AIMS) aquarium facilities. At the time of collection, all individual specimens were measured by weight and length of shell. At AIMS, *C. tritonis* broodstock (*n* = 5) were maintained in a round 4000-L flow-through tank with a flow rate of 5.45 L/min (equivalent to 0.64 water exchanges/diurnally) of ambient filtered seawater (FSW; 1 μm, temperature ranging from 30 °C in the summer to 23 °C during winter, salinity 32–35 ppt). Broodstock individuals were fed live adult COTS, which is the preferred diet for *C. tritonis* (Paterson and Poulsen 1988). Occasionally, alternative echinoderms such as *Linckia laevigata* and *Strichopus chlorotus* were offered.

Mating was observed in the summer months (December–February) with egg-laying occurring from April to May (Hall et al. 2017a; Motti et al. 2018). Egg capsules containing pre-hatched individuals were left in the broodstock tank without human intervention. At 50 days post-fertilization (dpf), capsules were manually cut opened with a sterilised scalpel blade and veliger released into a sterile environment to avoid introduction of biological contamination, as well as to control hatching time. Newly hatched larvae were transferred to culture vessels; circulation was provided by introduction of 26 °C 0.45 μm filtered FSW from the base of the vessel and aeration via a tube positioned ~ 10 cm below the water’s surface. Moreover, all culture vessels were individually dosed with a microalgal mixture (*Dunaliella* sp., *Isochrysis* sp., *Pavlova lutheri*, *Nannochloropsis oceania* and *Chaetoceros mulleri*) at a concentration of 3000 cells/mL using an automated feed pump (dosage varied from 4 to 0.4 ml/min depending on feed density required). For further details pertaining to larval rearing, refer to Motti et al. (2018).

The health of post-hatched veligers was monitored daily using a stereomicroscope (Leica DFC 5500), and their activity, morphological development, and heart rate were recorded. To sort living veligers from dead, the neurotransmitter serotonin (~ 10 mM)—known to significantly increase locomotion in healthy veligers (Penniman et al. 2013)—was added (100–300 µL/L). Active swimming veligers were siphoned into a new culturing vessel, non-mobile individuals that had sunk to the bottom of the vessel were removed.

### RNA Isolation

Encapsulated *C. tritonis* larvae at 25 and 50 dpf (*n* ~ 1000 each) were released from a single egg capsule using a sterilised scalpel blade, directly into RNAlater (Ambion). Free-swimming veligers at 14-day post-hatch (dph; *n* ~ 500) were collected via siphon over a 100 μm filter and immediately transferred into RNAlater (Ambion). All samples were stored at 4 °C. These developmental stages were selected as they provide ideal temporal and morphological separation. Foot, gill, posterior and anterior salivary gland, CNS, proboscis, tentacles, and mantle tissues of *C. tritonis* adults, previously collected and stored in RNAlater at 4 °C (Bose et al. 2017a, b), were used for RNA isolation. RNA isolation of 25 dpf, 50 dpf, and 14 dph veligers and adult tissues was performed with TRIzol reagent (Sigma-Aldrich, MO, USA), as per supplier’s instructions. Total RNA was quality assessed through 1.6% agarose gel electrophoresis, and further quantified using a Nanodrop 2000 (Thermo Scientific, MA, USA) spectrometer at wavelengths 260 and 280 nm and thereafter stored at -80 °C until further use. RNA integrity was analysed by Novogene (Hong Kong) or the Australian Genome Research Facility (AGRF; Australia) for library construction and sequenced (150 nucleotides paired-end reads) using an Illumina HiSeq 2500 sequencing platform. RNA samples having a RIN (RNA Integrity Number) value > 8 were deemed of sufficient quality for sequencing.

### Transcriptome Assembly and Quantitation

The 11 RNA-seq libraries (3 developmental, 8 adult tissue) contained approximately 525 million raw reads with a mean length of 91 base pairs (bp) combined (> 40 million reads per sample). Reads with adaptor contamination, or when uncertain nucleotides constitute more than 10% of either read (*N* > 10%), or when low-quality nucleotides (base quality less than 20) constitute more than 50% of the read, were discarded by Novogene data filtering. Quality of raw reads of each library were checked separately using FastQC (Brown et al. 2017), and trimmomatic used to delete the 14 nucleotides at the extremities of each read (Bolger et al. 2014). Trimmed reads of the 11 RNA-seq libraries were merged prior to assembly using Trinity (Grabherr et al. 2011), which applies a de novo reconstruction method. The guanine-cytosine content (GC-content) was calculated to represent the percentage of nitrogenous bases on an RNA molecule that are either G or C (from a possibility of four different bases, also including adenine and uracil in RNA). Quality of the assembly was assessed using the built-in Trinity Perl script to generate an N50 value. Alignment coverage rate was calculated using the program Bowtie (Langmead et al. 2009) with a cutoff set at 70%. The level of completeness of our reference transcriptome was evaluated using bench-marking universal single-copy orthologs (BUSCO; v2.0) (Simão et al. 2015). Gene expression levels were calculated using RSEM software by applying the transcripts per million (TPM) method. The formula applied was FPKM(A) = (1,000,000 × C)/(N × L × 1000), where FPKM(A) is the expression of gene A as fragments per kb per million fragments, C is the number of fragments that uniquely aligned to gene A, N is the total number of fragments that uniquely aligned to all genes, and L is the number of bases on gene A. Following assembly, Transdecoder was used to predict open reading frames (ORFs) with a selectable minimum default parameter of 100 amino acids ORF length (https://github.com/transDecoder/TransDecoder/wik). In addition to the reference transcriptome (i.e. all 11 libraries combined), reads from each sample were assembled independently as quality control for the reference transcriptome.

### Biological Annotation

The BLASTp was performed against the NCBI non-redundant protein database for gastropods to obtain biological functional annotation relevant to *C. tritonis*. In order to increase the quality of the annotation, HMMER, a protein-family based annotation tool, was employed to search for all Pfam protein family profiles (Finn et al. 2013). Then, BLAST2GO pipeline was used to obtain the gene ontology (GO) annotation for each transcript (Conesa et al. 2005). Finally, transcripts were mapped to the KEGG database for annotation to predict their metabolic pathways. From each tissue, putative GPCRs were extracted using their Pfam accession number and prediction of transmembrane domain (TM) performed using TMHMM. Only GPCRs with a minimum of 5 TM were retrieved for further analysis. GPCRs were classified into families using SeQuery tool (http://cluster.phy.ntnu.edu.tw/) (Hu et al. 2019). According to the classical A-F system, GPCRs can be grouped into 6 classes based on sequence homology and functional similarity (Attwood and Findlay 1994; Kolakowski 1994; Davenport et al. 2013): class A (or 1) (rhodopsin-like), class B (or 2) (secretin receptor family), class C (or 3) (metabotropic glutamate/pheromone), class D (or 4) (fungal mating pheromone receptors), class E (or 5) (cyclic AMP receptors) and class F (or 6) (frizzled/smoothened).

To investigate candidate olfactory receptors (ORs), a list of ORs from *Aplysia californica* (Cummins et al. 2009) were blasted against proteins from the newly assembled reference transcriptome of *C. tritonis* with an e-value cutoff set at 1e − 5. ORs found in the tentacles of both species were used to build a phylogenetic tree (MEGA7). NPPs derived from the molluscs *Lottia gigantea* (Veenstra 2010), *Pinctata fucata*, *Crassostera gigas* (Stewart et al. 2014) and *Theba pisana* (Adamson et al. 2015), as well as from a previous study of *C. tritonis* (Bose et al. 2017a), were used in a BLASTp search (e-value cutoff set at 1e − 5) to identify homolog proteins from our *C. tritonis* samples*.* Matches were manually assessed against known NPs to determine conservation of putative bioactive regions.

The reference transcriptome of *C. tritonis* was searched using the hidden Markov model (HMMER v3.3) (Finn et al. 2011) and putative CYP450 proteins identified using the global PFAM model for (CYP450) accession (PF00067; only full-length sequences and partial-length sequences above 250 amino acids were considered) (http://pfam.sanger.ac.uk/). Putative CYP450s were named based on the homology with CYP450 from the Cytochrome P450 Homepage eukaryote databank (Nelson 2009). Values of 40% and 55% amino acid identity were used to assign CYP450s to families and subfamilies, respectively. These *C. tritonis* CYP450s families were further divided into clans similar to those in vertebrates. The CYP450 families were then identified using BLASTp. If identity was above 30%, and typical conserved CYP450 motifs important for catalytic activities (Rewitz et al. 2006), including heme-binding region, PERF motif, K-helix region, I-helix region and C-helix, were present, then a similar function was assigned based on categories used for humans (Mittal et al. 2015). The deduced amino acid sequences of *C. tritonis* CYP450s were subjected to Multiple Expectation Maximization for Motif Elicitation (MEME, http://meme-suite.org/) (Bailey et al. 2009) analysis for identification of conserved motifs. The consensus sequences of the heme-binding region, also known as ‘P450 signature’, is ‘PFXXGXRXCXG’, and for the PERF motif ‘PXXFXPE/DRF’. The consensus sequences for K-helix, I-helix and C-helix regions are ‘EXXR’, ‘GXE/DTT’ and ‘WXXXR’, respectively. Sequences of the 5 conserved CYP450 motifs were extracted and then subjected to WEBLOGO (http://weblogo.berkeley.edu/) (Crooks et al. 2004) to create the sequence logos.

### Veliger Heart Rate Assay

Identified NPs were assessed for their ability to regulate *C. tritonis* veliger heart rate (beats per minute; bpm). FMRFamide, FFamide and egg-laying hormone (ELH) were synthesized by ChinaPeptides Co. Ltd. Veligers (*n* = 10) were added to each well of a 6-well plate containing 10 mL FSW. Veliger heart rate was observed under a microscope for 3 min prior to addition of treatments (10 μM). Serotonin (known to accelerate ciliary beating rate) was used as a positive control, while FSW was used as a negative control. Significance was estimated based on adjusted *P* value using the Bonferroni correction with a threshold set at *P* < 0.05. Standard deviation and *P* value was calculated using R (version 1.2.5033).

## Results and Discussion

### Assembly and Annotation of the Charonia tritonis Reference Transcriptome

In molluscs, reference transcriptome databases have proven useful for providing insights into the molecular machinery that governs early life-stage development and adult physiological processes. For example, developmental transcriptomes of the gastropods *Rapana venosa* (whelk) and *Aplysia californica* (sea slug) have enabled identification of genes associated with transitions between life stages (Song et al. 2016; Heyland et al. 2011), and as more datasets become available, comparative analysis can establish evolutionarily connectedness (De Oliveira et al. 2016). Here, a reference transcriptome for *C. tritonis* including combined early life stages (25 dpf, 50 dpf, and 14 dph) and various adult tissues, and also separate assemblies per sample, were established to facilitate the subsequent targeted interrogation of genes (Supplementary File S1).

**Fig. 1:**
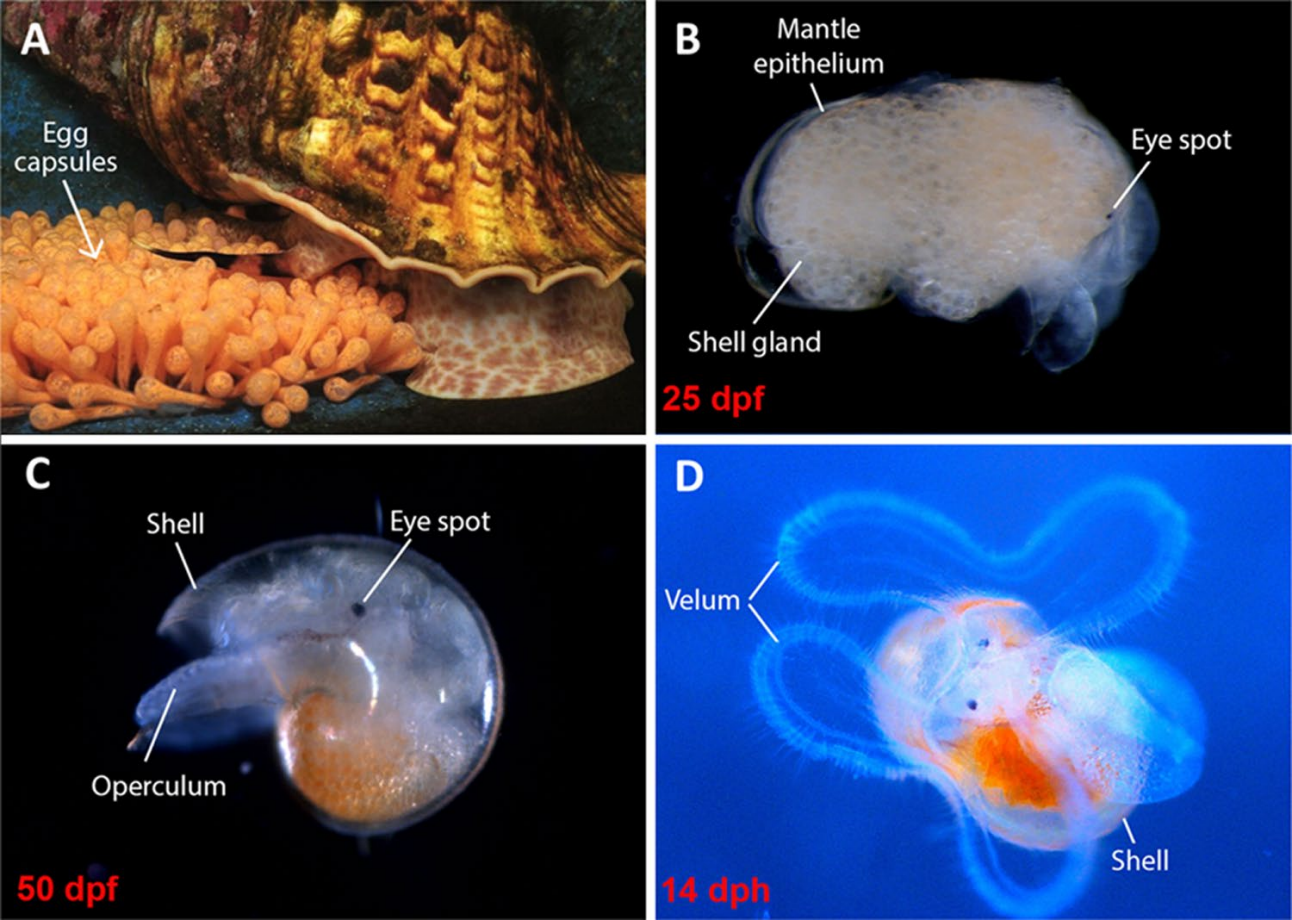
Photos showing *Charonia tritonis* life stage. **A** Brooding female guarding recently laid intensely orange-coloured egg capsules. **B** A single yellow/opaque encapsulated larvae at 25 dpf. **C** A single encapsulated protoconch I larvae at 50 dpf. **D** A free-swimming veliger at 14 dph

In *C. tritonis*, the temporal and anatomical changes associated with embryogenesis and early larval development have been described (Berg 1971; Nugranad et al. 2001; Zhang et al. 2013). Embryogenesis up to a late larval (protoconch I) stage occurs within the egg capsule (Fig. 1A) and has been reported to be in the order of 35 to over 60 dpf (Zhang et al. 2013). At around 25 dpf, we observed the protoconch shell and the beginning of eyespot formation (Fig. 1B). By approximately 50 dpf, the protoconch shell, operculum, foot and right tentacle were readily distinguishable (Fig. 1C), then at 14 dph, hatched free-swimming veligers were observed with one pair of velar lobes (Fig. 1D).

**Fig. 2:**
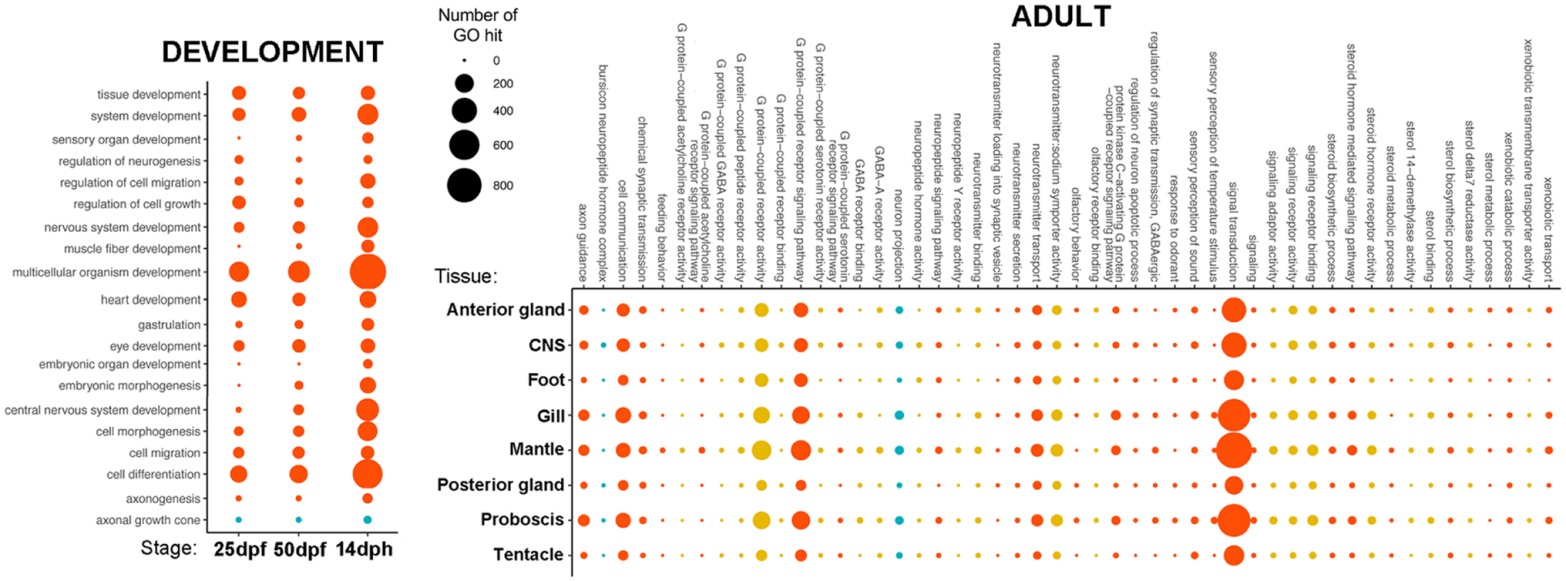
*Charonia tritonis* gene ontology (GO) annotation for developmental and adult tissues. dpf, days post-fertilisation; dph, days post-hatch; blue, cell compartment; yellow, molecular function; red, biological process

Paired-end sequencing of RNA samples from developmental and adult *C. tritonis* tissues generated 120-Gb raw data in FastQ format (GenBank accession number PRJNA699909). Subsequent de novo assembly generated a total of 613,506 contigs, including isoforms, and 61,611 proteins. To facilitate discovery analysis, small proteins having a minimum length of 100 amino acids were included, giving a final N50 value of 556 bp, L50 (corresponding number of sequences) of 126,520 and N90 of 235 for this reference transcriptome. This is similar to that reported from the neural transcriptome of *C. tritonis*, with N50 of 641 bp (Bose et al. 2017a). The Bowtie coverage rate of 81% lends confidence to the assembly quality. To further evaluate the completeness of the reference transcriptomes (combined and individual), proportions of complete as well as partial homologs of 429 conserved eukaryote genes were assessed. The reference transcriptomes contained 83% of the complete conserved eukaryote genes, and 6.5% of fragmented BUSCO genes that is comparable to other mollusc transcriptomes published recently (Ip et al. 2018, 2016; Sun et al. 2012; Mu et al. 2017; Bankers et al. 2017; De Oliveira et al. 2016; Gerdol et al. 2017). To guarantee the best annotation possible, a combination of tools using different bioinformatics databases was applied. BLAST, using the non-redundant database and GO, annotated 14,095 and 11,575 proteins, respectively, with an e-value threshold set at 1e − 5. A relatively high number of these proteins annotated to biological processes associated with signal transduction, GPCR signalling, cell communication and neurotransmitter transport (Fig. 2). This was supported by a relative abundance of proteins corresponding to the molecular function groups for signalling receptor binding, GPCR activity and NP hormone activity.

### Identification of GPCR Genes, Including Candidate Olfactory Receptors

**Fig. 3:**
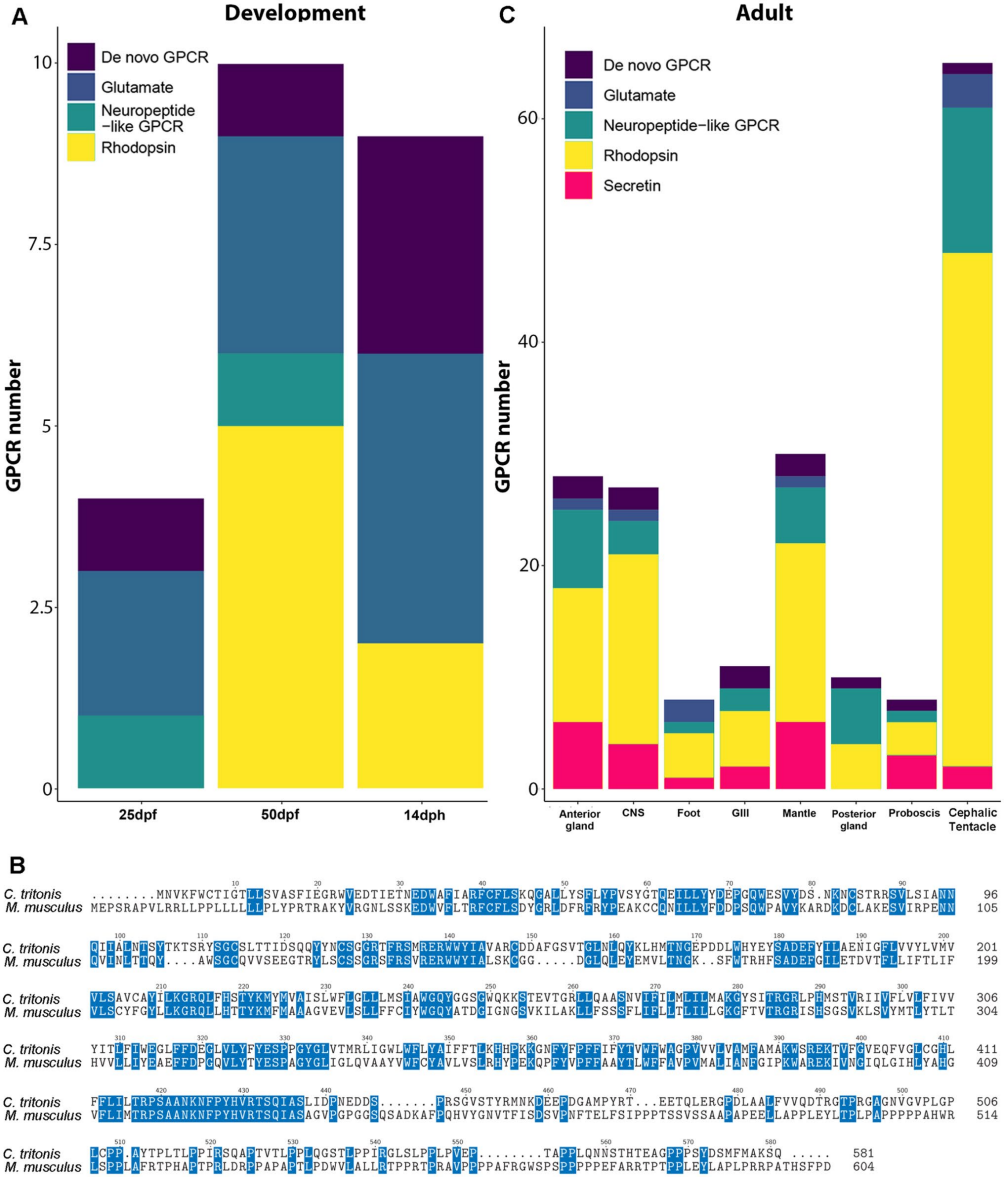
Classification of G protein-coupled receptors (GPCRs) identified in *Charonia tritonis* into different families: glutamate, secretin, rhodopsin, cAMP neuropeptide-like and de novo. **A** GPCRs present at different stages of early life development: 25 days post-fertilisation (dpf), 50 dpf and 14 days post-hatch (dph). **B** Multiple sequence alignment of a GPCR exclusive to veligers at 14 dph (TRINITY_DN337635_c4_g1_i1) with a *Mus musculus* homolog GPCR. Blue shading represents conservation. **C** GPCRs present within different adult tissues. See Supplementary File S3 for all *C. tritonis* GPCR sequences

GPCRs are 7 TM proteins that constitute a vast family capable of binding ligands and regulating a wide range of functions, including autocrine, paracrine and endocrine processes. They are also associated with larval/veliger growth and metamorphosis (Bai et al. 2011; Yang et al. 2014). Four classes of GPCRs were identified and categorised in *C. tritonis*, namely the rhodopsin-like, secretin and metabotropic glutamate/pheromone GPCRs (Supplementary File S2). GPCRs identified as ‘outliers’ by SeqQuery were, in fact, NP-like GPCRs, and those identified as ‘not GPCR’ were too distant from those present in the SeqQuery databank (primarily constituting mammalian GPCRs). In the three development stages, targeted analysis revealed that, overall, more GPCRs are expressed at 50 dpf and belong to the rhodopsin-like GPCR family (Fig. 3A). At 14 dph, the glutamate GPCRs were more prominent.

Comparing larval/veliger and adult GPCRs raises interesting questions about their functional capabilities, inclusive of those that may regulate settlement and metamorphosis. Eight GPCRs found in the developmental stages were absent from adult tissues (Supplementary File S2). Of these, one is exclusive to 14 dph veligers and annotates to a GPCR that is conserved throughout the animal kingdom, named transmembrane protein 145. It is predicted to be involved in a GPCR signalling pathway in response to a pheromone stimulus (GO:0,019,236). Homologs to *C. tritonis* transmembrane 145 GPCR-like protein are found in other molluscs such as *Pomacea caniculata* and *Aplysia californica*, and even in mammals, where the *Mus musculus* homolog shows most similarity within the extracellular N-terminal region and 7 TM domains (Fig. 3B). Also of interest is a metabotropic glutamate GPCR-like protein (TRINITY_DN99081_c0_g1_i1) that was exclusive to 14 dph and the adult cephalic tentacle, suggesting a role in olfaction. Based on its e-value and query coverage from blast, this GPCR is largely restricted to molluscs. Metabotropic glutamate GPCRs are known to bind glutamate through a large extracellular N-terminus region and modulate neural synaptic transmission (Cochilla and Alford 1998; Cartmell and Schoepp 2000).

**Fig. 4:**
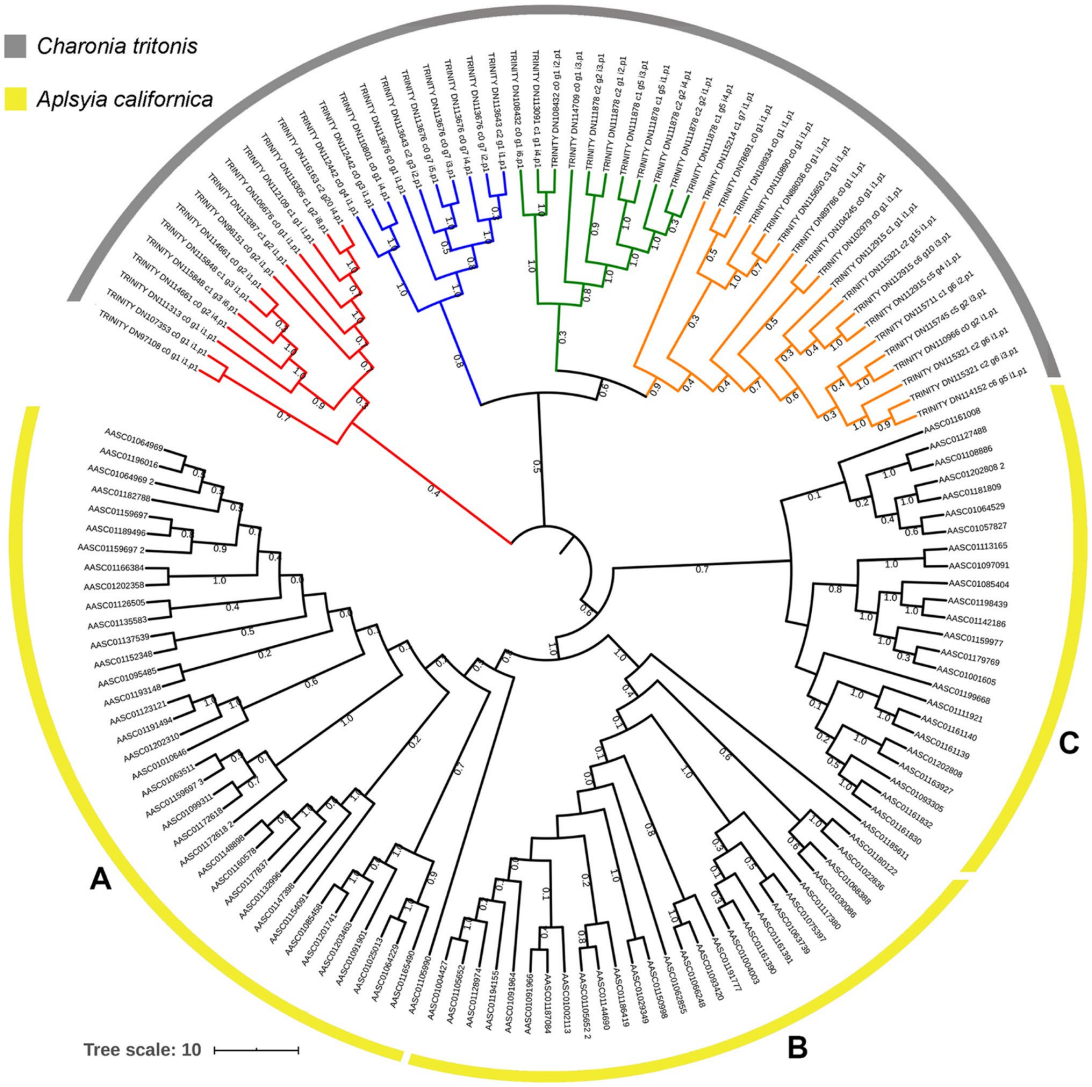
Phylogenetic analysis of candidate olfactory receptors (ORs) identified in the cephalic tentacle of *Charonia tritonis*. Phylogenetic tree demonstrates the clustering of *C. tritonis* candidate ORs, represented by red, blue, green and orange clades. *Aplysia californica* candidate ORs are grouped into **A**, **B** and **C**

Similar to other predatory gastropods, *Charonia* spp*.* are likely to use olfaction to locate prey, made possible by a pair of large sensory cephalic tentacles that facilitate directional odour detection (Hall et al. 2017a). Of the 143 GPCRs identified, 137 were identified in *C. tritonis* adult tissues, with the majority identified from the cephalic tentacles (Fig. 3C). Sixty-six GPCRs (48%) were present in the cephalic tentacle, including 61 exclusive to the cephalic tentacle. Most GPCRs identified were classified as rhodopsin-like. Rhodopsin-like GPCRs are well known for their role as chemosensory receptors, and have been implicated in olfactory processing in aquatic gastropod molluscs such as *A. californica* (Cummins et al. 2009) and *Biomphalaria glabrata* (Adema et al. 2017). Candidate ORs previously identified in the gastropod *A. californica* (Cummins et al. 2009) were utilised in a comparative phylogenetic analysis with *C. tritonis* cephalic tentacle GPCRs (Fig. 4)*.* Fifty-three candidate ORs, clustering into four distinct clades, were identified for *C. tritonis*.

### Identification of Neuropeptide Precursor Genes and Functional Peptide Analysis in Veligers

NPs are widely recognised in molluscs for their roles as neurotransmitters and neuromodulators (Cahill and Koury 2016), with many regulating key processes like feeding, reproduction and osmoregulation (Morishita 2017). Also, the processes of larval settlement and metamorphosis in marine molluscs are largely controlled by sensory recognition leading to activation of endogenous signalling (Scheltema and RS 1974; Hadfield 1984; Cahill and Koury 2016; Geraerts et al. 1991). For example, in *A. californica*, NP enrichment during the metamorphic stage has been observed (Heyland et al. 2011). Our in silico NP analysis of the *C. tritonis* reference transcriptome identified a total of 117 NPP transcripts (including isoforms), coding for 37 different NPP genes conserved with other molluscs, some of which appear to be developmental stage- or tissue-specific (Supplementary File S2).

**Fig. 5:**
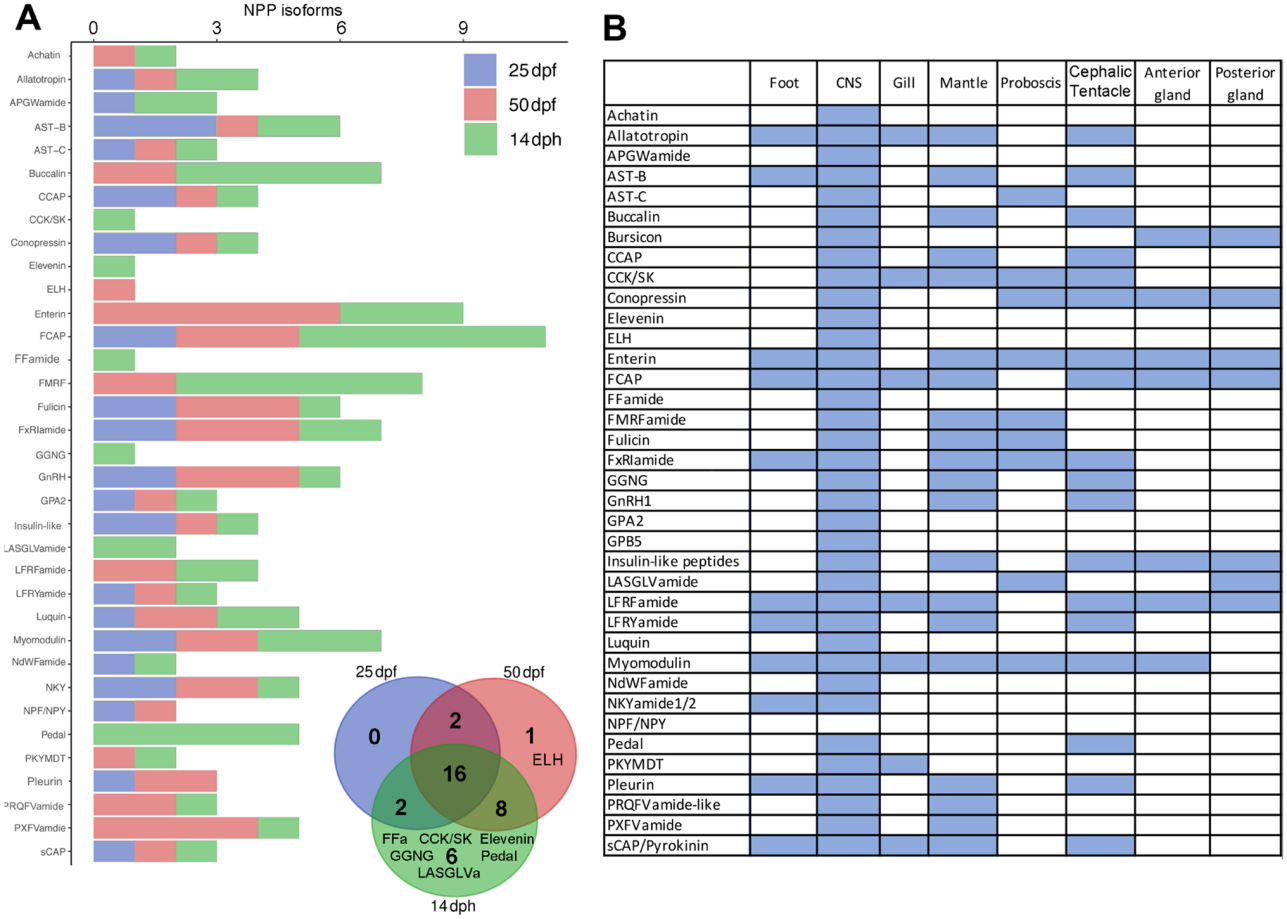
Summary of neuropeptide precursor (NPP) genes present in *Charonia tritonis.* **A** Graph showing NPP isoforms identified in early life development stages: 25 day post-fertilisation (dpf), 50 dpf and 14 day post-hatch (dph). **B** Summary of NPP genes present in adult tissues. Blue shading represents identified. See Supplementary File S3 for all *Charonia tritonis* NPP sequences

During development (both pre- and post-hatch), 35 NPP genes were identified, including 16 that were present at all three life-stages (Fig. 5A). Temporal change in expression of NPP genes was observed as development proceeded; 20 NPP genes were identified at 25 dpf, then increasing to 29 at 50 dpf (including achatin, buccalin, ELH, enterin, FMRFamide, LFRFamide, PKYMDT, PRQVFamide and PXVFamide). At 14 dph, a further six NPP genes were expressed, including the CCK/SK, elevenin, FFamide, GGNG, LASGLVamide and pedal, while ELH, pleurin and NPF/NPY were absent. The number of isoforms was investigated for each NPP gene and within each developmental stage (Fig. 5A). The highest number of isoforms was found for the feeding circuit-activating peptide (FCAP), FMRFamide, enterin, myomodulin and buccalin, particularly at 14 dph. Buccalin and myomodulin have been associated with the intrinsic modulation of accessory radula closer activity (associated with biting) in *A. californica* (Cropper et al. 1988), which may correlate with the requirement for *C. tritonis* larvae feeding (Kellett et al. 1996). The *C. tritonis* ELH gene was only found in 50 dpf larvae and adult CNS tissue. ELH is most often associated with the stimulation of egg laying or spawning in adult gastropods (Stewart et al. 2016; Cummins et al. 2010), as yet no function has been inferred for larvae or veligers.

In adult *C. tritonis*, all NPP genes besides NPF/NPY were found in the CNS, which also had the highest levels of gene expression (Supplementary File S2). Notably, the NPF/NPY gene was observed in *C. tritonis* larvae at both 25 dpf and 50 dpf, but not at 14 dph. NPF regulates swimming depth of larvae in the annelid *Platynereis dumerilii* (Conzelmann et al. 2011). Besides the CNS, the cephalic tentacle and mantle tissues contained a high proportion of NPP genes (17 and 18, respectively), which is likely due to their relative abundance of neural innovation. In the adult tissues analysed, FCAP, enterin, LFRFamide and myomodulin NPP genes were most broadly expressed across all tissues (Fig. 5B). Others were more spatially refined, such as APGWamide. APGWamide has been well studied in molluscs, demonstrating a key role in male copulation behaviour, for example, in the basommatophoran pulmonate freshwater snail *Lymnaea stagnalis* (Croll and Van Minnen 1992; Croll et al. 1991; Van Golen et al. 1995; Van Kesteren et al. 1995; York et al. 2012). It has also been detected in the CNS of other snails, such as *Fusinus ferrugineus* (Kuroki et al. 1990), the African giant snail *Achatina fulica* (Liu et al. 1991) and *Cornu aspersum* (Liu et al. 1991, 1993; Chen and Walker 1992; Liu and Takeuchi 1993, 1995). FxRIamide, GGNG and insulin-like peptide genes were found at relatively high levels in the mantle, while the foot contained a notable level of myomodulin. In molluscs, the insulin-like peptide has been studies in different oysters (*Crasstostera gigas* and *Pinctada fucata martensii*) where it has been associated with a role in controlling development, growth, reproduction (Moon and Choi 2020) and involved in cell activity, glycogen metabolism and other physiological processes (Zhang and He 2020; Antonova et al. 2012). Pedal peptides are known to be involved in the control of locomotion and ciliated activity in molluscs (Hall and Lloyd 1990). In *C. tritonis*, with the exception of adult CNS, pedal peptides are only found at 14 dpf, the latest stage of early life development examined in this study. A similar expression pattern has been reported in *A. californica* (Heyland et al. 2011), for which pedal peptides are highly expressed in larval and metamorphic stages.

**Fig. 6:**
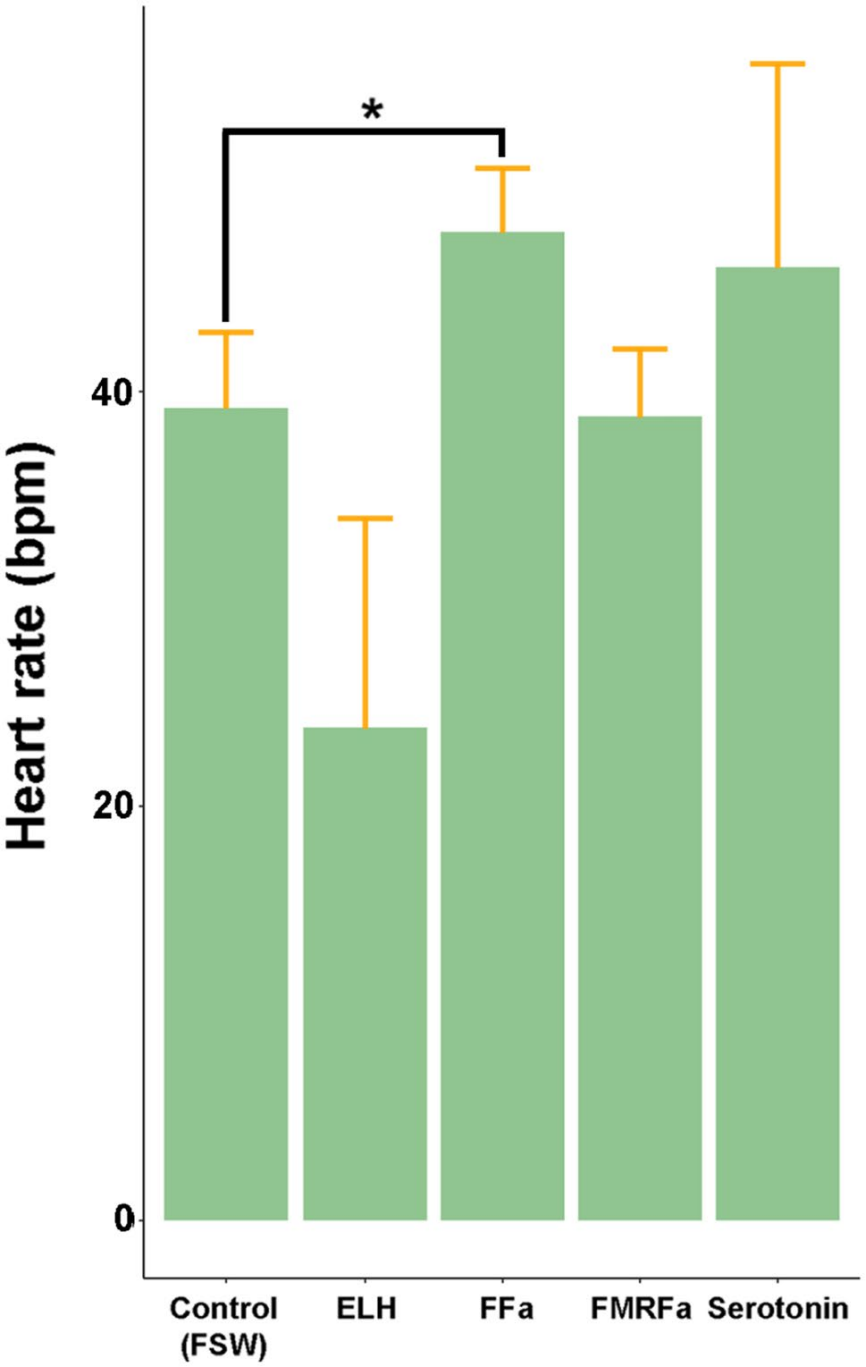
Response of *Charonia tritonis* veliger heart rate (beats per minute; bpm) to chemical stimuli. Neuropeptides and serotonin were administered at 10 μM (*n* = 10). FSW, filtered seawater. *Significance is *p* < 0.05

The identification of NPP genes and their distribution provides opportunities for functional analysis in *C. tritonis*. First, the neurotransmitter serotonin (10 μM) was tested; it can elicit increased larval activity through stimulating ciliary beating in the sea snails *Ilyanassa obsoleta* and *Crepidula fornicate* (Penniman et al. 2013; Braubach et al. 2006) and induce metamorphosis in competent *I. obsoleta* larvae (Couper and Leise 1996). In this study, it has been successfully used to separate live swimming *C. tritonis* veliger from dead in culture (see ‘Materials and Methods’). Furthermore, it was found to stimulate an increased heart rate beat frequency (av. 45 bpm), although not significantly (Fig. 6).

NPs derived from the FFamide, FMRFamide and ELH NPPs were tested in a veliger heart rate assay (Fig. 6). The FFamide NPP gene was only present in 14 dph veligers. Little is known regarding the function of FFamide in molluscs, however, neuropeptidomics of various species has proven its high conservation throughout molluscs (Stewart et al. 2014; Bose et al. 2017a; Zhao et al. 2016), suggesting an evolutionarily conserved and critical function. To date, it is known to play a role in the modulation of muscle contraction in male copulatory organs (Li et al. 1995) and increases in expression in the freshwater clam (*Corbicula fluminea*) upon exposure to organophosphate chemicals (Wang et al. 2018). Here, synthetic GINPNMNSLFFamide (10 μM) stimulated a significant increase in *C. tritonis* veliger heart rate. The FMRFamide has been more widely associated with the stimulation of heart rate, where for example, it can increase both the force and frequency of beating in *Lymnaea* (Buckett et al. 1990) and the crab *Callinectes sapidus* (Price and Greenberg 1977). FMRFamide is also known to modulate ciliary beat frequencies in *I. obsoleta* and *C. fornicata* larvae (Penniman et al. 2013), and its expression was detected in pre-hatched *A. californica* and *L. stagnalis*, suggesting a patterning function of the nervous system in these species (Dickinson and Croll 2001; Dickinson et al. 2000). Its presence (and relatively high number isoforms) in 14 dph *C. tritonis* supported a similar role. However, application of FMRFamide did not stimulate any observable change in *C. tritonis* veliger heart rate.

ELH (DISLNQDLKSLANMLLAREYDRILSNRMNREFLRKIGamide) caused a decrease (not significant) in veliger heart rate. Although ELH was only detected in 50 dpf veligers, its presence during development does suggest it is not strictly a reproduction-related NP. In support of this, ELH does have homology with a deuterostome stress response hormone (corticotropin-releasing factor), as well as arthropod diuretic hormone 44 (DH44) (Mirabeau and Joly 2013; Conzelmann et al. 2013), which does not stimulate reproductive behaviours.

### Identification of Cytochrome P450 genes

*C. tritonis* are one of the few active predators of adult COTS (De Lange et al. 1997), due in large part to their capacity to detoxify saponins, a family of defensive chemicals present within starfish and other echinoderms (Kamyab et al. 2019). The CYP450 enzyme system of molluscs (and other phyla) are widely involved in molecule biosynthesis and biotransformation of xenobiotics and are also key to many developmental processes (Jia et al. 2002; Rewitz et al. 2006). This enzyme family also contains excellent candidates hypothesised to be involved in the catabolism of saponins, as well as mechanisms for settlement and metamorphosis.

**Fig. 7:**
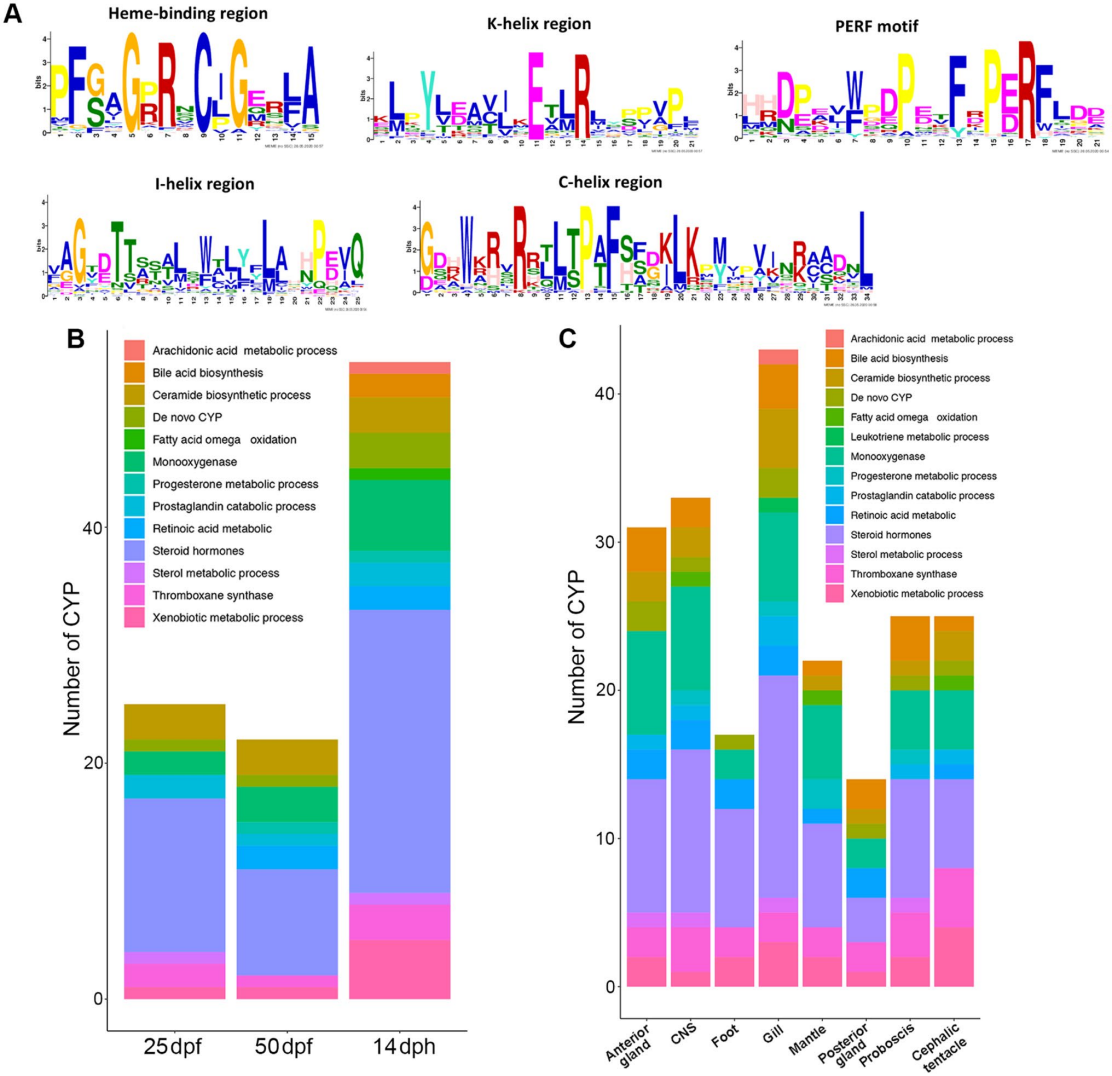
Summary of cytochrome P450 (CYP450) genes identified in *Charonia tritonis* and their functional classification. **A** Sequence logo representation showing conservation within CYP450 regions and motif. **B** CYP450 at different stages of early life development: 25 days post-fertilisation (dpf), 50 dpf and 14 days post-hatch (dph). **C** CYP450 within different adult tissues. See Supplementary File S3 for all *Charonia tritonis* CYP450 sequences

In this study, we identified 91 putative CYP450 genes in *C. tritonis* (Supplementary File S2), all encoding proteins with motifs consistent with CYP450 proteins, such as the heme-binding region and PERF motif (Fig. 7A), thereby validating our CYP450 detection approach. By comparison, the genome of *P. caniculata* revealed 157 CYP450 genes (Liu et al. 2018) that are mostly expressed in the hepatopancreas, gill and kidney. Furthermore, a genomic investigation of *B. glabrata* found 99 CYP450 genes, including those with adult tissue-specific expression; developmental stages were not investigated (Adema et al. 2017). We found that 59 *C. tritonis* CYP450 genes were expressed during the three development stages, with the largest number associated with steroid hormone biosynthesis (Fig. 7B). The existence of steroid hormones in molluscs is far from clear (Fernandes et al. 2011); therefore, the potential function of steroid hormone metabolism associated with CYP450 genes in *C. tritonis* requires further investigation. In post-hatch *C. tritonis* veligers, three families of CYP450 genes were identified that annotate to roles in arachidonic acid processing, bile acid biosynthesis and fatty acid omega oxidation, all of which would be considered normal larval metabolic processes. Furthermore, the number of CYP450 annotated to xenobiotic metabolic processing drastically increases post-hatch, correlating with exposure to increased environmental stimuli.

Various classes of CYP450 are dispersed in all adult *C. tritonis* tissues examined, with the highest representation being within the steroid hormone biosynthesis family. The gill presented with the highest number of CYP450 genes (Fig. 7C). The snail’s gill is a critical organ for biochemical exchanges with their environment, from gas exchange to filtration of particles and chemicals, including toxins. In fish, there is strong evidence pointing to a major role for gill-associated CYP450s in response to environmental stresses including water pollution (Uno et al. 2012). In molluscs, less is known, however, certain gill-associated CYP450s are differentially expressed in response to the diarrhetic shellfish poison okadaic acid (Chi et al. 2018). Unfortunately, our transcriptomes did not include gonad or digestive tissues (e.g. stomach, hepatopancreas), both of which would be considered key tissues for CYP450 activity during reproductive and detoxification events, respectively.

## Conclusions

*C. tritonis* is a threatened species which makes sampling difficult; therefore, the NGS-derived data we have obtained provides an important resource for molecular-associated discoveries that could help overcome bottlenecks in the establishment of aquaculture methods and a breeding program. Our reference *C. tritonis* transcriptome contains 613,506 contigs encoding 61,611 proteins with high confidence, of which 14,095 are fully annotated. We report 143 GPCRs, 53 being designated candidate ORs based on their expression in the cephalic tentacles. Many include rhodopsin-like GPCRs that are known to have chemosensory functions in other species. During development, GPCRs exclusive to veligers have been earmarked as being of particular interest for further characterization, since they could play an important role in detection of settlement cues leading to metamorphosis.

This study also provides a more comprehensive analysis of the *C. tritonis* neuropeptidome, leading to the identification of 117 NPP transcripts. Although much is still to be learnt about the function of NPs derived from these precursors, their identification enables more targeted functional experiments in the future. As a prelude to this, we tested some NPs on veligers and observed a significant increase in veliger heart rate with FFamide. We report 91 CYP450 transcripts, of which their presence was more prominent at post-hatch, likely an essential requirement for survival outside of the egg capsule. Similarly, in adults CYP450 abundance in the gill is more pronounced which may reflect its function in detoxification.

## Supplementary Information

Below is the link to the electronic supplementary material.

Supplementary file1 (DOCX 109 KB)

Supplementary file2 (XLSX 10 KB)

Supplementary file3 (XLSX 70 KB)
