# Supplementary material for: Development and Interrogation of a Transcriptomic Resource for the Giant Triton Snail (Charonia tritonis)
Source: Mar Biotechnol (NY). 2021 Jun 30;23(3):501–15. doi: 10.1007/s10126-021-10042-7 (PMC8270824; doi:10.1007/s10126-021-10042-7)
Supplement: Supplementary file 1 — Supplementary file1 (DOCX 109 KB) [file 10126_2021_10042_MOESM1_ESM.docx]

>TRINITY_DN145875_c0_g1_i1

TQSWAMLAAHNLSIEEVLLSSGLGSNSRWFDPMTELCLVLAFCLSILLGLLGNGLVALML

CRRRHLQSARHCYLLNLVVSDILTCVLCVPFTLVRLTLKDWRLGELLCRVAPFLQLTYVF

VSIFTILAIAVERYRSIVCSTHLKAERHRLARVIIPSIWLLAVGLATPLAVTHRLEHVYN

VTGEGVLLTLCVEDWDSDTWLGVYTVLVLLFQYVLPAVAIMTLHLLICRFLRTRVHLRAD

SVRSRQKLARHRKNLVLLTVISITFDLEWLPITVVNIVADFDPTLFPSAQHFCLTYSLCL

LFALTSVFVNPVVYGWYNSNFRREMCGTCNRETASERSGSTARGSSGAVSRFFSLIVRSS

AISRPQDSVDKCGTGSPPRPPHARLVRHHSSLDATHNVWPFTRHARMRAQRMSLQFELK*

>TRINITY_DN149433_c0_g1_i1

MSSSRPGSGTDQQWRNWEQEQFIRDLHKASNILGMSLLVVSLTAVLLTMVVYSCFVTLQC

MRVTLIKNLMVAILFQEHITLVLQALVFFARKENLPDIRCLFRNTSFCEALIAGSKYFEL

TTLSWLAVIAYNQWSRSKPKLNKRFNFVLLSLIGWISPLFPVAVWTGLMAQRPQANAGEG

SIRCLRSPFWKVRNSSISSSL*

>TRINITY_DN18631_c0_g1_i1

MNKTEEENFTGEELPIWALVVFGLLHSLTSVNGVVGSVLILTALTTNKHFRTSTNCTYAV

YMGNLVVTDLYFQIYFFPMLAVGFALGRYPVVNSAHCVVTGYTAMCCYSVFILTLTAISF

DRYIRVCHDNLHRQYFSWKTSLAVCAIIWILGAMVPLTGALKNSLGFDTKTWVCFTKHSA

DASVSLPYLMCFVFTMLASTFFNLRIYMVYRATRRRIEQHAQAGQDTEAFPRQKTVSSSD

VALLRSLLVIILCLVIFVTPGSVARGLRSKVDIDNVLYAFFLWLLSLNSSIDWIVYGLLN

TRFRIGYRNILSKCNLPP

>TRINITY_DN186513_c0_g1_i1

LTLSEAAHEGNNASAKRRRTASSTMLSQTAATTAEELVPLSNDSYKSNCSVLDLIGEPAA

GVNKSTSAATVEEYTDAEKYIWVIGGFVLLLVGTIGNVLALLVLCRPRMRKQKASIYLIV

VAVTDLAVLYTGLLRQMLLKAFHIDVRHQSELSCKLHTTLVYFFLDLSAWVLATLSLERA

VSVRWTLAVMRRCSRLTSSAIIAGIAVFLFAVNSP

>TRINITY_DN191157_c0_g1_i1

MWNLIVLAYGSSSPALSNRERFPMFFRTHPSGTLHNPTRIKLFQLFKWNRIATIQETQEI

FTSTIEDLEKRVKEVDIDIAVRQSFLTDPTNAVRNLKRQDARIIVGVFYEDMARRVFCQA

YKEKLYGKKYVWLIIGWYPDNWYRANDDRHNCTAEQLEEALEGHLTTEAVILHQEPTMTD

VGMTAKQFTEQLNEVLNTTDPILITGYPEAPLAYDAVWALAFALNKTAVRLAKKGMRLED

FDYYNEEIAQEIYSAMNSTKFLGISGNVAFSSQGDRIAWTQIEQMRNGTYHKLGFYDYVA

DNLTWFGTDKWTGGKPPPDHTRVIDHLRVVSQTLYFAMCALAAIGIIAGVACLVFNYRNR

QRRCVALSQPNINGLTVVGCMLCLSCIFLLGLDGKFVSHDVYPFVCQVRAWFLSVGFTLS

FGSMFSKIWTVHQLATARKKDRKGVQIWELYTVLAVLLLLDVAVLVAWQILDPLKRDLET

FAKEEPTNTEEDIQLRPQLEHCISDNLSVWLGVLFGYKGILLIFGIFLAYETRSVKLKQV

NDSRFVGMSIYNVVVLCIITAPISLIIGNQQDATFAFVSLAIVLCSFLSMGLIFVPKMME

LAKHPQRDGQEVRGLTDSLVSREEEERHQRMVCENEQLKRQIAEMEERVKELNRRIQKKM

QEQKNFSGNTDSDIASPCYTSLSTTTTITTASITTTASTTTPKLGMKQGGLTIQPETHFQ

LDDRMITADNNSDSGLASWSNTRSPKASATDLEPFC*

>TRINITY_DN225040_c0_g1_i1

MAPVKNNQLAAQGAITLELQSTPIEKFEKYFVNLNPRSNVRNPWYKEYWEEVHKCSWFKS

SDGTRQCSGNERVNRRLDNQESKVQFIYDAVYALAIALHRMQEDLCGPRAWGVCNRLKTI

DGERLLKDYLLNISFRDGYGATVQFDEKGDAPGRYTIMNYQRNRHTREYEYKVVGTWADG

FLSLDLHKIVWAGGTMDIPRSRCSEPCKEGEIKNMQKGEQCCWICTKCSPWEYIKDEKTC

EKCELGLWPYDNKTGCYQLEIQHMNWGSIYAIVPMVLAGIGICSTMFVIILFILFNSTPV

VMASGRELSYMLLSGCVVCYFMTFILLATPSVVACAAQRFGVGFGFSIVYSSLLIKTNRI

SRIFESARRSAKRPPFISPKSQIVMTCVLIVIQILFTSVWLLMEPPGTRLHYPETREPIV

ILKCSSDDFSFLVSLVYNILLIIICTVYAVKTRKIPENFNESKFIGFSMYTTCIIWLAFV

PIYFGTLNSFRISVQ*

>TRINITY_DN231904_c0_g1_i1

CGGGGGYHVNGMSNVTDDVVALVTGLIDTVTPASFRQANGSRSFFTMSPNHNDSNTTSNP

TPAQRPESPYEVWAQVVIALILGALTLCTIIGNCLVCISVAIVKRLQTPSNLLIVSLAVA

DLLVAILVMPLTATMQIYGAWVLGPNVCDMWTTTDVLLCTASILNLCMISVDRYLVITRP

FQYAMRRTPKRMAMMITIVWVLSAVVSIPPVFGLKSPHQPYNCYISLDIGYQIYATLCAF

YLPLIVMIFVYFKIWLVSSKI

>TRINITY_DN240749_c0_g1_i2

RLKSFASAMTMTHEASSALSSTTTPSSSSSLNDTTALLVHIISSQSTAHGNHNHLDHDQN

ATDETVWTVLERLNHERAVYFLPAVIWVVLLMVVGVIGNVLVIYVYRRRFKRTSSNYFIL

TMAIFDLVACLIGMPTEIYDLLKPFTFYSGFGCKLFRSTENFTIYGSVVVLVEIAFDRYF

KICRPLMVVSLFKIKVLCAVAVFVAILMAVPSAVLFGITRISTPEPDIKGYDCSVSDTYR

KTAFNSAYYICLTVVFVVTLVILTVLYVRIWLELRQRRRMVIGDQLTKPKDQEELAQAKK

FRVSNDHDPRSLLRPLLHHHPFFLLLPERHHGSSGAHHLLPVHRPW*

>TRINITY_DN251981_c0_g1_i1

KEFPHALLKNLSRMTTLYADNFKLCCGQTLPDAFNVKDCHAPSPVFSSCQHLLGPDVQRG

LVWLLAPANLIGNVLVLVLTLRQRQEEIVQVFVSHLCGSKSVMGLYLGLLAVADQVWSGS

YLWHDTAWRSGTVCKVCAFFFLLSCQVAVFLQVAMTLERGVALTWPLKVGGGRRRRMVSP

VCVLAWLTGGILGVVPALFPGDSFSTTALCLPLPVSGESSEEHLYTLALLAVLNPTLMSL

TAGVQIYIHTLVRRNTLAFLADEVGKAHELTTARRVISLATMDACCWGLVTLWAVLTSQG

AWLSGDVISTASVFFVFVSSTLTSYLYLYNGFLERRRQVQRQRLLKRLGYKTKAKEGKIS

SF*

>TRINITY_DN267234_c0_g2_i1

MSTLLNNSSTSGVSDGDTQDTTDPYLTWLYNMRYYLNVTCFAIIIILGNFGNIMTIVIMQ

KMKSGESTVNIYFTGIAIMDQIMINARTLTNWIEWTFEYNILDLHSVVCKIMIWIQTGSG

TIGSWFLVCLTIHRALSVVWPHRVDLLCTRRTVLMLMSGISVFYAVLYSHYFVGFDLIHS

YNQTAYCFIASPDYAVFFYDVFVYMEVLIYSVLPFACLSIANSVLIWKLKVSLGAARNNL

SQGSSEQIMAREKAVSSVTLTVIVVSVAYIVLTLPSAVFFYDILLCSFDKPV*

>TRINITY_DN285149_c0_g1_i1

MTSTALLQALSTTLSPHLSPGEMAATDALITLSPSTMGTMTTTAAPVLPPGGGRPPPPDY

VLHTQLVCDRVLLPLVVSFGIMGNILSLVVLTRKEMASPTNCFLTSLAISDMSLLLLQIP

MFFGLNAQVAATDSFKLFVRYYTVIMYVMTNVFLTCTSWLTVAVTVERFLSLRFMMHPRI

VCSIKRAKRAIIGIFFTSFLFHFSKFFEYVPNTDLTSPRSLLPTELSMNKTYDTVMHITN

ITVAALLPVVALVIFNSFLVYFLATHRRRMLKHKTGKAGLGVGNGGGGTTSVDMLHVSCV

VVAIVFVFVLCHSLGVFVALNIAVHGRYKIFSDHLFIAFKSINGLLVMVNSSVNFLLYCS

ISRKFRKMFSAIFCQHWLVKSSSWSLPVPLSENSMATAVSIRPAPNANGYIHPPNDTAKE

ASFVRPSPSSSSTSRSADQVDM*

>TRINITY_DN290182_c0_g1_i1

MAAKAAITLLTTTSISTSPANNPMVNSTTMNGTMKSSRWMKRNVPFWDAAFLTVKITGSL

ESILIIAVNLTLLSVILSSPLLRSRMRNHLVISVVIANLIVGILSSPFAVDATVRRKWVH

GCYLYVLLILLTVYVQNFVSVWGIVALLLHYLARLLRYEGPNWLGRLPASVQKAVPGLLI

ASPWIVSMVLLVPLVFGGLHKFVWAVWTYTTCPMVLQEWATFLLNCLSFFIPAVILVVLT

VLIIILHRRRSDKTESQSAMETGMKVVPDVGQELETCWVHVLVAVLTILMMGPEHSFLMS

RYQIRGPLKSIVIASLSVHLLSDLTPLAVALIWLLMLPDVRGRLLELLSKLPCNFHWWRG

RSPPDASGNTSIAPVAFRDLHDE*

>TRINITY_DN296971_c0_g1_i2

MMASALALSIDASINTEQNNIANNNSTFTSGSGHKDPHEAFYEQARFITGLVIYPIICIL

GLCGNILCIIVMSQRQMRSSTNVYLLSLAISDGVKLISDLLYFIVVLLYHVDSPSGNKAY

GCLYPYAHYIFNCSLCVSAWLTVSVAFERYIYVCHPTKVKTYCNIARARTVSFTVFFSMS

LLAIPYAMRYKTVEATSNKTGTKNWTLVVTELWQNELFALIYTWVQNFLRSIIPLLILIA

LNVTIIYGMRRCRIGRSKSGRRHRITIMLIFVILIFLICITPDAVMSTFFGMGYYEEAYL

QRGIREITDLLLLLNSACNFVLYCIFNTIFWKNFVFLFCRRCYPDPSITEDSNMRRLSLV

GRPGRSTLRRGSRRTSKTCNNGWTRGGSPLLDQKVKRQNV*

>TRINITY_DN302351_c0_g1_i2

MVHTDYVNMTSEPVTNYFVTWVATVPMVTVSLVTLTANGTVLVMFAIRPSLRRAKNAYLA

SLALADFLIGCYMPLLVLEEVGLMRVEGARACRTYLTTRYSLLYVSLLSVLLITLDRWWS

VRWPFSYRTRHTRRLAAYLIAALWTLSVALYAFPIFLWNPNTSTSVNAPLDPTLRTHHQI

PRSQPADPNTITSHRPRHSTAPDGSRILHPGECEVPYVLSFAWSALTCCVMYLLPLGAMW

ILNCSLYQKIRLRRSVEIRRSTSVTDMFFVTMKNSKPTIHITEAPSLVGPPSGNDLPTPA

SPLMPFRSGSPTHRSTPQLNNLHPGYATPTMGRRHSSAIVTLCPEQLEIRRQLSQRRISL

PDWALRGDWLPSNNSSRCRYVG

>TRINITY_DN306168_c0_g1_i1

MDSTTAMAETTLPDFISNLQSTTARLSMTSASPVHYDVSSTSEAQGMRMTSVSVMMMMVT

TTANGWSFANTSTSWSSMEDVEQFLQHKNDEFTMLVLPAMIFLAILFAVGVSGNCMVLYV

YNRKLQKGTIRWFVQALAIFDLLSCLVAIPGEIIDMRNNYTFGSSPMCKVLRSVSMFCTV

ASGMTLMVVAVERYKRICTPLRKQITPKGAQVIITVSTVAASICAAPACIIYGPQTIPTD

NPAINGTDCSTADVFMGTVIPLAFSSFQFLLFVLGALGLIVLYSLIGRKVWSHAHFRKMG

FRAGINRRMSFFFNGSECSSPTTDDTVFAFPVKRNIPAVVVVAEEDNSNTSHGNVDPNDN

NNVVHTKAEKVATVEGFPSEVEKMEKTPAYSEPCGDDIADRASMDTEKHYPEDEHSEPDT

LTPHDLSLVNDLDGREQENVTSLHQTDLPSGVAGQAREKLSLHNIGLANRLRPKETEMLP

PPHLGLSNEIDRQSPESLSLDHKGLANGLDQQQTSEPHQAALPNGVEKKVPEVLTPHPTG

LPNGLVRKVPETLTPHHPSGTVRPVSMEQPPTPTGNFPHTLDTFRRTKSTSRPISRDRVK

SGDSAVVRGRYRRRSTHHTQSDMRRFLHRSESVNSGTRRTTLMLFLITLVYLLSFLPYLI

LMIVKVLDEESLSGRGGGWELAHNILLRSYFINSMANPIIYSFCSRTFRKECSKVLHCRC

CRSPLFVY*

>TRINITY_DN312729_c0_g1_i1

MSSMTTAASPVVDENMSFFGDRLLLFNRVKPEVISVDKYVSIFIYCIGFPGNILSFIVWI

QKRMRHSSGCYLAALALDDFIFMSLHVIFELHMVWGWEPLNTPVFCQIFPIMFLCAQYLG

PLLVLGFTTERYISICHPFKREKYCTVKRAKLVIGILVASALCLSGVQGYFYTMQDRITA

NGTYEDSYECGPRPEVSRNGNTSLLILWNLSIEILTFLLVPLTVLVLNILVISEMRRLSR

TEQVTLSGHSQRTSATTVMLLAVSFYLILTTLPVTIAYTLFYEFEYDLSQVVSVNETLND

PMFQRWVNYELVLAAIKEFGTTQYAFNIIFYVITGKIFRKELKRLFAKVLCGNISLSISK

EYSSLRSSLRREGSKTKSTWVSINGHTQTKNNVKDTTETKV*

>TRINITY_DN314768_c0_g1_i1

MSSNSSGGLIQEFGGSFFHWVSVEVLPYILGSVLGICVLVGIILNIILLVVYGKRQLYKD

PTNDFIPQIAIVDLLASFIVVIPSSVAALLHEWPFPDTLCLMHGVLVTWFHLLSFALVIL

IFVERVVKAWNPNLHGNTFNTHTFVVIISVFTWAGDFLVAFLPILGIGSIAFNKYQSQCS

LEHDKHHISTHVIFGLGLYISPLVVIICFAMLIEKRRRNIVHERNEERNKLIDLNESGEE

KPEKWAESPTNLSGGDKGGKGTGSDAPKVIKNGPSKAWDKTSDKGKNQLFKKNPNNPAAA

GGKGEGPCAKKTSREEFLESYNKRMSRPTSTTLVSRMFRDTEEEKDHHQAMTALITWGVL

EICWVWYLVAAMLVMYSGTDVWGGWHVIGVLMGDLTYCIKPIVYLAHNRHYRKASKETMP

DAVREKADIITQKMSQAVEKMDNVLFHSPGAQKSKTQKLQAAIATQKAAMIWKRKAIGKF

GFGKKIADAGKTDASKAASDSATRPKPAASLSGSVVAAAALAQAEAAKKDPATRTKSPEQ

KLPPVPELLIPPGSAMSSLPSTRQGTAGTLNSSRPATKSSVLLSVGGSAGPASPNSLRPP

STARSSIPSGDDDLA

>TRINITY_DN316687_c1_g1_i1

ALHYCRMGSLRCLRNIIHCHLIVSFALKNVMYIVIHSMIKQIHGTNYEWVCKVKVTVINY

LHCTNFFWMFVEGLYLFSMVVWAFSANKIKHWHYIIVGWVLPVVITTAWALVKALHDDKL

CWLPSRETSYDYIMHTPVIIVLACNVFFLATIIWVLVTKLRASNSLETQQYRKAVRAILV

LFPLLGLTYLLMFYGPATDTELYRIFKYLNAVLQTLQGLLVAIFYCFLNGEVQMLLRKKL

SSIQDSRGLFTRHNTKSSFVGSPGRSSFHALSMTTCNGRHSFAARDKRGQSETTAAGFPP

DEEEAMRML*

>TRINITY_DN96151_c0_g2_i1

MAQMLTTAQRMHYAASAVVGPIFVALGMLGNILSIVVWSRRNMRSSTGRYLTALAVADSG

VLLWFILTDTVKMMHPEVVNSTAYAVFFAYLGYPFFFLWVICSIWFMVGVTIDRFIMVYL

ITKAKEYCSDRRVKFSIGTIGTMCFLINVPHYLSFTVDWDRGANGTGPALVKTEFQKGEA

GMAYEIWVHCIVLVLVPWVTVFTLNMLIISKIGKTNRKMSSTKTAQSADKSRQSENQITR

LLLIVTFTFLVLLVLQCVTQCFYAIMPEEFDKNIIDEAFAVGKLGAIINSSINFALYCVS

GRRFRQEMFKLLGCKGRHPGILSSTDHSSTSASGMTSVGTVGM*

>TRINITY_DN320900_c0_g1_i4

MAQMLTTAQRMHYAASAVVGPIFVALGMLGNILSIVVWSRRNMRSSTGRYLTALAVADIG

LLICFILTDTVQMIYPELEDSAVYAILFAYLGYPCFFLCFICSIWFMVGVTIDRFIMVYL

ITKAKEYCSDRRVKFSIGTIGTMCFLINVPHYLSFTVDWDRGANGTGPALVKTEFQKGEA

GMAYEIWVHCIVLVLVPWVTVFTLNMLIISKNYWHIEVNNDQVRSERPTAR*

>TRINITY_DN323918_c0_g1_i1

VQTCALPIWSELGPPFRLTVYSEEHLVGTSVVLGIMILCCIIGNCFVIAAVILERSLHNV

ANYLILSLAVADLMVAVLVMPLSVVAEISRVWFLHSEVCDMWISFDVLCCTASILHLVAI

ALDRYWAVTSIDYMRRRSARRILLMILMVWIVALTISIPPLFGWRDSSNDPDVTGQCIIS

QDHGYTVFSTVGAFYLPMFIMICIYSRIYVVARRRIRKDKFNKRKRNGASSSGAQTVATT

EQITLTVTVAPHPEYSVISNCNGCSPDKTLSPAKHHPSKNGVSATAMLGRDLDSPGGSAG

NGQTLGYTNGIELDPPTIMLDKAGGEGARKPCLPSSRQREKLEQKRERKAARTLAIITGA

FLLCWLPFFIVALSGPFIRGSVNIPPVVESLLLWLGYLNSLLNPIIYTIFSPEFRNAFRK

ILFGKYSRRGR*

>TRINITY_DN325019_c0_g1_i1

MDLLTTAIPNSNMSIDFAPLAPYQTAVLLWKVCSPIILVLGTFGNSMTLIVLHTMPSTKD

NVSVYFAALAISDLVLSYTGLFRRWLLYAMEVDLRNLHDVMCKLNLFLIYLSMMTSAWFL

VALTMQRVMSVLWPHRVALLCTKRKAQAIVVLIVFILVALNFYIPVFYTLQTNSNSGTPV

CELLDDPDLNNYDTFIYPWIDLVVTSLLPSSLLILGNSILAVSVFKSLRQAHQMTGQTHT

TRKKAVSSLTVTLICVSVTYLCLTLPICVYIIVHNFLDVGDDMHYQAWLELVDAICNLMS

YSNSAVNFYLYCLTGGKFRDQCSRILSCFSRCT*

>TRINITY_DN326447_c0_g2_i1

MSAREVHLVNLNSTFNQTQLSINLSACKYYHAPSRQCYVSDEHLKEEIWAYLQPSVTEWT

FLALYLLCFVLGLGGNGLVVWAVVRNSHLRSTTNVLLTNLALADFLAVLVCLPPNLVQTI

WETWFLGQTMCKLVEYYQVIVVLVSILTLTAISVERYCAICRPLTFKQTRSRVVICLVLI

WLAAHLAAMPRLFIMKLRHDQIVPPNVTIMLTSCVPSDSLKKVALHYEIFLCVVFYAVPI

VIMGFAYTAVALCLWASANTSFLTETDSNAILSQLRARRRTAKMLIVVVIVFMICFLPVY

IWNMIRLVSPQFLMYLNTDLVSAITLGAHLVLLINSCVNPLIYNFMSAKFRKEFQTACSC

LPCCSIPYDANNSYGRDRGLGLYPDSDGHICGAVLRHLSAADLQADEKSCCDLPRAHLAG

RAPRRHAPPLHHEITSRPDRAA*

>TRINITY_DN328585_c0_g1_i1

MSDVSNVVIHSSTFPSFTISDITSDIMIYSSTSPTFTSGGDKMTMTPSYSDTEVAEFLHQ

RNMDMMSRLTIYLVFLVSLMVCGLAGNSIVFAVYYYRFKPSNPRTFILAMSLCDLLTNVF

ALCYEMLRVRFSFTFHPWGCKVFAVAMTVVVNFSAFVLVAVALERKKMVCGNHVRSHAVD

RFVHVALFVCSFLAVAWAIPRAFLFGESTVRFPDTNITGVKCDIQDVYEHSLFRVVYTGL

LSLLFLMSVVIVAVCYVRIGIHLLRHKRKTYKSMKKAKSSTSSDIPLKQGTSNTSDMDSG

VSVDQKDSDLGKDLQDIHVVPTCNQTLTAPQSVLHESVTDAQQGQKRQTASEEATKMTLP

TTSTENLETSTKIKASLPTVSNEISGTSSKESSEVTKVTLPTESGETKTSTRAPAKLSGE

TFEISSTETKEATEAVPVTSTDAVEIKPSTRSPPKRSGGLGTPIPSRTTLMLFVFTVFFV

LNFLPYLTIQSMRSLAQESRLQAMDINLLVMCFRSFYLNSAINPFVYSFCSAKFRYECRQ

LARSLIQRWRRRERVVTV*

>TRINITY_DN97108_c0_g1_i1

MVEFQRSNLEMTTPDSTVKITSSSGLFTTSGPESHTKWPTPSYVASHMTVDSTTPVSTDP

HGHHPDSLFLGWVNMTDVRTAFDCGVIPVLSVLGIAGNCLCVVVFVKQRYRSVAKPLLLA

LCASDILFLLATFLVSLPCLVRKVNEKDGKMLHVHMVPQVDVFRDIMSRVTVMLTLAIGV

ERCVAVTRPLKLRGLCSIPRTRTAVLIIFLCVFALKSPAFFRYDVKRHVTPDNFTTVDVY

KTSFYLDNKNMLDFYFDYFLLVTLQGLPLVSIVLCLVIVLVNLKRRLQCAYPRTVAKFGA

FARARGKDQDKYKETEHADLVLEERKLTRALLVILLLCIISELPYLITEMYHVIRPDVQS

PSFLVARDVSRVLCVLNSAINFVVFMRLYKHFSVTFKTFAGLCN*

>TRINITY_DN102979_c0_g1_i1

MAVFYKQGLKERINVCLFALSLADFLLLLQTFILRSENMNTSRKERYGPIVTFLTNHNML

GLFGFAWVSNVISAIIASERCFCVLRPLLSRTLLRTSTITVIIVLVNVVTVGLYFFVAFR

YRIQCVFDPVRQVSFLSGVAGKFYVRHQKLLDYLDIFVYGVGLPGLVMVVSITTTIITTV

KLHQAAAWRAETSSASTNGMTSREIALTKMLVGNSVLFIACVFPVGLFRFVSLLVPAMNV

AGDQRNLYLVGIWLLDIFSCCNSTFNIIIYYTMGSRYRQTFWDLLGRGSKEERAVVVAAD

PGP*

>TRINITY_DN104245_c0_g1_i1

LKGAIILPLLFLIGGPANVINMVVFYRQGLRERINLCLFALSLMDGLYLCHCMVYNGETM

YFQFISSGERNGPFQGFLFNHNLVGFFGFTWTSQVISAIIACERCFCVVSPLRSQTILTT

RTMAILLIVVFVVVVGVYFLVVTRYRMVCVYDPVTGDRFSMLGGGPFYYKHQAFFDYLDS

MVYGVGIPGTTMLVVMVTTIVTAVKLRQAIAWRSETSGTLSPREVALTKMLIGNSVLFLV

CVSPIAIFKFVLLFIPDMRPGRRYQNLFFTFMWFLEIPSYINSSFNIFIYYSMGSKYRHT

VKALLCRTAGAKVKGKGGAEKRQPISHGSAVTCATSI*

>TRINITY_DN340691_c0_g1_i1

MLAAKFHDMFWNFQVSGPNIVQNKVAHRIMKSMPADFTSLLKMMASYSPYSHPNTLTTPF

SESSFTTPQTMDFTTTTLSSDNPDTTWMTEPDDRQCSSTDSFMPALPWIRIIFGMLMVVE

NCVSLVALWRVKRMQHAMHSFISSLAVAELLTGVWCCYRYGAEILVGVDMITVECRLRFV

GITYLNFVAILSISALCLDRCVALYFPFRYTELVSKRCVRMALAFVWTFPLLVVLTAYVD

TAHPDHPECSFVNIASQRTYIVLTVLRCCLITFIIVSELLIFRSAQCQIIKIYPSVFSSR

SSNRLLKLNAKAAITILAVVVPFLLLYLPVLLVQGNLAVRPHLADFCQSQLLAFVWLCAS

AHGLFTPLIFCWRFTEVRQNLLKILNCWTHFPRTSAVFHVRTNDSASTP*

>TRINITY_DN107353_c0_g1_i1

MQFTTSFPTTAFNWINFSEAAISYDVTTGETNATTNLPEAPEPSSFSGFVNDATKDAIVE

VVNCYLLPVVFVFGVSGNVMSFVVLLVHGMNNSTNVLLLGITVSDFCYLVTLYARKVSCI

VSHVDQMSSMILETTLIPNVHMVNRIFTTTTPFLTMLISLERCLAVTLPFKVQSIVTSFR

MKTVVVVIFLFSIAVQLPFFFIYEIQWTTDETTGQSIPKLKGTQFFYDNFQAITAYNNSV

LSAVFNYVPMGVIVVCTIVVLATIRKSARWRKAASQGGEGHEEGRMTKLLMTVVAVFICC

YVPRCAVLIANSLAPHSFSSMGGRYANLFSVFSALQFLLYSVNSSSNFVIYMIMSRRFLQ

TYTRIFGCRRRRGTRETSQNRLISSRIRVTSITSAATCEDANETT*

>TRINITY_DN89786_c0_g1_i1

CRVVLTQFTFIPWNNPDNIISRDTALRVVKVLEVILVPLLALFGMPANILCCVVFYRQNL

RDRINMLLFTLAVVDFSVEAYMLLHSIENLYTQILGRFDPYGPVMTFKTNNGLNVLYGFV

YASGFITTAIAFERCLCIGMPFMAKKVLKTKTAATIVVVVVAVLVGMHYIVAEKFKIGCT

YYPKMGFTLKGYYPSEFYMKNSKIIDALSSAVYGITLPCFFAATTIITTTITVIKLKTAV

TWRQQTSSLKREEKSKELALTRMLVAVSCLFIACTIPNIMVRIVPLPFPDFQLGGRYQNL

FLCGVCLSHFTPALNSSLNFLFYFHMGSKFRTTLTNLCCSSKKVLLEKSTSLSGTAMTTT

E*

>TRINITY_DN106676_c0_g1_i1

RLYDIHSVFCKVSFWICTGSGTVGCWFLVCMTSHRAVSVVWPHRVNSLCTRRTVLVLVTG

IAVIISALYSHYLFGCDLVSYDGGTTYQCMMETQEYINFVVDVFTYVELTVYSLLPFLCL

LCGNSILIWKLSSSVKDARKELGQGNSKQVLAREKAANSVTLTVVVVTMTFIVLTLPISI

NYVMSYFARVYNEVTGYEYAKAHLFYTVSYLLAYCNSAVNFY

>TRINITY_DN111313_c0_g1_i1

MATQPSSERTVTQGQFPDTKTSHDTLLKDQMTYFTDTQRVDAYNSLMIIRYVFLAISLPV

TVCNVIVFLQKRMRNATSVYILGLSFAQILFLSAHVGLSLLSGLDPEPRSTPSFCLYHLY

TISFGSVIARRGSYVILCFVSVERLYAILRPLHVKQFVLSRFSVTVMVATYVVTTIWHLY

VPAKLTVVMVEDEMTGTTLCRSHVTELYLRHKDINDTFSLAVKILMTYTALILQIVLNVL

TICALRRHNMAAKHVQPSANEEARRQRERQLTVVILATTIAYVILSLPACLNGLLNIVVS

EYNERGLHKNLANVVYNICLDLQVLSCSVDFFCFFALSSNYRKTFFSVFSCLRPVSLSKA

DLDTDTQNTLQASADSYSSK*

>TRINITY_DN111878_c1_g5_i3

SWRDPRNIMTYETYVFVMRIVELYIFPCILLFGVFTNILNAVVFFRQGLKDRMNLCLFWL

AMADLFFLLSVTPLASCSFAALVNPTYAREYEVNAFYYTVAIFTSFVHTSGCITAVIAIE

RCVCVVFPLKAQSLLSTRTMAFILLTITIFLQLSFSVFVLQSELTQIFDKNTGKTTWSFG

PSQWERQEIIFILYDVFFVLVQFTLPLITMSTVTICTIITAWRLRMATAWRLTTSSSNAS

NVQLQQVTLTKMLIIISCIFIICSLPRCTLSLARRIEPGFKLVYFNIYHAAHVIGYHGIA

AINSSVNFCVYYSRSSKYRTKVQIAFCRKIATNYSSNTGDSKSQ*

>TRINITY_DN352723_c0_g1_i1

MQSTDAMDNSTSDAQETFSNLLDRDVLGSQLALYMAVSLMAVIIIVGGVGNIMVILAVRT

TKKLQTVSNAFVVNLSVCDLVFVMVILPFNIYTYLTDGWHLPLFLCKIIGFLGYTLTGTT

IITITLIAWNRYKLVVDSQNYTHIFCRRNMAIMLALAWVMPMICLVPAVMEVWGRFGYVT

MMVTCNLLLDHDSQSFKLFLLIVRAVIPCFLILFYYISIYRTTKASHRRVRRSRLIMPTV

CRLDQMIQRKEMHLTKMMGTIFIVFALSYFPCTISSIVDWNTVLSKRFHMFCLITVYVGS

AINPLIYGLMNSQFRCAYYAILLCRCCCSMSHGRDEALIKCTNSSPTPSNKIYTKVNKRK

RSQLSHTTKQQTNHKKSTPAAVFKHLTCPPDLAESSSENQDCTPSTKSTKKILPMCWTLS

QDCDLPSPHSSPDLSRTVHRTETQKTCLGDCDPEQCKCRNPFIV*

>TRINITY_DN352897_c0_g1_i1

MTMLTIDTCLNTTVIPTNISSEDGLLDDGEDEQLRELAQQVEYYVMKRFIPTISGLGLVG

NFLSLLVLTKEKLHKVLTKMEISAHIGLIALAVSDFLLCLLVLLVTTLPLKDIYYPGEIL

VYFHLVSNGLITVFIITSTWLIVVMAAERYVAVCHPLRARNLILLSRTRAYVVVLFVLCP

LCTIPVFLESYIQEVVCVDGQVIYRVDTVSRQSMVLRRIVWAMAFDFIPCAALIYFNTCL

IWKIHKAKQLRERMAPLHTSSRKPSTSSNVQKFRWDGNDAKNLRAATEPDNEVNTGKKSF

VGKLHISRGARKQRDSNLLDLMEITESSPSSQPSYNGGVEDRRGEKQLLCQQGKNGLVKT

SSCTTPSSSKLSRAGTYHNRSLSTKRRISDNALHSVTATLVAVVLTFLILVSPWELLKFG

MQYSSTPMDKYKMDIAVYLTNLMQVLNFSFNFVLYCMVNKSFRHTLRTVICLQAFTHVVK

*

>TRINITY_DN353958_c0_g2_i1

MWQIVFAVGLLVAPAVSQMTLSKKTCDANRWHVSVDTTNANILLGGLFDIREPGTGGIGC

GLPSKELLQAYEAARWTIRQLNTNNYVNRIRFGMDAYDTCTLEGSALAAVQEFYPQSVGR

AGTSSTGNSYCTKSGSVYKLGLIGPMQSSVSKAVAEMAPRIPASVVSMRSSAPSLSNKNL

YPTFLRTDQPVSIFARAVSALFKQLQWEKIVIVYGDDDYGIGGYQEVLQAAYDAGICVSG

AISVPHVGSIAEYRAKLQNLASYGTTAAVFYGNPTDALTTLQALEGISGVNNIKWLFGQL

ELETDYSAYRAAQGAIVALATTTEISSFKTYYTNLNINNPPSENPWLQDWYMNKYQCRLT

GVNYAPFNSKSLCTNPRLQDFKQSPYVEQTILAIYAYAEAIKRVCSGSSGVCTSLRNMDP

LTFHKNYLSKVDVTLPNTFPITELRGRRLAFDVNGDPLVSAFTFYNYNNPGGSFTFQQFA

TYKDGNLQVTTNPSFPRVGSSNLPVASKCSVSGCTNCLQPTMGVTLAYKPGDFVIAALVN

AHESDQRGLTCGSVIPDLLIDAVAARYAAITDTSLASHGVSLGYLIVDVCPGMQAAQSFL

TNLLGGYKLYTDAKGVIITTTNIVAVLDMTREDTGWRMAPMLASMGIPQIRVAPTSSMMV

QNPKYPLLTSTVPSQETVQLSIVQMLSALQWKYVQIVSEASGSYYRAAENFRRLASHMDI

CVALWSSFDMDPVTMVSRMRDVQNAPVVVVFGTRMHLRSMLQAVKNVTASPTVLRMLTFV

SGSDEFGTSDNVVAGYTAESTGFLSVELADHVSVQMSAWLSGLTLSDIVNDAFLAQWFEW

KFSCALTSASRVRYNNVCTTPTSLVSTSVYLSYAPYIIKTVNAVASALKSILDVNCPGTT

GICAKFRNLNGVGQQIVNTLNAGGLATTDLSYFNYRNQVFTNVGRFDSSTRRITSLFPSN

IFTATGVTANTVNTQCVGHCPQCQYSYGYQKFTYVPGDWLIGATFSISNPGPEGQRPYVC

GDVRSTNGFQYSAAVMYALNRINSGMGPVKLNNVTLGGLIMDHCNNEGRAYGQVADIYSG

LTELTWRDMMGGKDSFSSDNILAWLTDNTASTKEAAKILQPIGVSIVSPSATSTSLMDNP

TFFRTIQGDSTTAMAIIKLCKTLGFTYIQVVYAANNYGRDGLNTMINTARQEGLCVINSF

ELVDEANASLAVDTVSAGSSKVIVLYMGTALTTAFLKEAAQRSTDQLTIISPESYSSVVA

NTPNKYNLRNLLSLQLQNPIISDFRNFLNNVNENSKNPFFARYYMKVFQCNLPGYYMYIR

DCSNQALTSSSAYVPSNFALSTINAVYAVAGALQTTLVEFCGADFTDKCAAFMQSDTMYN

RFVENLKSVQFQDPSNRLFRFFEREGNVVYDILRFDGNQYQTVGTYGSASLTLTDSAQLA

THYSSVESRCSSQCSECIFSGLSFSFTPGDIYLAGVFDVHERGQGVFDCGSMNAENGFQL

LEAFHFALQQVNSKQGRFANILPGVTLGGVGLDACQSAIRGGYLVSNINNGLTTLVRDGK

EISPEEIDVYIGSYSSDSSMYLARLLTDLQVPQISYASTSNSLENKLLYPYFFRTVPADD

KQVLGMLSYLDKNNLRYVQLLYTKDAYGEQGSAQFDLLIDQYNFSICVAQRVNFPEGPVV

SEESSDDVVTSLLQKPVANTVVIFAGTSHIRAFLQAVGRNPAAVGTFKFVGPMTWGDKYS

VTENIPDVANDAVTFVLDFQGGVGNFLESYLTKRNPANAGNNPWFEEWFQYTLNCYTSPS

NAGRYPRPCGTNTDLRTNVIEDNGVLHVMNAVYAAAYAVDATLKEKCGVNYVAVCEAYRS

SSDRRTDIRDNLEKVNFTDDTNTPFYFVNREGNKGYKLFKLTSSPLDTDMDTDVIYNRDP

IGSFSSDDVLTLNMAVVPKYDGSCKRKDACAECPTIRNRGVRYAVGGDPNGAVTLIGVFD

VHNQGTELYQCGSINMAGFQKFLTFFWTFSGIAANNNIRLLALDTCSNSLRVGQDLYSLL

KDGTLCNSEYFDLDGLGIGNVGAVVVTGKDNTVAASHVLEPLRVTYVSSDSVSPLMDDYQ

YLLRSITPWSGYVRILTKFLLDSGWTYVNSIYVSDGMGRTKYTVFQEISKENGICLRNGL

TLPEDMSDTDIRVTLSQLDGSSGANVVVLFSMPPLTVRILRLAQELGIADDFLWVFAMGE

SEMRMYFPAGLTFQALVLQPVIPSLDNFDDYVENLVYDSANDNRNRASIPRVWWEDFYQT

VHGCKLQNAVYPLDFDRTCSLNLQISDFNQSMMLHYTFLATAFSVRGLGFFYSDRCRAGV

SVQQCLTRLTSSRQLVRDDILNVMYTTTQATYQFNKPLRYIEGMYNMYLYNVRGGTLNKE

MYRSYRDGTFTAHNRLLDITSACQTPDGCGCKRKSATSQAQTDDEFSPTMPRNFYKYDST

NKQLYDWPIWAIAVAVLTCLGLLVTIIIFLYLLFFYPVKSGTSILGYLTMVGIMGVYAIN

FAFFLHASNATCGARRFVMGVVYTIVFAPLLVKAIDNWRFADLEYGNRPYRGLTSSLTLF

LIALGIILIQCIIPIEWLILVHPTAHRWTTNTMNDYWWCDPPDFYDIGLVCSFIFVMFII

LLTAIFAALAWDSESNNRESRWILVAAIATAGCFLVWMIVTTNAGPIYRDPTVAIANFVN

ATLLLIFIPIRKLHLLCVAKSEKPEIYGAGDFDPYSNVYNNQSYDPEGDDYAYEPTEKPD

DGFTAD*

>TRINITY_DN108432_c0_g1_i6

MYNITEEWSFSSTSNNNIGINGIGNTTNPNDDKLEGFLSDTTAYIIQTVVEVGLLLEVVL

AGVVMNAINMAVFLRQGLSDRINLCLFSLAISDTGYTLFLLTPRFQHLIKLIDPQLGQYL

RARTVSLIGVLAGFMITSNCLTLLISLERCLLVISPLKAKTMLSTRHMAVIIVITFCVCL

SCSFPYSLKYDLITVTDPTTNHIKYKPVYTPFYVNNKGVIEGFFLTFKIIVPTIVFVGVA

IATSVIAVRMRAVTAWREKSANTTTSSERKESSITKMLFVICVMYTICNVPAVISPFLAL

MIPGLDAGLRYQNTFFVSATLMQLMVGINSSFNMVVYYVMSTRFRDTLKELFTLCKTKNT

TIAKSFAKPSD*

>TRINITY_DN354343_c2_g5_i1

PVVFEESAEMGDSTKAMTGHTDVITPFPLATSSPDVGGMNPEEHQVMRLTWLMSLFIFFY

SVIFLLGVIGNALVVWVVTRNKAMQTITNIFITNLAVSDICMCLLAVPFTPMSFFFNSWI

FGKALCHIVPMTLCISVYVSTLTSTAIAVDRYFVIVYPFKPRMKVYMCVLMIVAIWIISI

SISLPLGIYHEKERIKNSTSFQCSEKWPKPQARQFFTVTSLVLQYIVPCTIITFCYSMVS

LALRKRSKVKIGSGARSREREEQEIRRKKRTNKMLIAMVVIFMCCWMPLNAVLLLREYEE

SVEHWYYFTVIFFTAHVIAMSSTIYNPFLYAWMNDNFKKEFKTILPCLFMGGRQSQNNSN

YTQYTSVEAQPSLMLNRSPMKNGNDNNCNANSDTATANNTANNAKETKAFYDADSEKVHL

NIAEEQGTEDT*

>TRINITY_DN354680_c0_g1_i2

MDNTTTEDGGSLDVRARQNVVLCILGLLFNSTVLAVIVVSVRLRKSAKYQLIVSLSLADV

LLGLVYLPLDTVMALDGGVWNYGCGLLFFTYGLQEFVIPTIAALTLSALCIEYSFTMFCN

MTTVTRRRVVILGVLLPWLLGLTSLLPVYVLQFVTSSGDFTPPVHCVKEEWHVSTMALLV

GLYGYLPFTVLLACLLLMVLAYCCRPPDDRIAVRTIISRSEGVYLTHEFADTFFAALLSL

GFNLPLLVELVLLVTCRDSWCHSTQRVRHGLEVLRTTQSVFFPAVWMLGMEFRHGLFTSC

NCCYKYCDMEVEQAFT*

>TRINITY_DN357826_c0_g1_i1

MATRNESDNAVMSEVKPDLFKWHGTEFIFVPLMMVTGTLGNALVLYITHFRWKSAIFSFF

IKILAWLDVTNLTLALPTLLAITVDSDMSAFLPLCRVTSFVALFTAIASSLVLVVIAANR

FRKLCQASRSQVKLPLAKKLTVVCVGLGTALSIPGVWVFGRHSASFKGDLGPVNISSCFI

ADDVLLPLLGTWAGVLGVIFSAVSMSLVLLYGFTIRALRQHDQRVINMGRRPSALSNNSN

KTVSKKHTWVFILVTVVFFVTYSPYLLTVVLLIAIPDIESTMGPVAKAFFDVAKCCPLLS

NVSNPIIYSFTSERFREECRKVFKLRPCMRMLTLKRKGSVTTSVEMSQSEET*

>TRINITY_DN110966_c0_g2_i1

MRSSVEDIIMTTSFTTVNENADIANRNISLQTFGQNSSSHDGCIQVRFSEMDYVPWNNSD

NIISSDVEILANRIKNNIQGFLFLIGGPANVINMLVFYKQGLQDRVNLCLFALSLADELC

LTMGMFVYGEQLHLQFTTKEKYSPMRTFIINTNLLSILGFPFHVSPVLSAIIASERCLCV

LSPLKFQTLLRTRTMAVIITSVNVLTFGLYFIVSFRYRVGCVYDQETGVVFKTLVVGEFY

KANKDLIDFLDGYVFALGLPSVMMIVVITTTIITIVKLRQVMTWRATTSSSISAREVALT

KMLIGNSFFFIACVSPTALIRFCWYVFPEMNSGGRNHNLFLASTWVVQVFACINATFNIF

VYYAMGSRYRETFWALFRRKMTTK*

>TRINITY_DN108934_c0_g1_i1

MSRTRDNTVNPDTALFNVSSSFHDVVHNMTSAGTTDANVTEMTTVPEGCVVFPFSTFSPW

DNPEDLVSYTTQQLVEEIVCVYILNPFFLVSFPLNIINMVAFWKHGLRERINLCLFVLSF

ADTIIMSVFLTCHLDRLYAAVTNTPYRLVITKFAFNHTIHSLAGFMYVSVFMSTLIALER

CLCVVSPLQAQSMLKTKTTAIIITIGHIVILGVNYVVAKKWEVLCMYDPFTGHSVDSIYS

GQFYLNNKQLIDILEGLMFGILVPGLSVIGVSLSTIVTVIKLRQMQEWRGKSASAPNQNI

SKDVTLTRMLIGTNILFVMCSTPPLIFHLAVPFVPDLNLSGKYYNTYNVLFNMQQFCTFV

NSSFNFFIYYRFGTKFRETVKDMFCGKHIKT*

>TRINITY_DN359032_c0_g1_i3

MSVRGPSAITNDITILTNPTTNTTPFSPSPCFSPALSPDSPISTDSHKNCTRNPESKLVN

LDPVFMVYYLGSLSLVMLVNAVGNILVLKTIYRHRCLWLPSNVFVCSLGISDLLFAPMFF

LYNLAGVKTSAILNVFGHWSVCRILLMEVLALEICSAYTLVAITITRYIGSEFALRYHHY

VTSRNCIAAAVVIWVTSQTSCAIVYAVQIPHAVSSGEYCKVCRYESIYTVRELGMFTVLQ

FGLPCTLMLAFYLKMAYRARKLNKRVSAEAFCSNVAGSEQSRATLNKRRHKSFVIMSLLI

GCFALSYVPMIVYLFIVIVEGQPEIHYEYFSATSRIFLLLNTAVNVFIYAGRMSDFRECL

RRDTKCFFCSLSCACPCSCSATSSLTSVSPVADVSGAVGMTSAPVCVIQGGGGLPAS*

>TRINITY_DN359917_c2_g1_i1

MASAEETVRILNEKKIACYHLMLKRERQVKAGNISYKGWCPVAWDDIVCWDVTEPATAVV

KPCPDYISQFKTHEFAVRKCLKDGTWEMHHNRTMNRSWTNYSACYDTHPVSNDIDMTYHA

EQADKLHLMYTVGYCVSLAALVIAVFIMLYCRRLQCKSNTMHINLFLAFIMRASLAFLKD

RLFVGHLGLPQDITYIDGRLAFVHEGLHWECRLITVLFMFAISATQTWILMEGLYLYMLI

HRTIVTERYGVRPYVFLGWVTPWVIVIPWVIVKFLYENTYCWNMQQSAGYFWILKGPWIA

MVMINFFFLLDIFRVLFMRVRSNHRHVGSSKYRKFAKFILVLIPLFGIMYIVIFVAFPSD

HVDGDGFNVIHLYIEMGYNSFQGFILALLFCFLNEDVHSEIRRMWRRHRSRREDSKYLNR

SLAGSTFRHVSRDNGSPQLSRVPRLIPSWKKTPLTNNSSSVRSHHTVSSLDGASSPESGS

SCKEKYPLGVKFSSTSLPNEHYRLVTSPSNGSVSSADRRRSSGIRESGSSGGRESGGGGG

DGGGGSSS

>TRINITY_DN360167_c0_g1_i1

MGEWSWEAAFHGSVHTATLAVATVLALFVLVLGSFGNGLVLVSAVRRRKKRTNFDLLILN

LSAADLFLCVVLAPLYLYTLFTDPPFPKGFCGSLLLGGTGFGLLSLLSIVAIAIHRQARV

VGRAKRSLTTRQTGIVLGVVWVLSGAGAVGATLHAMLDWRDSYDNCQVLIYSNDPLLHRF

TLFFLGPVVLISFIIIVVAYAVIARAVRTQSRTRTRGLAALYRSTPIYRNGLSNKQNDPL

VLEKPPGERRKKRLGGMKQCQCCSCQAAAERESKAVTMCLVVMVTLVLCWSPLLVSQFIE

LATGPSIILLQVKLCGIALVFLNSALDPYMYAQNQGHVKTRYGRFCWDVLRCQCTNPRRT

RFRTLRTRPLGPARSSLLEVNTPSLPAPDRGVEDRGNVEYAEDVRNEGRGVRYVFPGNEA

SPGNERWLRRQTGQGTCGPGTAPPWQSVFNPQTPCTTTTVATNHTAVSITAIRNCRHTCR

PKVVMRDVKTLVHKSCCHVHVPSDQSLVES*

>TRINITY_DN112109_c1_g1_i1

MDSPHWSGNASNTNSSLHDPIDHAGSNNVLEVFPVVTESPDGGWFGKIGTSVILIFGTFG

NVMTMIVLRKLRTGWSAMNVYLTALAISDTVMLYNGCFPMWARKVMNFDIYASHVIACKG

MIWLLNTAASLSAWLLVALTAQRAASVVWPHRINILCTRQKSVVISLVITFTFGLLYSHI

MYGYELVKLQNDTSVRCTFAFADYQDFWINVWVSVDMVMYCVLPCTFLIICNAVLGWKLM

ASVKKANEKFVSTMRDQRDTRKKRASSTTLTIFIVSTAFVLMTIPLMTYNTFFNTYASQD

EQSRQFHYILYEFFIVFSLCNFAWNFYLYCLTGTKFRREFLMIMFGCCGYSSSKAKSAPD

TERSQSSGAETVYKNTESEGIEDF*

>TRINITY_DN360803_c0_g2_i1

SVIVVIAIVSVLYNTPRWFEYRLLHLENPISNATCVGVDKTQLGENSVYNKIYFGWLYFL

VMCFIPLCSLAVLNTFLVLAVKKSQRQRKDMNVKQSRENNVTIMLVSVVMVFIVCQVPAL

IYNMAYAISMATVTTSPAWEVLSTVRNFLVNLNSAINFILYCALGQKFRRTFVRTFCPCI

ARRARDGFHSFTTANHDMRANSSSNNGAVYIKLMKATKGGKGGVSNVGGGKVKGGKGGLS

KQQNGNRNSMTDLTKVADQDSGVVVHKYSSPRASVDSSNCDSGLYLASSGRGKAKTRVSE

RSLLPKTRDDV*

>TRINITY_DN362443_c1_g1_i3

MTWWRLPWTCPWSVFVCLLWGAMCVHYAVCSKPLEDQEYDTGPTRGDLAGGSKTSGKPRC

ASGWIHLHRACYRFVARPPGGWSDAVSDCRKEGAQLLSISSTTEMNNIKRALKNKGSNRP

RTTWWIGLRKNNTDWQWLDGGDFSYRITRWKPGEPNNELGKEYCVEMDNKGQINDKDCTA

LRPFICEKLMTRDYSHWTSPPTLIPDTTTNATTTTTTSAVAATTTTTTVRPVTSAISGKA

ATLSIYEYTTTTTTLTISSTKNDSVTATDDVQAVTSMTLSPVTTTTASSFVLSSSTDGEE

NERTRTGDEKTDGQRNVTIDDDKSKGQGKRPDGKQKQNRGNNEDNDEEDLLELFAGCFMM

EGPSRGNRNRKSFLYTKGSTTCPEVVASGTRWPVTRHNVVQSESCRRGSGNMTFFCGGNQ

TCWRGEPNVDQCASPAFRNLLKRFSTDDDNDKREAPEPEETLAVTSELANVTKGDDVREE

DIVVTSKLIKGLVSSGAATKIKDRDDVKSIVANVVKAGSNLVSTRKTAVLWTRMSEREKI

RSATSLLVAMETATVAMAAELKEPTTIVTRDDNIELELRVVKVSPSGQDNEDMVYNAEDS

DTQFSIPLQTLGRFSSGGLAKMVFMTHYTMGNILDDNNSDGNRITATDDKAKDKVDDNKT

PATLASYIISAAAASSENSVNLPQPVTFTITHTKNIPEGFQPLCSFWSISESGLGVWSQE

GCRLVDTNSSHTKCECDHMTNFAVLMTVQDVEISDVHKSLLRMVTIVGCIISCLCLVASW

ITFTCFTSLQGERNSIHKNLVVCLFIAEMLFLTGIDQTQNRVACGIVAGLMHYFFLAAFL

WMMMEGVHIILMLVQVFDGSRSRLPYYYAGAYGPPILIIAISAGLYAEGYGTEEYCWLTT

EKSFIWSFAGPVAVILIVNSVILIYAMTMVCRHSDYVFSTKDKTTAGSVRSWIQGAMALE

VLLGLTWIIGFFFLTEQSVAMAYIFTILNSTQGLFIFIFHCLLNKKARKEYRRVMRVKRQ

PSSTGTGSGTHTASLAKNKLTSGPSFNRHSPCKEDNPPTYL*

>TRINITY_DN362613_c3_g2_i1

MTTNNSTTTLSNDNMGLSEAVGVEAYSVVLSTTVLVLAVVLGGGGCITILLAVFHSRSVR

KYSYTLLLVFFIFCCGLDLVWSPIELVHLLIYHHSLHHPSEHFAVVTFAIYVFLCAGVSL

VIMAFCMENVLRLTRCFCSPLRRVWPVVVCGITFITSIFMTVTFVYASLDDLHEATSTYL

ILHPQSCVVRLSILTVLLLVVVVAVMMMVVAG

>TRINITY_DN363875_c1_g3_i7

MGSQNPTYSAGGAPGLHQQDGDNYYTLSLSVVDLNGQDDAIKMTSSSSWDDAVTEEVMRF

TLETFVTMFTTIVCVVGNSLILVVAALTPAFNNFNRCYLFSLTTADLFLGLFVTPFCIFN

TMYGKWIFNSSIFCCVEAYLMALFVVVSMISMAFIIVDHYVAVRKPDRYHVMMSFNRSLC

WIVLTWVIATSFCCPPLFSLQRAFYYNETFLCVIDRQNQQTYFLTVMFMVTLPTATAVII

TSRYLFTHAFQDQKRFYQRVYEYFSERSWNYYISFVITVMFFITWLPFTIIRVAQSVQGW

SREDIPKEIHFYCMWFGLSNCFTKFIVYLLMSPQFRQGLREVCDLQGGHRGYALCGRGCT

SESSTSAPTESYGPEVITVPTTRK*

>TRINITY_DN364950_c0_g1_i1

MADRNDSEAGTETRPDVFKWHGTSLIFIPLMMAAGIVGNFLVLYITHFRWKNSIFSFFIK

VLAWLDVSNLVVAMPLLLIITLNDHTPHFMAFCHSTTFVALFTAMTSAVVLVVIAANRFR

KLCQPQRSQVTLQLATKLTIGCFLLGAVIATPSVWLFSKDTVSYQADVSPVNISYCFIKS

DASPPLMIAWAVVMGGTFMSITVSLVVLYGFTILALRAHDKRIATMMRRPSAVSNNSKKI

SKKHTLVFIAVTVVFFLTYFPYFITVILLLADRTIESHMGPVAKAFFDLAKLFPLLSNMS

NPIIYSFTSERFRSECQKVFKLRPCKKLLNLKRSDSTTATHSHEMSQSEES*

>TRINITY_DN365822_c0_g3_i2

NQQQQEQQGNGKRKKKKKIRSENSSISGETRSLNNTASQKKASKERRDFNFLANIRTAGM

LFVVSIVFIISFLPAWLMAVRVIAYHIVVFYLYFVYNVANPVIYAFMNHAFRKELKRMLQ

RGLNYCHD*

>TRINITY_DN366636_c1_g2_i1

MGYEIFVTHLSAADFLMGVYLAIIGIADRIYYGDYRWNDAAWKQSVACKVAGFLSLVSSE

VSAYFICLITLERVLVIGFPHKNLRFSQWSAHGACVVVWLIGLTLALIPLLPITSHWQFY

SQSGICIPLPITRKQFGGRNYAFAVMIVINFVLFTLIAIGQLLIYWSIRASTVRSKGSKR

KSVDMAIARRLLTVVITDFMCWFPIGVLGLMARNGVPISGELNVIMAIFVLPFNSALNPF

IYTINIVIERRRKVHRDQKSSKDNREPGSTQVSSRALDVDTESKVGKSTYSKEGVLKLVD

TCLDQGVLTQNELRRHVWNIYEG*

>TRINITY_DN112915_c1_g1_i1

NILAILLLIGGPANVINMVVFYTQGLKDRVNLCLFALSLSDLLYLITATSLYSEHIVMQF

SSAERYGPVTEFIINNNLMGFFGFQWVSQVLTAIIASERCFCVLCPLKFQTMFRTRTMAI

LIITVYIVVVGLFFVVSFVYRVACVYNPFSGSVTKTFVTGELYETHKVMVSYLNGLVFGG

VLPGVVLIVVTTTTIITTIKLRQIVTWRSESSTSLSAREVALTKMLVANSIFFTACVVPD

AIFRIACFCLPEMSMGRRNQNLYLSFVWSLDIITYINSTFNMFLFYAMGTRFRETFWSLF

KRKSQPGKGKHTVAPTKSSTSSQQRVKTLCNT*

>TRINITY_DN366890_c0_g1_i1

MSTGVLLHTLTTTLTSTLTSTLAPDNEVDTLADGFALSTQPDAGMTKTTLLTPVARPPGV

RPAAPDYLLHTQFVCDRVLIPLVVSFGIVGNLLSLVVLTRKEMASPTNCFLTALSISDIS

LLLLQIPMFFGLNAQVASTDSYRLFVRYYTVILYVMTNVFLTCTCWLTVAVTVERFLSLR

FMMHPRLICSMVRAKRVILAIFFASFLFHFSKFFEYMPNT

>TRINITY_DN367418_c0_g1_i1

MTNSNAATDKSVVLTPSVTSLTSSANTGDEMTTSWLDTRDIDVVLFLHQKNMDRMSSLIP

FVIYLVVLMVCGVVGNSIVFLVYYSRFKSSNPRTFVLSMSLCDLLTNVWALLTEMLEIRF

SYTFYSAWACKLFKTVMNVLVLFSGLVLVAVAVERQKMVCGQYARAQSVDRIVRIALITC

CVLSVGVSLPRFALYGRKTFLFAGTNITGVQCNIQDSHKYSLFPVIYNGLLALVFVLTVL

TMIGCYGRIGVHLCRHKKEMVKTSRKEITSTSSDTRPHQVSRKKSETDTSDTDTNTSVHQ

KDVRVSQDPHNIQMVQIFNQTSTAPQSLSQKPHSDRETLRQRGESAGEEAKKPTKAKLSI

TSTEGMKITPLTRTLSRQTSTSSGKPIPSRTTLMLFILTVCFIINFLPYLILEAMLSLAP

TSPVWDTDINLLFLGFRSYYLNSAINPLVYSFCSVKFRYECLQLARGLRQRWARITAITV

*

>TRINITY_DN367471_c0_g1_i2

MTTSTGAADDTTTMNSSLEDYYDYYNYDYNESAIYSLPLDEVIPVTMVYSLTLLLGLVGN

TLVIFSISYYRRMRTVTNVFLLSLASADLLLVLICVPIKAVAFFSFTWDFGEFMCKMVNY

VQTFAMICSVLTLTVISIERMVAVVFPLRAKYLCTMRHAQLVVLGVWVVSAVLATPTVFI

FLHMKVGVVRTAYWCVKNELLGEGLWLKVFETYMMTVLFIFPLFIMIFAYIVVGIKVWDV

SEIRTGGKVISTNDPNRRRSLTKPESGERSLLGNGSSQNARGSRQISDERETRKQVVGML

VMVVLLFTLCWAPILTNNLLTAWGHLHVLHYGYLKPMRQAFFLLSYLNSCLNPIVYAFFS

RNFRQSFKMAICACVKGKAFVRAYRYSISAASTRRSLAHTNGRTLTSVYETGTGSSENDM

TRSPTSYSPESLELQKI*

>TRINITY_DN78691_c0_g1_i1

MSDNTFASSNEEQGVVVTTIATPAQAANHHQGSGNSEGLKSLIPPGCLLLEQGDFVPWDN

PDNILSRETDYWVSTILIGTIVLPMLFVLGVAGNVLSAAVFKRQGLKDRINLCLFTLALA

DVLVLVSNFFLTAEKVYRDLVGFSGFFITYFPGITGVTWVSQFLSAVIASERCFCVVSPF

HAQKFLKTSTMAIIIFTSSAVLLGGMLAIAGPKHTAVCMFDPITNTTKDIVYVTDYYLNN

KEILDLFDVFLYATTFPALFLLVVVMTTAVTAVKLREVMAWRQQTATATSTADDKDNKIS

AKEVALTKMLIATSVLFIVCLVPILLVQIATFLVKDLNYDGRYHNLTSVLWQCINTFRCL

NSSLNFFSISTWDPSSGRHCGSCLALVSQRSDQVHCQQNYVPKHLAVYFSPKEKPFLS*

>TRINITY_DN111878_c2_g1_i2

MTESPLEIDTLDLSNASRRLARDNGSETKPSDVFLPWRNPQNIISYEAFLLILKVVQLYI

FPFIFVVGVPTNVLNCVVFYRQGLRDRMNLCLFWLAIVDMCFLITLTSDIATSFVALVDP

WLAKKYQAFAIHYMAGIFTGFAINSGCVTAVIAIERCVCVVFPLKAQSLLTTRTMAIILT

TITVLLQLFFTTWILELEPRQVSDTNEKPTWSLKTAQRANQEMFNLLYSIFFDSIAFVLP

IITMSIVVICTIITAWRLRMASAWRLTTSTSHSSNIQLQQVALTKMLIFISCIFVICSLP

PCILSLIRRTVFEFRADG

>TRINITY_DN111878_c2_g2_i4

QRQISDYNTDQAQIQDIVTQSLMENTVSDINNSPHASFQNNQSSTESTGVFLPWRHSRNI

ISYETSMVIEEVVRLYIFPCIFVFGFPSNILNVVIFYRQGLKDRMNLCLFWLSMVDLCYL

TLIIPWVVGTFLVRTDLWSSNEYVVKAQYYTIGICAAFLQTSGCVTAVIAIERCVCVVFP

LKAQSLLSTRTMAFILLTITIFLQLSFSVFVLQSELTQIFDKNTGKTTWSFGPSQWERQE

IIFILYDVFFVLVQFTLPLITMSTVTICTIITAWRLRMATAWRLTTSSSNASNVQLQQVT

LTKMLIIISCIFVVCSLPGCALSMLRRIDVSFTYGYSNHNISVALHITNYTIFSGTNSSV

NFCVYYLRSSRYKSEVHKIFRHKTDSKKKKTKTNKKNPSKWTGTKP*

>TRINITY_DN111878_c2_g2_i1

QRQISDYNTDQAQIQDIVTQSLMENNVSDINNSPHASFQNNQSSTENTGVFLPWRHSRNI

ISYETSMVIEEVVRLYIFPCIFVFGFPSNILNVVIFYRQGLKDRMNLCLFWLSMVDLCYL

TLIIPWVVGTFLVRTDLWSSNEYVVKAQYYTIGICAAFLQTSGCVTAVIAIERCVCVVFP

LKAQSLLSTRTMAFILLTITIFLQLSFSVFVLQSELTQIFDKNTGKTTWSFGPSQWERQE

IIFILYDVFFVLVQFTLPLITMSTVTICTIITAWRLRMATAWRLTTSSSNASNVQLQQVT

LTKMLIIISCIFIICSLPRCTLSLARRIEPGFKLVYFNIYHAAHVIGYHGIAAINSSVNF

CVYYSRSSKYRTKVQIAFCRKIATNYSSNTGDSKSQ*

>TRINITY_DN368154_c0_g1_i4

MTVSVTFLNSTPTFPGSSGPCGYLIAIMTSFYICTFFLTCGYALEAYIRLRQRLQSYVSM

DVNRNTGVSSICMWAVYIFSWFLPITLGVVLMFLSRLHKNTSGSMDRILPLECSLCFPIF

STHQSFCWMYVEDGLQWLLVYRLVFLIPLIFVFALNMGLYLCIARNFRQVSMRRGLLSYH

QRQEESTLKKKSAMYQAAFIVCWIPTLVLTCISFSDSYVMADYYPVLVLQAILGPLQGLL

NSIIYGWKRNSFRRALTESSHLLSTNRGGTMSFTL*

>TRINITY_DN368733_c1_g1_i1

MNGTNNQSNSDLPAYELGMALRKVYIWVLVAVGLPGNLACLLTILFMHMTTATIYVALLA

ATDSLALLLKLVFHQLITHRKLTAVSCYFLYVPSFFSCYCNWILALICFERFFAVCFPLK

KQAYFSKRKVYISAAVLTLFLVIAFAPTFFLYEDVGPERKECILRGNMVVFKEQVWSAGL

LGSLYFFIPFLLVAVFTTLIIIGLQRHRRARQAMMRTTRSGGGG

>TRINITY_DN113387_c1_g2_i1

MKTLMKRDLKMNYSQVQVSFSSSLLGLSTQYTHGSHTNSVKENGGIGSTVFLIYLNHLIW

QIAPPVLIVCGTFGNVMIIVVMRVLRNSKSIACLSYYFTALAVSDMCLLLVSVIWYWVEM

VFLWPPSAFRYNLLCTIPNLLWYTCSLTSAWFLVAMTYQRVTSVVVPHRVGVLCTVRRGK

LVIFTIVIIACVANVHFLFTYAYAPEYESCYPVEHYALVAYFFIWVDLFLASVFPFVFLL

IGNCILITHVIKAIRLSHKMRGNSDQQETSGASKVSSMTTTLILTSTAFLLLTLPTCTFD

AYRETLEFRIPDEHFNAQMDLMETFTLLFWFSNSTVNFFLYVLSGNKFRQETKRILCFCS

QTQETSNVIISK*

>TRINITY_DN370023_c1_g1_i1

MDRTMNSDFGTTDSNFTTASNVTSSFDAAHVTTDDPGRMTSNSIVTTTKSDWDYVSDSAN

VSLTVDVQEFLLQKDDEFAVFVSPAITFVGVLMIVGISGNLLVLYVYSQKLVTSGTFRLF

VQAIATYDLLDCLLAMPGEIFDMRRNYTFGTSPFCKVFRAVSAFCSLASGVTLVMVAVDR

YRLVCTPLGKQITPKIAKILILTCTVVAAVSAAPAVVLNGSQTTLTDNPAITGCDCSTAD

AFTGTIYPLLFSGFHFLVFLSVASTLIALYSLIGRQICRQERRRKSSFSTAGKGKGVGGG

KHVYVQMKENPCKGVTENSCNNTSESSVSMPYTIAITISDAAPSGSDDAPATAAAQFEEK

SKNSSQKNDEDSVESSGLGHDQSPCTHTLSKPEQNHSPQEQIQSSGEQQNQNQPSPKKVN

SSPQKEQASQTQSSSQRGKSRRPSKMTASVETRRLTWMLFVVTLVFILSFLPFLSLMIVK

ILEEGSPVSRGGWDLAWQNIVLRSYFINSMANPIIYSFCSRVFRTECVNLLHCRFR*

>TRINITY_DN370763_c0_g1_i1

MNNYVMTTTTPALTSLTLTPNMTSTLNPATMMTNASGSSSTLPTLTRAEQEQIAYYLSTM

KTIKESYIWVIVAFGFPGNLLSLLVICRMRSLGSPALYVGTLAVVDNLAILVKFLLMQFG

QHKVSLGLVGCKLMFFLGNHLVVYANWVLVAMAIERFFAVWQPLRVARTWTSHKAAICLS

VLLAVTLTITSPLFLIVTVRKGGSGQECVGDPQYAYLRSIWQWGQVTAYGFLPCILLFTL

NIAIIVLIGRARRLQQSLTSPSLKLHHTNSKNPSRTSQKNPSANTTTNKPFSHTSSKKGN

NNNRHATCTGGVQRQATILLLVTSTVLVLTTTPICVYLLVQQSWRPPVGSVAYAQKRLVA

FALRVLCDANHAVNFYLYFISARKFRAYFCKALCWLCMRKMDRRGSDVITRHFSTSRSTL

RVKDRNQGQSMALQRKEERNGDGEAGEENSVPLKLYTEEEQPSNANALSPCSSLL*

>TRINITY_DN371218_c0_g1_i1

METEGVLNHSYLFCMDTELGWGNRSYFTFFSQFNRGTSASLWAAIESAILIILFLLSTIA

NVLIFYTLSSNRRLRTVTNYFVCNLTLADVCFTLCCPLVAVIRVTGNWVLGSFACKTIVY

LSCVCAFVTIWTLTLISLDRYSCIVRSSKHRITPPVALILIAVVWVVTLAAFTPLLLYFV

VKECPYGEGTVSICTLGWPRTPGVSVPVLFTAVVCGVGFIVPIIILIVSHVCIFVKVHKV

RAAMRKQRQTRISTSSALLTPTIIPSTPPPFIHPPDAHLPSPAAAAAGGGSRFILPSLPP

KSPRLKTLALPNLSPTNSSMLPTPCTSTLEARKRRSSRDLQVVKTLVFLVLLFLVMWAPI

FVVFVLIMLDAYYDVMHVSSQAFLGAMCVAYCNAVLNPLAYGLSTDRLRYYLHTLLYSCA

PARCCCGSSGLSAGVRFSHGMGGGGGAGGGSTGGGGGGGRAARGVGGRLGEDGNTMVKSS

GGTSVS*

>TRINITY_DN113091_c1_g1_i4

MENVSSNSTEIDGISTVTVPPVPHKAQPEGLLSDATAHIIQTMVEVGILPGLVLAGVTMN

IMNMAVFLRQGLSDRINLCLFSLAIADTGYILLLMIRRLHSIIRLIDPLFGQYWKFLMTS

LTPIYAGFMTTSNCLTLLISLERCLLVISPLKARTMLPTRHMAVIIVMTFSICLLCLAVY

CPKFEVIKITDPATSVIAYKAVYSQFFKDNKDAIEYLFQIFTVIVPTIACIGVIVATCII

AVRMHSMAIWREESAHITTSLDRKEASVTRMLFAICVMYTVCITPSVIRPVVALLVPDLQ

PGKRYQNTFFTFMSFVHLTAVINSCFNFVIYFVMSSRFRDTFKEMFNRLKSHACC*

>TRINITY_DN114152_c6_g5_i1

PEGCTVQRLNKLEYLPWDNPDNIVSEEAETIVRRINENIIAILFLIGVPANIISMAVFYK

QGLKDRVNLCLFALSLADGFYLTFVMIINGEQLHMQFTNPDTYGPIRTFATNHNLTAFFG

FMWISQVLSAIIASERCLCVLSPLKFQTLLQTKTMAVIIVVVYVLVGGFFFVVVFRYRIG

CVYDLVSDAVSYEVVIGEFYKTHKDFITYLESFVYGVGIPGVAITVVIITTIITAVKLHQ

IVTWRAGTSSSISAREVALTKMLIGNSCLFIACATPIGLFRVVCLFLPDMNAGGRNQNFY

LIGLWVLEMLSYTNATFNIFVYYTMGSRYRETFWALCHRRRKSDKVNRY*

>TRINITY_DN113676_c0_g1_i1

LILGIPGNIICCIVFLKQGLSDRINLLLFWLAVADFINLVMLYMIEVRCYIQTWDKVRAV

NWVTWMNSKFIYIGLWANFVSGALIVIMSVDRCLSVVIPFKAGRLLTYRSMLVAIVVTYL

TLLLFYLPIILVYTVNWRVDPSTNRSIAYIDVIDWFPDSSLPLQLMSIVSTVCQPVFVVV

VIFCCALTIIKLRKASNKRLQMQESAKQDVGGVDNRITKMLLFLCIIFVILFLPQFGALI

TYSFVPDFLLYRKYHNTFLTVYGVVITASCINSSANFFCISGAQFQVPNNS*

>TRINITY_DN374275_c0_g1_i1

MSVTVGSSVFPSNMDHVPSVPALERWPNQSQEDQDHTHINPRYPNGIIVAILMVVGMVGN

SLVLYVYHFKMERTVFTTFVTFLAALDLTTTLTSMPLDVVIKTMMMTKTDKMEAVCKVAH

FEVYTSSLASGSILLLIAAARYSKVCKPMQPGWSVRRARVWCVLIVLVAMALCTVALIIN

GMEKVRISLGDAQSYHRHWPQNFTQKTNQTTLKENNVLSMSDTAAGDQHMHKPTQRNDVQ

PSVSEQVTKPNIVEVYICRASEKQKGTGLYYALYALLMTAYVTICLALLVLHCRISCTVV

QFKRQRSRQGSVLSDQDHLNLDTITSSMFRIFFTITVIFLLSYLPHLVCIIVQSFMFKPE

EAMPRMSRVLLDLAYNCPYINVVSNPFIYGYMSRRFRSHCFHLLCRCCEKD*

>TRINITY_DN374725_c0_g2_i1

MSYTNYSQGSGSVTMAAPTLNGTNGTSGEDEDTEFYRWNFVFSITLAAILSIAAFLAVVG

NAMVLTVVIRHRGMRTRTNLFLVNLAVADFLVGLGVMPFTVTTLIEGRWIFGKGIFCNIN

GWMNAFCLIASIHTLMYIGIHKHYSITKPLANHFKLRQIVGMMAASWVWAAVCAFINVAG

FTVRYKPGTTHCGPEYPSSTKTYVFHGIIQVSCIFIPVIVLTYCYTGMFRAIRAHSKRLQ

QNSTLEQDIILAQQKKVTITLFIVLAAFVICALPFNLYTTYTTIQKTKHFSPYLNPVAYA

FLYLNSALNPIIYAFRSPSFREGYKEILCQTPTYVISDGPMGTLKCRTVRSDSAYSRSVS

RCRASSSQSSC*

>TRINITY_DN374725_c0_g4_i5

MNGNYSHGFTTMSGHNITSNVSSEKYWENYYRWNFAFSVIVATVESISAFLAVVGNSMVL

TVVIRHRGMRTRTNLFLVNLAVADLLVGAAVVPFAITTLIEGRWVFGSTRGDFCTINGWL

NCFCLVASIHTLMYISVHKHYSITQPLASHFKLRQILGMMAAAWVWAAVSSTITVTGLSS

VRYKEGTSQCGPEYPMGLKSYIFHGIIQFTNIFVPVVILTYCYTRMFREIRAHTKRLQLN

STLEQDTILAQQKKVTITLFIVLAVFVICALPYHLYATYVTMQKDKHFSPYLNPLAYCSL

YLNSALNPIIYAFRSPSFREGYKEILCQTPTYVISDDLATDELQSPLRRRFSSFFSSMRR

ASISSS

>TRINITY_DN112442_c0_g4_i1

MVTMTNVSIGYMTTVIEKSHPFIPWDNPDNIISVETEETVQKVVICGGSQVVMILGIPGN

VVNMVVFTRQGLNDRVTFMLFFLAITDLLTLLSYFSISSECFIKPIDPVLAWTVETVQNT

NVIFLHIMTALLSVTLVTILSVDRCLAITLPLKVARILTYKRTVLTFVVVSAFTIGICLP

SFWTYTVSWVHDPISNQSVARAVPTQFALQHRGYISLFRTYLIASKMICLVAVVISCSIT

VRTLRAASKFRSEMAETKHRQPDVQITFMLLYVCTVMVLCLIPDQAFTVMTLAVHDFYMF

RKYHNIFLASYYFIALAQTINSSFNFFLYVLLSSKFRVTLKELLPCRQK*

>TRINITY_DN375472_c0_g1_i1

MATKPTFTTPFTSTINVTSSNYNMTTALTRTWKTYFSFWDTLYRIVQVVSTLETILILSS

NLALLLIILTSSSMRQKMRYHLVLSMVCANFIVGIFSAPFAVDYTIHRNWIHGCYFNVVR

TLLTLFVQNFVSMWGVVLILLEYIARLLRYQGPEWLMRLPHWLRKAVPGLLVASPWVLAF

LIQVPLMFIGLHQYVWVFWNETSCPIILQKWATYLLTITCYFAPALILIILVIVLLVLTR

GNAVTDRFRRAEMGTTLVSAEKEEAEGVGVHVVVAVLTVLFMGPEHVFYGARLHLKASWR

ASVIGGVVSMLFSELLPFILVVAWLLMLPEVRGRVVELATRLPCCSEWCRRHMTPPASSG

ATSIAPVAFRDLHDEHE*

>TRINITY_DN375633_c0_g2_i1

MKNHRIHVLPHGKYWWSLILVLMMILTVVVDSCVPSCQCTMVGTKKKQKGRRVDCSKHPA

PFTSLAHITFPPDTVQLDLAQNQLTVLKDGSFIQLSMLRKLNLSNNEISIIEPGAFEGLQ

NLRHLDLSNNKIGTINSSMFTGLPRLEKLWLNKNHINTIPDGTFNDLISLRRIDFGSDYL

RCDCHLRWVVQWAESKKVRLARETVCALPSPMKGRMLRKLTADQLHCEHDLQLPVFEIKP

SHSQLVFEGDKLPFECRASVIHPRTYIAWVREGGDIVITTNKTAGVFVHTSYSPDHTIVS

HSLVVENLDKSHEGIWSCQVTTPQGQVTKHVEVQILNYIGLSCPPTTRVTPKGRYVFEEM

LAGVKAKQPCQVGGPGKHVTYLCDRHATWKNLNTSACAFTSDLTRSLEKLAQEELNVTEV

GSYMQRLQQVAMTSKHRLISDVMLTDVHYLTSILQHQLLPLAFSSEYVSENVLWLTSNLT

ALPAPLLNQAQTDSQSTKRVLDVLQNLTANADVESSSQDWSQSSRNIAIIVLREVQLKAS

RVHCSLRRSRRQGLPWLSSRQLSCSRLGNSSENRHLWSENTDVSVDLPATLLQDAGILDE

NNTSVFITVFRNGRLFPTTTSQHVDAKLAHGNWTVASSVISVSVGHSVPNLTHPVVLVLR

TGNRRRPIAAAYWDFSANDGFGDWRTDGCEIIERKGNHTLVHAYHLSYFAIIEDVSEKPS

VIVMEPVIYVGSCICILCMLLVFITYISCFRIILCPKKMKHSVINLCLAVMLMLLGFVVG

INRTDMHIPCQVTGICLHYFTLAATFWITITANNMYKKFTKADRPPSPPPEPVSMPLPPK

PILRFYFLGWGVPIIICGITAAVNLNHYLGLEYCFLAWEPSLGAFCAPMGLLIILNLIFF

MRISCVVHTQSATALNEPDETEEVHTNEIELVPSQADTNLDVQSVTHDMESHRRSRNVSV

NGGGGVRRSGVGGSIRAGGDDEDEEDDSRSTASLPDAERRPITQLRALVATLFLYIVMWV

CGALAIARPFHAIIPYQELIFSYLYGLVSAIFGIFMVTYFCFTRNDSCVRWKSLFGFGPP

ATYTGPVATEQPAQTSEPSTPTTQPNGTVVKSNSNMDVSVYSQKSSNITKACNMKNNVNK

QTNNSNINLVSLPASSLTEVSGGSAQESIPNFYNPRQNGAARKFWQKNRQQKVMNKDVNK

DINASLTDNMSGTEHSQHLSQGTGSDANTHLSIEIQIQPKDRGSGKNLSSGGYDNASSSS

NPNLNRHNNTHGPPPTYCQVNNTTTAALITAHAQAHSPLGYKSDGGVYGCGGHCPSPSRS

HVSPAGSQHLVMSQQPQLPQHQRTASSCSLGAHPSAFTPVQPRNNNTLPRQSKPDSTPPP

TNMLSGPPSEGVFTAENCGVFVDGPGGVPDQGLGQYVTQASPYPGYEPCMHTNYLPPQLC

NIPVSHAYPHFGPPHALARSISPVVSGMQGVMYFPHPHGMHSPAHSHQPPQPLSGDSSDA

SSRHRRHSRYSNHSSNLHRATSGGINSVGAGIGLGGDGSKSPVVSDITATPSSHDGRVGT

QDSDSQSPAKKARSIDSDHHSDPTHRKKHRSRDKYGRRHGQGGMTKQRSLGWEEQFKDRP

AKVAYAYVNHNYRDKVMTKLIKQASESDELAKQAFWLPRSLSEYDRLTQMGFCNLVEDSS

SSSDEEDSFDNVWLPQTNNNSDLFKKETSV*

>TRINITY_DN113676_c0_g7_i3

VVIFFISKLSCYIQIWDTVQARNWVTIVNSKPVYIGLWTTFVSGGLIVIMSVDRCLSVVM

PLKAGKLLSYRPMLAAIIVTYLGFLILHLPVLFVYTVGWRVDPSTNRSIAYITVTDWLAF

DRELALEIMNTVGTIFKPVFLVVVTLCCSITIAKLRQASRKRLLMQESGKQQSARGDNRI

TKMLLFLCVVYVVLILPDVGAQITYSLIPEFYVYQKYHNTFLVVYAVILTGSSLNSAVNF

FAYLVLSSKFQTTLRELCHC*

>TRINITY_DN99081_c0_g1_i1

MMRAQWILSALTVLMLAQFAISVVPGQCPSLQNVCFYDDVAKLRPATNTQYFAVGGLFDI

RKKGTDSFTCGVNFSPMGILHTQAFLWSLNYWQTESLSVSIGAVVFDSCESKAQLIQNLL

GFEQCNIRVSGISRHNVVGFVGPTSSMEAMAAAKLTGDMDLTLISPAADSSLLSNGEKYP

YFLRTTPTIQVDIDIMAYMLADVRTKYVAAIYQESRMAAFDSFRDKMEDKGICITGSYSL

SDEETTAALGNFVRTTLASLEPTRFIALFLSPELLQSFMFQVTAVDVVKSMNLVFLMTSD

IRRNSPVFNGQNAIAASDAIVLERAEPTSVATAVNEFKKYWNTNTIRANLAQMGRISDPF

LIKFLNLGNSLPEFDVDAAYTIMATRAIVNGVKQAGRDSVCGPNTNLCSQFFDSENRGST

IYQKIRSTTNFFTPRDAGGAFPNGDLRTDAISYNIYRYRSNGTFEKIGEYSGGATPNIIK

PIQGTRAYLPGQCQDTPCLQCGELPSTTTITTTPAPTTPQNCKPDSRNSNFSLVYYPPNQ

EITGAYMRKTRGDSEFATRFDIGQRWVMALGVLAGLGILAVIIFEIYILYKLLGTRMGHK

WRTMWLGQLLLFGLLLCYLTLFAYIPIPTKVTCGITRFGVGVSYSVCFAVMLVKLMVILT

SKTTDSSLLPGDAESPNYLKGIYQFLMFVFVVGVQVVIDAQWLITVPPEAVKVISNNGDE

VWVCNHYTFRASGSAGTMTDMATFVRNEFENHILSLVYIMFLILITTLLALNAHGIITNH

RESVFIGIAAGFSISVWLAWTLVGGLNRDHAYAHEFGDACIAFGLFLTATLILFAMFLPK

VRQLVSMGVEGIYYEDDRDTMYGGSVIMAPSYKSRPNSVIYVNSQGIYSEPVVLGNGDGG

YVRQPSVDKPTPASTYSAPPTYLKKGSDSTRGGSVLRVTDDLSGRRPLEKKRATSEVAYG

TGSRPKSQRGTLRRSRSQTSLGAL*

>TRINITY_DN376500_c0_g1_i1

GTSQGRDICLCDHLTNFAVLLDFYGDTRPLDEANTTALNVITIIGLSLSILGLAMTVITF

IFFKKLRQGRAQQTLFNMSVALLCSEVVLLVGLKQTTNYGVCLAVAILLHYFILASFLWM

LVEAVLQYLTFVKVLGTYISKYTLKTVLPAWTIPLIPVIAVLATDYRQYRGRRDYCWLSL

DAFYYFFALPVGVIILFNLVTFVVIMISLLRRPEGLRSTRNKSNTAETNIKAAFTIFILL

GLTWTFGYLAIADARLPFQYMFTIFSSLQGFLIFVLLVARRRQFREQWRALCCGLGGQVR

GQKVTPTLSNSASSQSSTSSFTSSSRSRTSSGGSSKTLDILIKRADSNASRTKVLPQLSS

KSSTTSSKPRQ*

>TRINITY_DN376601_c5_g1_i2

MRRASIRILLGVIFLTLRTTTALHLQGTWNSKDFFVFLAKFGFQKTNPQKLEDTQGYIYG

NITAVDSSGAAVHREDLTLTVVDSEYFIHFYGNRTLPLQEVCPVMFQKIDTIAFDYKCNT

KGVEDFLRAIPCPDGRLCSDEDNPSNVLPGFQFTFKVRDTEQPRFWYLSIVSCSRDRSDN

QCQWQPKAMKEHITIQYDIWIVNGNPSSKGLNPFEHQFSFEFHDVFEIHMVAFLLISLIL

ILWMYAFLKQKHMITKLLTVVFAGELLSTSLGLTHVAVFAVNGQGVDWMSKVGTLVDIVV

QCLFMMFLLLLAKGWAITTDELKWKSVLFGICGVYTVLNLFLYVWNLVEIDTIISNTVEW

QTWPGYATLGCRLIAMVWFVVELRRTYHHTQHDNVNFIQHFGAFFLMWFIYLPVLALIST

QVSPLWRYKTILSISYAVDIFAYAVLLHLFWPSRSVLFMVNGEWPLPMYDLEITGLLEDM

QETTLFTRNSKESHTDLGKEEGGKPQSNGDTITNKLFSVNGSPHCPSSSSNGHVNTTYKD

ELSSDEDNT*

>TRINITY_DN376711_c4_g1_i1

PYRPGQCQDSPCLQCGELPTTTTTTTQSTTTDAQPCKPENAGNFSVVYYPPDQEITGAYM

HSKRMDSEFATRFEVGQRWIIAIGVLAGLGILAVIIFEIYILYKLLGTRMGQKWRTMWLG

QLLLFGILLCYLTLFAYLPIPTKATCGITRFGVGVSYSICFAVLLVKLMVILTSKSSENS

LLPGDMESPNYLKGIYQFLMFIFVVGVQVVIDTQWLITVPPEAVKVTINNGKEAWICNHY

TFRASGSMSAMTDMSSFVRNEFENHVLSLVYIMFLILITTFLSLSAHGIITNHRESVFIG

LAAGFSIPVWLAWTLVGGLNKDQDYAHEFGDACISFGLFLTATLILFAMFLPKVRQLVSM

GVEGIYFEDDRDTVYAGSVIMAPPSYKSRPNSVIYVNNQGIYSEPIVVGNGEASNHLRHP

GSGNPTPASTYSAPPTYLKRGGDSTIRGGSVLRVTDDLSGRRPLERKRPMSEVAYGTAGR

PRSTRGTLPRSRSHTSLGAL*

>TRINITY_DN376806_c0_g3_i1

MAVNSTAASLLTVTEAAEAEVSKLWPAILYTSLLMLMGSVGNSIVCYVYGFRWQSTVTKI

FIFSLAALDLFNSLVCMPTEIAMLVRIVTFNAPWWCKISRFLTYTLNGSSSLILVAIALD

RWYKVCRPLKCFFTHRRAKWTCGGAILFAASLSWPSLVLYGNLTVPIVGTDSIGVTCLVS

DDFIDTWWPLVFYSIYFTCYLCLVVVITVLYSMIAIKLVQLKKQQKERLAAKYAGLRSSK

LMYASQDNITGIDNNKENLAGNDKPDEVECGVKIHIDCVEGEGIEHQNPPTVDGVLQLQQ

LQEDIERRKSVSFKLPRKSTHCEDMTPEPPGNNLQNGNQLQTSGIMINQITQEEEGIPGV

NREGRDQGKDEGNVHDHYTQTTGSAEDLRETEHLIQTHDPDMMQSNDSLQAHGYSDHAYL

LSKTDNHSYTGSHGDTDEFEQGIHFIAPNDLTWQHSPIVNTVTVNCQAQVTCDDTHSDIK

DVSSDMLADDVGSSSSLLSGKTEQRNAKTRSDSFTLTLSRKQGNGGKSKVHKTSSDSNLV

IPERHQSLTSARRSFLMLPGGYPGGRTPTSRLPTPTESPSPNGIENRSVILSSKLILERL

NKKMAYRPAKTTRMLFAISIVFVISFLPFFCVVITRAVKGRAFLAALSNAGVVVISIFIR

SSFISNAANPIIYGLCNKQFRTECSHLIGRCYGTRRDVTLRVIEATDDALGSNS*

>TRINITY_DN376819_c3_g1_i1

MKEPQIVMAGTRVEDVEIIDVIDRSTTTIRSVLKDGGINDNGQYVNCSGLLAMDCSNFTA

DLSEYNASVEVERLVRIIVPTIFGVIVLLGLLGNLLVISVVLSDKHMRNTTNILILVLAV

ADLLFIVFCVPFTAASYALPVWPFGNLWCRVVQYLIYVCAYASVYTLVLMSLDRYLAVVH

AIRSMSIRTELNTWLAVAFVWTVILLGHVPLLMQHDVMSYMHFDEERSSCTNVYHLEHNR

NGIIFYACFISFGYMIPLGSVCLMYGLMLKRLHGAVPGGSQSQESIRTKRRVTKMVVIIV

AVFAVCWLPIQIILLMQQLHVYPPDIIFIGIQLASNCLAYMNSCVNPILYAFLSENFRRS

FRKFLCCNTPVSNRAEYELVSVKQTENVTTATALKSSI*

>TRINITY_DN114709_c0_g1_i3

MAVLINVTNSTANYPAMEESNNTESASQLFTILPPPTRRASVSEVFTIFPPPTTRVSAVP

FLPWNNPHNVITYSTFRDIEFVFNCVLDPLLWLLGVSTNVINCVVFYRQGLKDRMNLCLF

SLAMVDTLYLSSNLLVDSHRFVSSIHPGLANKLNAYLLAYCSGPNIALMLTSGALTMIVA

MERCICVVFPMKASTLISTRAMGMVIIFVSLLLQVALLCSLTIKFKITSRTDKVTGMTTY

TTIPTNYYLQNQLVFKIFEFIFVSPILPVATFLVVSLTTTVTVIHLRLALAWRASTSSAR

SKGEEGKEGGNRISQQQAALTKVLVLVSCLYITCSAPSVAMAITRFAVDDEGFMPWGRYA

NIFFAAHDMSYTVAAINSSCNFFIYLWQSSRFRQTLSSCMSCAKVGRLVKGPSEMATKLT

SA*

>TRINITY_DN377463_c3_g1_i1

MMVVRPGNPSLSKVLILLSLFRVSFEIGFFVGPVLEGPSANSQLPEWRDSMGMQVTDCPD

RWTCQGYDYSKLSTSARGYYSLKNCACDELCILLQDCCPDALLPSQSSNPSPKLPTYSSV

GNGTGRADGNPLHNKKAAVAENTIPDFRAVWEESRDSQKKGNELRNKSTSPLINADHLRL

KENNTYTDYSLDKDMFSCVYDATINRANNVSVVTRCPEHYDDMEVTLLCQNVTDDDMMTR

LPVSGKVTRVLYRNMFCAMCHQEPFLYWGVGANCSSKHQLPYDHNSTSLHDLLLDSSKCK

IVFQPPGHDYRWRPCLSDVTDRCNDSFPANTALERHITASCEDVRDRTQRMVFGFMQAYR

NQYCALCNHYNLSSIFCVRLSYDTSIITVPDVRERPSTYSFSILLDINANTGSTTVGHRL

QYKTMEYNETASCEEGQVYDPFRSTCRPITCGPGRYFIDNMCKKDNQLTNEGGIFTKESG

IPNMKDHIISKGRNISMTDCSYIQLNDTEYHLFQDGSLELLGTGTFYNVSQYVLEEDKVY

LCVNYSQNYTRTVKTQVQDVTFTFSLTEAVVSTCGICISLLALAVTIFVYASLRPLRNIP

GQNLLSLVCSLFLADLLLLVAPSAGEVFLACAVIAGVMHYFFLASFLWMNVMAMDVWFTF

SKAFVKAGDRGKSSKRFIMYSMYSWLTPVALVLPAALVQILKPGSRVSPQYGLGICWLGN

KYALLLFFAAPLFLLLILNVIFFIISARNISQARRTTARMLGKEEEGKMAIYVKLTMVMG

ITWVLGFLAALVPNNQVLTYGFVIMNTLQGLFICLSFVFTKKVLSLLKDKFCGRRRFKVT

KSGSTDMTNVSKSLSNNRKMSSASTKSHNVVV*

>TRINITY_DN377967_c2_g1_i5

MAEMSLLLPVTSLSTHLTYFNRYCFLCAGDPGPRVTWAVNVSCTEWVDLQSLGSETEVLQ

AALDSRLCTLDLQPPSDIPTETCRNYIVPTLPEVPSDMQSAPSGSRPHRLGAPISVLLRF

RDRGESGDGRGGPERAGGGGMKKKVKESGGTIPEAGRNSSCSGFVVDILDEDRCLTLHCA

PGKRLEGNTCRPVIGRSRGLHYSLTLLMDVFVRSSDVSGSMTSQSLSSLCSLEELVKAGV

TQHTSHLYSEAYDLTGWILTDHADTIMTSLDVVYDEADDLYFDDPAVTSEYNDENVVTVK

EFRLGRVLVWFDFFVTSDTDRNSVEQSAMRMSEEEWHVICGNTHARLVPTLIRRENVNVV

VMKDSPNPNVTDALPEVFTESVTHRDFRLLRSQGSSVALTVSPLLMCPFLTFPGQHYEFE

KLSSVEEHKMVNLTLMNVTQMVPASDIETDDEGQLKMCASLFEEKFEEALRARDDPNIDS

DGKEVSFWALLEHYVSLVCVCLSLLCLFMTIVTYTLFRRLRTIPGQNTMALCFNLFLAQA

LLQFGVNWTELRGGCIALGMLIHYFWLTSILWMNVCSFHMHRVFTTKCAARMSAAHKRCC

TRRVLAYAVYAQGVPLVVIGTTVSASIFLSEGHQVGYGGTVCYLSSAVLVGAAFVGPLGL

VLLLNLVFFLRAVRAISKTEMPVEANRQNGHRSPSPNINLTSPFPSAWRRNLNACIRLSS

LTGLFWALGIIAELLDQRILRLISVMVNGSQGVLIALSYLANRRVMRLYSQLCCTCRAQK

QQSRSSTTVTGSLPSASMAVTQSTSVLTVSRDTNGHLHNSDVAQP*

>TRINITY_DN378060_c0_g4_i1

MNMGQTTCCYDLTPDTAHRSQSRTRLRTHRGRTKHGSGLVERCVVFMVLILYRVSPGDAL

EAHISSTDPSGVIVLNTSLGHNWGYHVDVHKSQSSLPFLQLDSTTGLVYWRGEAKCPHVS

NNPLKLHIVSRTWQWPSQSGSNSTVVPYSVFVHGCEQSHRTLKYKRLKDDVHRIIVNSKI

TTSDCIPPLTSLLNMKDFVPHSFHQCQVSVDLPVSSPVSVDDNHVFTSAVKLCFNNERVV

VKFSVSVLCDQSQEFQVPFTLSIHPCSAVTQAVHRRPRRSVNNQPRFDPASYIVNVKEEQ

SPGVQVVTITATDTDTEGAGTLTYSMIPTRDERSQSRFAINPLTGTINTTVKLDRENIKA

HYFLVIATDHGVPARSASASLTIIVDDVNDHAPQFDKQVFNVNVAEKQAVDFPVLSVQAT

DQDFNENSKIRYSILNPASPNDAFKIDPVFGAISTMRELDRETHQHYELLIQAVDQGDFS

ERKSSTATVNITITDENDNAPVFSQTSYRVNIKEDQAISNTEPIIKVTATDADIGQNADI

LYQLSGNNREKFHIDANTGEIFLVEQLDFEDEDEYQLTVRAEDQGNPPMRGMATVLVHVI

DVNDNAPFFTSSSYTADIVENSDPDASIIQVQAFDRDSGQNSQLTYSFVSDNDNQSLPVI

IDPLSGWIRTSGKIDREHKQEYSLVVKVVDNGDPRLSATTTVVIGIRDINDNAPIFTSRV

YQATVSEEANIGDEVIRVTAEDKDEGENARVHYDITSGNIGDAFLMSQSENQGLITVKKK

LDARQHNRYSLTVTATDTGGRKDTVQVLINVTDTNRYPPEFQATPFQFSVYENIAIGTTV

FKVKAIDRDRGENARITYSLHPGVKAFAIDPNTGEITTRVALNRESQPGYMLRVTATDNG

KPQLQDSETIDVTINDENDNKPEFSETIYSGEISEDAVPGESVLQITARDYDKGANGQVF

YTFEGGNDGNGDFSIDSAHGIIRVAKYLDRERIPNYELVALAVDRGTQPQSSSVIVRIKV

NDVNDNHPQFEADELNVYIAERSPIGSTVAQITATDPDEGVNALVEYSFDGGPDVNSFQL

SGKRGEPAIITTLIPLDYESEKKRYEVILRAASNTQFSTTKVFINVRDVNDNVPKLQDFI

IICNNYGGNFPRGPIGRIPAFDPDVSDQELLVYKIVSGNEARLLHLNMSTGEITLNSRLD

SDVPRNGTFKVSVSDGKNEVKATCRLYVRLVTPEMLHDSVTIRLNKMTQTAFLSSLLKFF

TDALAIIFSTDPTNIFIINIEDDTDVAPAQILNVSVSVRQGSVTVQGRTQDVFFSPEYLR

EYIYLYRSLLANLSALQVLPFDDNLCLIEPCPSYSVCQSMVRRGEPMPFISSNTVLFRPI

HPDNGYKCACPHGFAGKFQANDCDVEVDLCYSMPCQNGGTCLPHESGYTCACSSGFTGQN

CEVNMTMRYDTISCPQDYCRPPSVCVPLIRGGFRCDGCPDDGNYDQFCRLKTRSFKKGSY

MTFPSLKTRNRFTIKLMFATQEKSGLLLYNGRFNEEHDFVGLEIVDSQVVFSFSLGDNHT

SVSTYIPGGVSTGQWFEVILQYHQRVATLTVGENCDTNIAIFYGDKLDNYSCAARVVHEL

PDKCDNPIKNCHRLLDLTGPLQLGGLPWAPSTGQVTQQDFVGCIRDVYIDNKLLDLNTSV

VSVQTGVGCPQKNTHCQSAPCTLGGKCKEGWTTFTCNCPEKTGGKDCSQVIEMSRQLRGS

GYMSFTSISQNTVHYPWFNGIAFRTRAEEGTLMQIILSTGQVRIQLLGGFIQYSFEDNSV

ALDAVQVSDGQWHYIEARWYQGRMELLLDYGQTQKSAALTQSVSGSSIQIIYVGGYREGN

EPISDGFVGCVKDIRVGNNLNAVLNQPRETNVEKGCHAPNSCDPNPCGSGTCVDIWGDHQ

CQCPPGTIGPKCEDICGEYNPCLNWAECNRPTRGENTYTCECGSRQSGRYCQNVSPLECP

AAWWGNPVCGPCLCDTSRGFDKNCDKKNGTCYCKELHYQPAGADQCFPCNCYSEGSRGPN

CDPHTGQCDCRDGIIGRRCDQCDSIFAGIDLKKKTCRVKSYEDCPRNYAKGIWWDPVPFG

DTAAQDCPEGAFGTARRQCTQRTSWLEADLFNCTSDNFTRLQTQLEALQAGISPFNADLA

IELLKNATNSTTPLYGGDIVMTLEFAKLVLEYENKQHGLGLISQQYRDFVQQLVIALSNV

TSLPNAGFWPRINRVYKGSATLLQLLEQYLHNIAKSMAQAQSGPFEAVSDGIVLGADWMS

VYNVTGRPLPKYDNIINKGSANDFASILLPSSVFGSNSQRKGYYGYAIYHTMGKLVEMNT

DSSIVKTGLPLTTNGPMFTFTHLANQNKLSEPVYVRLKLAAPHNRTNLQCAIWMPGSDGK

GIWTGSECSVSDTDCQVTSESDECQEVYITCMCYHMSTYAIIVDLEDGTLPRAAIIKLEA

LMYVLVAVSLVLLILTFFILISYKALQSNWNSIHINLVFVIVIIELAFAVGINRTSPELF

CRLVAISLHYFYMAAFAWLYVEVLHIYRMLTEKQTINYGSMKFYYLLGYVIPGIIVGLAV

GLYTDGYGNDSFCWLETSELFIWSFAGPIMVVVVLNIVTFMLAVKASCREKVHVSDVSSV

RLGLMGAILLLLLLSVTWVMGLLAVNFDLPALGYVHAIFLFLQGVFLFIIYILLNRKVRL

CLKSTWYRLQGKKLNMDENLTGTRSSVMSRSALAYRNDTASSTEGGVRINVGISTTSTTS

QSSKSTGRYPPRYRDDFGRSTSSSTSAGHVPSASGQIPNTGVAPYGYDPNMGEALPEEEY

EAQSPTKPEGNDSDSDSDASIDRNSLDLASSHSSDEDDDFEIGPPWDQHIPKSKAVAQAR

EQLEKHKKEKERERLGNNNSDQHWADSGQVDISVDLDPPPTSRPHISSVPPDVTLSAAAQ

RSGRKVTIVPPARSDSLQNNYATSSSNVASDVAPSGRVQVQVLTHNGSLSSDSESSDETN

V*

>TRINITY_DN114661_c0_g2_i1

MSGVTLSQTTEFSPLSQNTQSTHLVYLEPSASPEVSHDDDDGDVLLSADVYWRAVPAMDQ

MLCALQVIGLVLNTANIIVMTRRSMRSPTTTFLVALSVLQLVYITVGLVPAVHRIFKPLV

ITDAFYLINSLYVSNYGMTCLRRSMYCIQCCVSVERLLAVWLPLKAKQFLLVRRPWLFIL

ATPVVVFLTHIHITLKLEVFETTSPQNQTIHSFRYTQHYRHHSELFDSLSITMKTIFVYL

TLVMLITTNLAMTGVIKRYASMRRQMKTNVDVDAAQKRETQMTLTILVSTLIFVL

>TRINITY_DN378396_c4_g13_i1

MAPQVVELPEVHESLLRLVTIVGCIISCLCLLASWITFTCFTTLQGERNSIHKNLVACLF

IAEMLFLTGIDQTQNRLACGIIAGVLQYMFLAAFLWMMMEGVHIVLMLVQVFDASRSRLP

YYYATAYAPPVLIIAVSAGFYHQGYGTDRYCWLTTERYFIWSFAGPVAGILFANTIILIY

AMTMVCRHSEYVFGGKEKSTAGSIRSWIQGALALEVLLGLTWMLGFFFINQQTVAVAYVF

TALNTMQGLFIFIFHCLLNKKTRKEYQRVMKVKRRPSTTGSGSGTHSSTARHKLTSGPSF

NKHSYHLDNPPHHYFSRA*

>TRINITY_DN378744_c3_g3_i1

MSATISNHMNNRVQSMKDSQVVSSFFNDTGSYLKALEDAEALDLLPVVILLALFAVLGNV

GNGITLYVYYTRFTPSSTRTYIIAMSVFDLLFTSITIPGEILDLRFSLTYSASWLCRLHR

FLSIFLTVVSALILVAVALDRRRKICFPFRLQLTARQVSLSVVGCIVGAVVVGFPFGVLN

GHHSVVTKIEGVTGSACSVDDEFIDTSFPLIYNALLLLIFLVSVVIMSVSYVQIARKIVK

HKRKSMAGGGATVRTLAILMAKQEASMRGEGADTQQSSSHFEDSSVMDKNHCRPQENNNF

HTSRLESKVEDEVFTTDMTGPTKLTSPSTSIDTKKSQSKKNTFKSHSTRKREKEFEMAVN

TLVHAVSEDMLHSSELFKTRALSYSTCKTRSASVGDECSSCDAKKRPDSASESPKSRGKE

SPVEKNDITAQFGSMNSPTTPHNFEESNTDVINTPPSPEKDVNGTVLTQQENMEQTEVKS

KADVMNTVTSSENEANGTTHSGTQGRDKIQSKGLKKSSLKSSKQNDVTGNDDRHVTISET

GAERLQSILRHTLRRFKSGGADSLGGGDGDDAGGDGVTMTSTLRRRRHWGKAIPSRTTWM

MFVLTAIFIVSYLPYLTITCVRAIQHDVETGLSGWKFNLYILGLRSYFINSIVNPVVYSF

CSARFRHQCRHIFRRRPKMSISD*

>TRINITY_DN379018_c0_g1_i1

PAMRYIWVSAHWLAMSSCMYNPIIYWWMSEKFRCGYKFLLRKIKYKCCKRGHDHLMMMNG

WGAPFNSSFSQAYDLQNRYRSSCAGIGGTGERIKLSETWCLGVT*

>TRINITY_DN379645_c0_g1_i1

MHRALFGMLLLTSAVMQSHSQTMNMPYDDTESSGEVNPDDYVDNFGSGDKDTPSLETLAG

SDINTQTVAEVINQTQKLLANPRDLGAVEILQTAIIIERTTQVPSIPKQTAHGVLKLVDT

VSELNTDLLSEADTLSNVSNRLIRSVDGLGQKVDLEGARSVRLVSTTMALEVWDLSNACT

DDLAVGLQLKTVDDDDDEDNVVMSRRAVVESSHLVTVRRGERFLTSETDTAINLTSSFMK

SVVKENQGKAIRMWMTVFSDTTLFDHNTSWPTNASDSGTLKTSKRRLNSKVISASVTADR

VPVSGFPSDVVTTVFLPLVQLTAEQRSLYSRCVFWDFREDGGQGGWSQEGCRYLKTVDGR

DVCVCDHLTNFAVLLDFYGESELPPDDHQDVLSVISVVGLTLSICGLTCTILTFFCIKKM

HSILPKQVIFNLALSLLCSWVVFLAGFSRASGSHGGCVAVAALLHYFILASFLWMLVEGI

LQYLLLVKVHGHINRFLLKASLIAWGVPVIPVITILAIDLELYKGFTNYCWMSLTPFYFG

FLLPVGLVLSVNIVFYVLVIVSICRVGSGIKSGKVHNRVKSTTVSVRASFACFVVLGLSW

LFAFFAIADARLVFQYLFTLTTSLQGFLIFLVFTARDPHVREFWREKTLRGMTMLRRVGR

SQTTASTSVGSSTGKSGDARGPRAAADGTDLVTQKDSSSKAPESGTDVPLQTLSV*

>TRINITY_DN379815_c0_g2_i1

MAAVSSGVKISLLTLFCLFSLEFSSPVSAKWVKSTTDTKDDWVFITRFCFLSELGRFDYV

FKYPTSYGIQQLLLYYDKPGQWESVYKTSKNCSTRRKVLSKENNQIIDLTNLGRDITGSR

NAGCTTYQQDGEEYFNCTDHVSFRAARERWWYIVLARCEPSQGGKVGLKMDYEIHMMNGP

DDDYLHHELSADEFYILPVDIAFLLAFLVLLVLSIICAVILFNRQMFHTTYKMYVTALSL

WMFGLFLQCIAWGRYGKTGWEEGPTEVTGRLFEAASTAVFLLMLLLMAKGYTITRGRLTQ

MSTVRLTIFFCLYIIVNITLFIWEGMFFDEGFVLYFYESPPGYGLIAMRIIGWLWFLYAF

IFTMKHHPNKAKFFIPFFIFYTAWFWAAPIVILIAIYSIAKWSREKTVNGVEQLVALLGY

LFFLVLTRPSAANTNFPYHLRVSQVRFSDDDDEPTETYEMSEASRRHLDSMFTVSGNGSS

SSSSSPPSYSSASGGYVNGGYNGYMKNENGYNPVLESTFITQRNNQVPPLGTNQPLPPLN

SGPTLPPSYNDQVHPPSYERVTQLPPIRNNGPEVPPPQENEQPLPSIRNGGPRLPPLHNG

LLPPIPRPPSPSAPSAEE*

>TRINITY_DN379992_c1_g1_i1

MEDSLILENDLRALSPQDLTNLGETPYSLHDLLHLLPNHHHHSYPYDQLPLHDSLASRPK

RFVVTHSMIEQGRFNVALLVLFIICIFLVNLFFIVVILWSRSLRSSSKHLLILSVAVADL

LQGLLILPLITDLGTRRGGADFDCTTFQVARLLADFLIPSITTLGVLALNIDYILRLTCN

AYSEGTSRAVILCTLFVTPWVLSIVLLVPIYVVGVQKVSLYNIFETCHIVFVGGYARALL

VLSYILYGFLLLIVTVVVAVMYLVKREQLGLDVTGERVHAPFDICLASFIVVLFYTPIFL

FTILTTEGYLGCSGDSECRVLNWSYTLSLWLMFTKSWVVPMAWFFGRDTRKGIRDIFTFC

*

>TRINITY_DN115650_c3_g1_i1

NSETQGMSTPPKTRKKISGPLASFVINYGSILYGTVYVSGFISTLIAFERCICITYPFIT

KQILKTKTMAVIVMVASVILITLMYLVSAIKFRIGCGYYPPFNLKLDIHYPSDFYLENKT

VLDVLNGIVYGICLPVIFGLATTITTIITAIKLKGAAAWRQNTSSVTMEAREIALTRMLV

VVSCLFIGCTIPNVVLRIAPVVVKELKLGGRHQNMLMSGVSVVYLSNTVNSAFNFIFYYK

MGSKFRMTLIGLCCSKQRDSEPVVPAKAATGTHQCLQTD*

>TRINITY_DN115650_c3_g1_i3

LTYPVYCARKKYAKKMNPNLRYKNLSNISAVHMSLSSTSSENIHHRSTAAFNDDLPTQNQ

SDISAECRVLEFLPWSNPDNVITFQTQLLFFRVFGGVLIPCVSLFGIPVNILNCVVFFKQ

GLRERINFLLFCLSCSDFIVSGFMFVSTLEYFYTQILGNFQISGPLASFVINYGSILYGT

VYVSGFISTLIAFERCICITYPFIAKQILKTKTMAVIVMVASVILITLMYLVSAIKFRIG

CGYYPPFNLKLDIHYPSDFYLENKTVLDVLNGIVYGICLPVIFGLATTITTIITAIKLKG

AAAWRQNTSSVTMETREIALTRMLVVVSCLFIGCTIPNVVLRIAPVVVKELKLGGRHQNM

LMSGVSVVYLSNTVNSAFNFIFYYKMGSKFRMTLIGLCCSKQRDSEPVVPAKAATGTHQC

LQTD*

>TRINITY_DN115321_c2_g6_i1

MPDRMAIRNNTTVQNTWPSNIHLPTVVPNNSIPEGCTVQRLNKLEYLPWDNPDNIVSEEA

ETIARRINENIIVILFLIGAPANIINMAVFYKQGLKDRVNLCLFALSLADGIFLLQNMFL

FGEQVLLQFTSFEKYGPLTTFITNKNLVGFFGCQWVSQILSAIIATERCLCICSPLKFQT

LLQTRTTAVIILTVYILVIGLCFFVSLRYRLGCVYDLVSGTVTKDFVIGEFYETHKELIN

YLDNFIFGVGIPGVAITVVMSTTFITIVKLRQIMTWRAGTSSSISACEVALTKMLIGTSL

LFIACITPVG

>TRINITY_DN115321_c2_g6_i2

MPDRMAIRNNTTVQNTWPSNIHLPTVVPNNSIPEGCTVQRLNKLEYLPWDNPDNIVSEEA

ETIVRRINENIIAILFLIGVPANIISMAVFYKQGLKDRVNLCLFALSLADGIFLTQAMMF

LRGEHLHLQITSTKKYSPVATFITNNYLVIFFGFQWISPILSAIIATERCLCICSPMKFQ

ILMQTRTMAAIIISVHLLVLGLYFVVVTKYRIGCLYDLVSGTISDELVISEFYRTHKDLI

NYLDNFVFGAIIPGVVITMVITTTVITIVKLRQIMTWRAGTSSSISAQEVALTKMLVGSS

LLFIVCTTPIGLFRTVCLFFSDINIGGRNQNLYMTGLFIIQMFSYINSTFNIFVYYTMGS

RYRETFWTLFSRRRCKR*

>TRINITY_DN115214_c1_g7_i1

MMSKSDTLTPTLNTSNDVVPSMYTEITNTVLSVPDGCVLKQVSQFIPWDNEDNLVPKQVE

DIVNILFTGIVLPLLFLVSFSTNILNMVVFFKHGLQERINVCLFTLSLLDCVFVTIAYGY

SSDVLYMFAIGKGDEIGAAAKFFIQSHLIGLHGLISSSQVVSTAIAFERCLCITRPLLVK

SLMSTKTTAVLLWTFVLLILGGMFVVAGLRYGLICIFDPEDSSTLIAMYPSEFYLQNKFI

LDFMYGFVAGLFFPGLCITCVTVCTVITVAKMKKLSQWRETVSSASHSFTSRDLAITRMI

VGSSVLFIVCFMPGSLISVCFLVVPDLTLGGRYNNLYLVCVRCFQLASTINCTFNFFVYY

VYGSKFRETVHQLFSSYICCSDKKHTESLTSNR*

>TRINITY_DN381479_c0_g2_i1

MACIIWVLAALLCHAAAEFESQTCSVDRQATIQAGAEAMLGGIVAIQERGASGYGCGKPA

GSMQWYEALRYSLDLLNKKDEYLNGQLLTDYYVPGVRLGMHVIDNCNNKDQAVAAVSTLF

PSLSADDRSCATAQSNMTLGIVDSTDSATVASIAGFADNFKIPVVALSASAKGLTFSGMY

PNFMRTVPPDNELVKVMGQVLTRLKWKYVVVIYENSLYGREAYSALRPVLAEAGICLTAA

FMAESSDTSSTTMRNLLGQVVATDTVGTIFLGSDRLISALLKQGSSVNGAGKLQWIVTDS

ISLDSTFTYNYPRGILALVPASRYIVEFEDHWVRIDEENPSKENPWFRDWYMEKHQCNLA

GVSTYPKQCSSLYQGMTAAEIERKKRLNFVQDQYVEPAVHAVFTYAYALREAQQALCPQK

TGICSQLANLKYEDFLNTYLKNVDFTYAAQERVPSLASDQYAPYKAAKRLQFDARGDIIN

PSFSLWNYNDLPTGEENSTIFRFREVGNYINKRLTVNTDNIRMYTENRLTPLSPFPASPC

PARGCSPCLGVPQEMKYYFQNGDIVINGIFSLHKMGQAKFTCGQLMSNTHPLYLEAMIYA

VKKVNAQNILRGVNLGGLGIDDCMDSDLSSNFIMQVQRGLYTVQDGSGNALDPRKVEAYT

GAYDDPLTIPLAGLMDHFMQPMVGYRATSAMLDKYKYYLKTVPGIEDEFRAIIMIMKSYN

WEYTQILYSNDAYNNYDVMLFRRLATQAGICVVAYYKFDSDNRMAASMLSHHSTVRPVVL

LLSENNHRALLDVLNSTKTRNKNDFIATSTFSNNDEITNDYKSVAEGFISIDITYSDLTQ

FYNELSTLRVNTYQRNPWFEEWFESAFNCYVGGNPRGYTSACNTAVGITSAPGFKRDLRV

MHVINAVYAIARGLDSILTKYCGTGYNGVCGQFVTASRANKGNDLMEEILKVSFPIEDTN

PSQTFRFYNRGGAYTYTVYSFSFGQFKKAGTVNPYSYSMSTLTTGLGSVSPPTCPKPCTE

CLYMFAFQQYWYLDGDLIIPAVFDVHYKGMSMYGCGPLRVNNGVQYTEAFKFALDLVNGG

TLVNLNNVRLGGLAFDGCTSPARSLAIINGVMGRNFPIMDNQGGRVDISKFVSWLTYDSE

STMEAADMLKMIGMPIVSPGATAPQLLDKTKYSTFFRTIPSDTVIARAMANLIQERGWKY

VLTLNAPDAGNRKTRDLFRQYLKDMGICVIGNLEFETDGSVDVILTSIQTSTTHVVAVFA

DTDRYIPDMIRRLEAQNNPNNDIIFIANRYWNLDHMGIARTWPRASSVVTNTLSFWLEDS

TVTDFMNYLNGLTLTGSDNPWLMEYYQAAFRCNFGGNTMYTTSCSSTSLNLGNSNRLYQN

MYTLTTINAVHALALGLHQVLIDKCGAAYSGVCSRFLTDSDTLNLLMAKMDAETFTDVTS

FLFDFIDREANRGFDIMQQDKEGNTRAVAKVSMDGNVTYLPNWDNSQYANVKSECSDDCL

VCEGFDANFRNFSFIDGDVYIVGLFDIHTEGATPYTCGTINQYQGLELLEAFNFAIDYIN

KKKEIFDGKLTSVRIGGIGIDVCKSPTRAASLVANIHSGNIRLSLSGGFEIDPRQILAYV

GPFDTDSTIQVADILNAIGVPQVTYGATGLQLQDPVKYHYLLRSVPADDKQARAIISYLK

NFNLTNIQVVTSEETIGQAMTQEFLRLATLNLVCISHNYTIGEVFSQDMSTQAMNIVNEI

VRYDQSRVVVLLVDDPLPILQAAGSNALARDNILWVATDKWGYDTEFLEKLGPLLGDRNS

KKNVIIFDIETADVPPFDIYLQDKTPDNYVRNPWFREFYEDQFSCSWAGTGTICDKTRGL

PRLDEYIQDPYVIYVVNAVISVGLGIDGALRVICSDANSLSIGGLCPKFRITGNRRDIVL

AEMKKVNFTDDTLQPFYFESTGESSRGYHIYNVTANYDNILPGSYMYENVGSYNDTHFLK

LDITYDLNYQAYCKPYDNCMCVNIFPEIIPSRYMLVPSQFELNLVYIGDIHQPDPRNPFA

CSTIRVGADFFKMMAFFYAIDRVNRNLDNRYPESLRLGGIALDTCSSTLRLEQDIFNLLS

GYPLCDTGKSGQVVPPSSIIAFVPDGNANSIPVSQFLASTGITSVSPSATNPKLREFVMS

EHFLSIVPPDDVQATVILKVMDYLNWNYASVVYTDEPSMVAAKNELLRQAAVGQAACVGQ

AVSLPLDADISDAESALEKVSQQVGARAVILFTLPAHTRLLLQASQNHGQAGDFIWIGTN

AWGRNNEVIKGLEQQASGAIVLQPYSMLVEDFRTFVKSLTFTNHRGIPDDWFEEIYQTIH

RCKIQDAKRSLPFSKLCSKTETITDDMVPFEPSVLHTIIAVNMVAQGLNQIPACQGSRFA

ISACITRLQNRNDDIYQAIQNAQWNVLPALLGDDSFTFSFIDSGYGNIGFDILNYHSSST

GSGYVYSRLGRYTDSLDLNSLGYQGLSSGDLSVPTSNCGGLECFCQGPTGIYGVPRQWTF

IPGQTSIEVIEEGRTYRDPETGELFYVAKIPDVTTRFKDMWGVAMATLAALGVFVSLALF

IYLLVVYPVRGGTSVLGYILSFSIVLLYSLVFAFVAHVNVELCGLRRFCLGFCYAISYSA

LFVKLVDCWRSREKEDMFEVKYSKLGRPLGLFMVTVLLVLVQVIISAEWLILEEPAVVRI

FYHDQYWPRCVPNDFYDQGLVLSLCYIMVLILLTVLLGFCTFNSTKNHREARWILGIAIL

AVPTWVVWCAWATLGAIKTRDAAVAVGLLINATVLLLLGPVRKLYLLNKYQALIEEEERS

NLQQERASKGNDYSTVYENQYDNAPQLHDRSSGIGSTRGGYRYPPSSVRSGR*

>TRINITY_DN381780_c1_g1_i1

MMPNGSLATASNRDDSSWVRVKENGPAYQHQDSSQKGGVMGSETHRQEAYYLFFMGAVGI

ALNLLVIITILVRRTLRKMTSAFLIHACFLNLLKSAYLIPFGVNLLGDAPPADCRFEGSS

YVVIITTSAFNMVAMICAEAYTFGENNVGGKSRGSFCCVLFGVLMVYVCSVVLHLGPTLI

GGYFQYNPEIGNCSFKMGEVTGYVANVMWICIVTLSLVAVAHFLCKMYKEIQQNQTNRVS

MLVRSSITIMDDAEAKRNSCCIRAMVKEASHRAKIFVITVLAYVISWYPLFLLMLIDVDF

RVSPKVYQAFSFIAWTQGTVEPIIYICFDRQLNLLARWVYCDRYKQYDSSTLAHLMAQNR

GTVAGEDTMSNAIDGGASRGAPNTTATTISATHNSIAYGDGGAAAYPYHEDDDEGLSEGS

TGHPNTPPPPPPAPVDLESTGFPQSAVVHGVMEPMYDNQRPSSSAAAGVGRSEGSRHHSA

GVGRSEGSRHQVSANMEVPVPHEIEC*

>TRINITY_DN381897_c0_g1_i2

MDLSTNELTTKPLATTSATSPLTPTTTVSSRKMSVNELTSLWTTTEILSSVHHDDVTQLI

WTTGVTDFAREENVTASANSSIMVSNNSSVPAMMKNDTILDVNLEALNLSMTMSLLPTVI

YLVVLMAVGLVGNSIVFLVYYKRFKPSATRTYVLAMCVCDWLANAFSLPSEILDIRYTFT

FDLPWLCRSIRATNSFLTLTSAFVLVAVARDRFNKICHPLEKRRSMKRIRIYVLICALAS

VALSLIFGALNGSQTIPTGVGNFTGITCSVSGEFLDTLFPLMYNMIMAVTFISCVVIMTV

SYIRIALELWRHKKKQARVSGVAPTPSKDHSKTHSPIVEDTSSGPRHDLHHSETKSSSTA

ELIPMKTTSGTKSKSKATSPATTPANTSDKVLLLSVNNLNYEGPKSSVEDSDEGCFSGCS

AETTSTSVDVSKEGTVHTNQATQGDSQTANVHVHIVDENEDKEVKKLDTDTETKPEVPES

AQKNGSGSLDATAIEDHAMTPLDSQDASQDSQTKTSTIQDKHDSDDGSSNHHRHTDDSSL

SPHHKTTTTTTKPSSLWQKFLMSRRRHQYDFSKESALKPSQRCQIPKKRGTVKSIPSSTT

LMMFVLTATFVINYLPHLIIISMRAMSDDFGKGLTGAMLNAYNVGLRSYFMNCAVNSLVY

GFCSARFRQECGLLFSRK*

>TRINITY_DN381970_c2_g2_i1

ELFRVSVTVPLVTRPADWNEELATTQAITVLQLTGLRTSKRFQFDGVNSSTGQDIQMLHF

YVDQSDLDMVQDGVKNLMRRDTLFDLTVISANCTVQIDPEMKLLSVENYVAEPALEGKSL

TLVCTARGSKKLQFRWYKDQFLFNSALTSRNAWEVRLQDDFDDKQMSIFNVDGVTMFDKG

TFSCEIEDFGEVENRSLTVDVMPLPQVEIKPLTANLLPGQPLSFRCLSPDENMRTFDYQW

LRDGEDVSEKERVNGEIVEDLLPSGSRLYIPSLAVSANYTCRVVNKAGAAEKSAFVFLIS

PNMSEMACESDSYEGYKWNRTYGGYFDLQQCPIDKADFKVVGLEEGHARRDCVCDTTCAW

GPPNYARCHSIHLIYQYEQLERCQLGYQQDGLSRIYDNLYKILRKAQNRTLAGDVDMFAI

ILYTLFDVALRFPAVAPNPAKSSFNLNTVASFMNMLLDEVKTTNGFEKRDLSVGARFIRV

CEIIAEMTRENPTLIPVFNVTLPRIGFAVRNVTVLAHNTSEESEEDAEDEDGTPVKMSYT

GRKLMSILLDGRSKGEASVKSLQVLQVTYSPDIIAILRVAGTRSDFKEFLTSLVSVLPVG

ESDLLDDSYKVKNGKVELEFVHPSQFPVFVENITKCVAWHRRDSRQLVGEWKTGVCEVTH

RTANVTRCLCPVPGHYTVVVIATNTTLPAVLPVQRDTVLLIGCVISLCGLVAAILVYFVC

WRHLQEDSSMIHANFIMCLVGINVICLLCLSHSNSEVLCFLGKVVLHLLQLTAISFLLVE

AIHTYVCIQSSGFRGLSSRIKYTCLKYCLIGWGIPGVGTLAVTFMSEYFGYDDTCSSLCW

WSTGTWQYYSFLVPLVVMVVVQVFIMLTCIVCTRLWKDEHRFRDRKKYIFIAVRSYILQT

LVVLLSISGFLAEGDRSLNHQWFFALVNILLTIAVLITLLLLKRHVRRIVFSFLLPAHKQ

ALASGSFRAFVLPDRVEDSEELEVKRIQQYCTELDRTKFTSQHKQTLRTLLGSSSSGSDA

SASGTASPPNSLSVPGHDTFNRRSHSAGSRSSSSEWGGARARAAGTGGADGRKQGGKKGR

GKKPLKMQTTSAAPPPATRREGTTPSPSRQLLCQEGLSPQHDWTRQPPPPCLRVDPVVVV

VGRDKEGGEDQERDPSPNLPDSGYEGTESDNPSLRQGALPYDLDSQVSSDLETEPSRNGN

SPSSSSSASSSHPNPLLNPSATPHQHSPSPSRTTPHSRRHGNSLTAEGGARGGTDVMSGD

VRVNCDVISRANASPTNEQNADCEEAAEREALLCVESDCVEEKESEQQAFVTA*

>TRINITY_DN382052_c1_g1_i3

MARKASISSLLMSFTMAALVMTSLAVANELYEAVADAYMCAENDDDWSFLRVNTLSEEIE

SNPSRAEYLCYTVLQASGFEDALDQCHQQQASVLSFTQGVTFYEIPGGSSLTDLLAYNGV

KNLWLGLYVKDGRLRRDMFPMTQLMESNFAFNVRNILSLGKPSPSEPQGTSNCFEMALNL

TYNCPTPVTDSPSGNGGSGGNPNISDVFTPSDEPRQTEPLPDLVTPGGEDVPPTPQGSST

QPTQKPPPDSITSGNGLSRRKRSEIRDILEKLGSPRANTKFKVRTRRQSYPDVLISPWSN

TKIEVIARRLSYPAVLTSSKLDMKFKARARRHFNSAALTPSRSNMKVKVRGRRSLGSEGG

LLGSACGPGTVSVSVKSRHCHDRLSAVCVKPALRKSSTFLNRSSIFCESGQLGNAYFSRC

FSYGLEKVNRSAAQSRCETQGSDDRRIRTLYRPLTKAYSSFLDRRIADAVIHDLGNNATQ

AFIQTSMAWVGKRDKNGGDCLALSLDTGDIVSWDCSALLPLYICQSGVFLPSVAKNLSIE

IGPATAFQIQNIAGGDDKQISVPEEYTLPGANVVPKLSCVYHSQLIPDLQSMTLVKSGVA

QVQSSSSVPLFGKISALAARNVSHRISMNLRSGFFTPSYNMTDYYWCEVNDLRTGQIHQS

GKFFVRTKGVEMYAASITAILREYSPSQVILSSLLGQAQQNGPLSPRSSYFGVFDFRVLQ

YRHYPQAQRRNRVVVDFHFFNTTATKNERPVTNQTLREAERLLRSSFIELSEEILEFDIN

TLRVRSIDKCFYTTIDDPVTGKRYEIPDTNLGEIFTSPDICYSNGEPFVTLKCEGDKLFG

ASWSEFIPNAGCDFTDADTSASTATLKNISESDINEDTVEDKLAETIDIVSDIDGFVPVD

VVYLADILQQAAGVENLTVGTAEEILKLVDTVAKIDETVLKESNVLGNATNRILKAVDEL

GDQVTIDGDVSRVVTPSTALEVWNLNRVTGDLIIGLELRQDGEEPQPVLNTRDLVSLFAS

KNLSYTFTDAAILLPGAFVREIQIDNPGKDIRLAMNVFAKPTLFKLGQLDSRDGSIFKNT

TLNSKVISAKVTVDGVGVSDLDGSVVTTVFLPRVRLAPESQAGNSTCVFWDFAGGEGQGA

WSEEGCRYQSAVRGRDVCVCDHLTNFAVLLDVYGQSALPQAHQAALSAITIAGLSLSIAG

LSITIISFLFIKKLRQGKPQQTLFNMALAMLASWVVFLAGFSRVENHTGCLVVAALLHYF

ILASFMWMLMEGILQYLLFIKVLGVDFHNYLLKTAIPAWGIPLIPVIVVLAIDTELYHGG

KQYCWMALNPFYYAFLMPVVMIMLTNIIIYIMVVINICRRRNKNSSGGNSRAVGIRASVA

CFIVLGLSWMFAFFAVDDARVVFQYLFVITSTLQGFLIFLVFTARDPAVRAFWKQTCCRK

KKSRSDKPSRSVRIAEPGFRDSKSDDSRLNRSGQTSGSSQDQMLSDRTTPSNLSPTSTEE

PKYFL*

>TRINITY_DN382054_c1_g1_i1

VFISAGQAFIYWSIQKNTLTIKTTKVSRDMTIARRLVSVAVTDFMCWFPIGLCGMLSMAD

IPISGEVNVALATFVLPLNSALNPFMYTFNTLAEKRRKSKEAELLKWLESHADLTSS*

>TRINITY_DN114661_c0_g2_i4

MSGLTLLEKTEFSPLNKSTPPAYLYTESSMSPKVSHDDDAVLVSADEYWRAVPVLDGAMY

VLQVIGLVLNTTNIIVMTRRSMRSPTTTFLVALSVLQLVYITVGLVPAVHRIFKPLVITD

AFYLINSIYVINYGMTCLRRSMYCIQCCVSVERLLAVWLPLKAKQFLLVRRPWLFVFITP

VFIFLIHVPSTLRVEVFETTNQRNETIHSFRYTEHYRSNRKHFDNMGIALKTIFVYLTLV

MLITTNLAMTGVVKRYASMRRQMKTSVDVDATQKRETQMTVTIMVSTLIFVVLCLPTVTY

ALAVDVAPRSYGPFSVNRYFFLFMQKIGGVLFGVAFFTDFFSYVSLSSAYRNTLLCMLKI

TKEPEALFGTRSGLKIKTSSHP*

>TRINITY_DN115848_c1_g3_i6

MSGLTLLEKTEFSPLNKSTPPAYLYTESSMSPKVSHDDDAVLVSADEYWRAVPVLDGAMY

VLQVIGLVLNTTNIIVMTRRSMRSPTTTFLVALSVLQLVYITVGLVPAVHRIFKPLVITD

AFYLINSIYVINYGMTCLRRSMYCIQCCVSVERLLAVWLPLKAKQFLLVRRPWLFILATP

VVVFLTHIHITLKLEVFETTSPQNQTIHSFRYTQHYRHHSELFDSLSITMKTIFVYLTLV

MLITTNLAMTGVIKRYASMRRQMKTNVDVDAAQKRETQMTLTILVSTFIFVLLCLPTVTN

SLAYNAAPKSYGPFSLNRYLFLFMQKFGGLLFTLAFSTDFFTYVALSSAYRNTLFCMLKI

KKEEMLISTRSGSKTKTSSCP*

>TRINITY_DN382275_c1_g1_i1

MNRLHHILLLLFLTQSLVREAHGLFDLGMGMRFPHKPLLVLGSVNLQPVNSSSHWGDLLH

DTLQHVVEHCKSARLCNTTGLDTLHFYDSKCSECHPCSCDEGCYLRGDCCIDKQLRDVSD

KPVLNSQLHKMSPTSGCHSTYITQYDLATYAHMVDKCPTDFHDKVVVDRCLNPSPNGTFS

QWPVWSQVSNTVYRNADCALCNNENMDDVLRWNLKIQCDRSIHLPQPDKVMTLIETALEE

PTCQILFSPPEGCQPFPCRPVTGNYIETCNTTGQRHIYNQALWKACEVFSGKMTVSGNTY

KNVFCAMCNNDRLVAPGDCESDLPFPFFVEFNGSSFQAQAQAEQSAAAADSDEGRCWCKE

HEVYDQYEDKCRPLHCSMTRALVNGRCRSVVTNSTGLFYDVALSLTPTDPHTPVRLTSTS

PLSQVARNIRTSFMLQGSSESGWDVNTHKFRVIAEMKQGCESQGPHSPEGKDQEVFRVIR

FKVHIKFSSNWPEPSETLENRLFALMKSQWNISVDGRPKRSFTASPLYFGSQKENETKPS

QLVSLLSSKGAIKEFVVDIPDPSIQDSGAQTLQTNYYLHISQALKCVKISFNVSRKLESG

NALLVVNKTSGSAHHIPLDKVIGFEFVELGEGGEVSVCADKIFDADADDADVSRHFPVLL

FSPRLFAITTAVQVLSMFCLILVILTYLTFEELRSVDDKNTMGLVATLNLTVALTGGTVF

GENYAGACGCIVLGMIGHFVSISAFTWAAICLFHSYYSFKCAVGSWAEGCLNEHTVFLKY

LALSLVVPSLIVVVTVAGSVIGSQDDCSLNVGYGKGICFLSGNVSIQLAVCVPILVGFIA

AVLFVCAIREHACTRGEHHESSDKAFQRFSARALLAGVMCLTWMPHATALLMEKVRFWYT

FLFFCLLLAIYIDIIVLCEKRVVKLWRSRFLASFLKDKNLRKGHKFTPVET*

>TRINITY_DN115711_c1_g6_i2

FMIGGPANIINMVVFYKQGLKDRVNLCLFALSLADGLSLTIFILLHGEQLYFQFITKERY

GPLHNALMNRNMITLCGFIYVSPILSAIIACERCLCVLSPLKFQTLLQTRTMAAIIIVVY

MLVLGLYFFVAFRYRIECMYDPESGEAFNGFVVGEVYKAHKEFITLLDGVVFSLGMPAVV

TIVVITTTIITTVKLRQIVTWRAGTSSSISPREVALTKMLIGNSIFFLACLCPASVIRFI

WLFFPEMNSGGRNENFFLTSLWTCQILIFINATFNICVYYAMGSRYRETFWALFGRKSDK

P*

>TRINITY_DN115321_c2_g15_i1

MAVFYKQGLKDRVNLCLFALSLADGIYLTTALFLYSESIFLPFSRDKNAIPVITFMVNNK

ITCFMGFCFVSFILSAIIASERCYCVYRPLKYQTLLQTRTMAALIVAMCIFVLCLYFIVL

FRYRVGCVYYPETGLALKTMISSEFYQTHQQFIDFLNSIVFGVGIPGGVMTVVITTTIIT

TMKLRQAMAWRSGTSSLISAREVALTKMLIGNSVLFVICVFPTCLLRIACLFLPEMRSGR

RNHNFFFTCLTLCEVLKYINSSFNIFVYYVMGSRFRETVWALFGRRVSEMKQPDTSYPTV

ITS*

>TRINITY_DN112915_c5_g4_i1

MLLHGEQLHLQFTSTERFGPMTTFLVNINFIGFVGLYSVSPVMSAIIAIERCYCVLRPLK

FQTVMRTRTMATIIIAVYVIVVGLFFVVALRYHIGCVYDPETGVVLKTLIDGTFYRDHRQ

FIDILDGIIFGVALPGVVMVTVITTTIITVFKLRQIVTWRSETTSSLSAREIALTKMLVG

NSILFIACVSPVALSRFSWLFLPEISSGRRNHNFYMTCLWVSTMACLINSSFNIFVYYAM

GSRYRETFWALFSRKSKIK*

>TRINITY_DN92000_c0_g1_i1

MEKRTSRSWIRPLHILWILVVWARTVYTISETDKRNSSEYNKTSTDFPPYPSEKVYFDLN

DYPRWNFSLEGPDNSEEGEDTSEDYGDFTDEPFTVPLPAVSLYDDNPCQNVEKTFMAGDG

TIEMTETLQDCNDVWTWEIQARSPDDGIILQFNNIHLRLACTLQVFTFARGSERVEQRKF

TSRRTDFMYFDPLLLQETRVLVELNAPYSSYKYSFSTVNISYYAHPIAKLPRNTFDASVP

SGSLSLYDCHEGVLVPQAFRCNMVKQCMNNEDEEHCEYTKLGCGEGWAPYKDQCLKMEFV

MTFSYVPGHPHPTFPSVAERTCVSKYGGSLAKLPDQEGLDLVGNLLRQSGHRSAVVGLKK

MKPVSKRLRHLYRYVWQWGDKGSPVAHVQQQLQRDGPLLDCAMISAYPSIHFRPLRCVVP

DIRPEGYVCMRPNHNWTRKEIPRPAGVHFPPPNRQMEAIATKSCADGSLVQTFHRCLNDD

SDEPLAKGFAPFHCRYGPPIHYALVCDGNNDCADGSDEHDCGTVTYPPLRDSIFVCESLQ

KISKSQRCDGMPDCFDGSDEIDCTSCQLSLLSQIMCTGVGCVPSHYSQYFDGCPTIGLST

EGEFLSRIPAVVSFDGYGMSRMLEPGFQNDDGLFQCISGVYIPSFLLNNGELDCQFGEDE

NIPPENFTCPGFYRCQYSGLCVHPNFVCDSIYQCPYKDDERYCNLPCPEDCICEGYAFVC

ANLSEPEVLLQARYLDLSGSDSVPMHALQFMESLQFLNISNCSLDIVSLANMSQLRILDL

SFNRLTNLSSLMFEGLTGLRTLNLSGNPFVTVLDTSFGTFVKSSGLTSVNHLIITDTELQ

SIADRAFSSLTQLISLDIQKNQINFYDKDVFYGLKNLKYLLTDDSKLCCKYFHSAMAECV

APVDELSSCSDLLRLDFFRVFLWTFSALAITGNAAVLLYRAFAARQASSPAFRVLVSNLC

GADLLMGVYMTIIGIADAKFRGIYVARENEWKNSLACKMAGFLGLLSSEVSAFVICLITM

DRLLVLCFPLKPKLHLKGCSSMVVCAIIWLLGLAIAALPLVINLDFYGETSICLPLPITR

RQFSGQYYAFGVYIILNFVLFILIGIGQACIFRAISNASKAAGTRRRQQELTIARRLFLV

VLTDFCCWFPIGLMGLLAFYGIPIPGEVNVWAAIFILPLNSALNPFLYTLNTLLEKRRAA

RLQQRTKKILGNLQSELPRLHPSLAEEVVRVCIRSRIVKKDVMLRWLNLRQDSNMSCVDS

EISTGKTSSFVLPDNTSSGKISHIS*

>TRINITY_DN382709_c1_g1_i3

METFYNNSMNASYDATTMMMMMTTESINDTTTITNITIDTTSNMTGNVENIPDIHPAFTK

LIIVLYVLVILLAVMGNGCVIYIVLSDRRMRTVTNYFIASLASSDALMALVCIPFTFISN

VLVNSWPFPAWMCPLTTYFQIVVVFQNAFTLMAISMEKFIAIMYPFKRRLSRCQCYLVIA

LTWFLSFGTPLPTAILSRVDVGTDDPNATVSFCVEHWPTDAQRFSYTMTIMVLQYFVPLA

VLTYTYIRIVIVVWLKDAPPHGQMGGLGGRPSFLQQRDSTTFSAPPGGGEFGGPGNYVDP

RKRIIKMMMTVVGIYGVCWLPIHIITILGDTNPSIWDMPAMRYIWICAHWLAMSSCMYNP

LIYWWMSDKFHRGYLNILAKIKYTCCRQGHEVMMNGRGGAFESSFSHGHDNPYRHRPIPG

GGGQGGRSYPVKQNGVRVGDAGDAHELRMVTEVEETERERTQNGLSFPCSLVLDKGETVP

LRKASDPTESGLGSEDGEDVEEAGIGPVQTVHPDTCSL*

>TRINITY_DN383025_c1_g1_i11

MEHLTTPVLLLLLISSFSPLLCTAQSGDRGDDEDQVSRHSTFACQYGLLMLECPPHHVIR

IQRANYGRFSSRICNPDGITENMDLQCQSVTAVEIVSTKCDGHNRCQFEVTSELFGDPCP

GTSKYAEVKHYCEPVKSEGDSGLKPLPDAPHPPRLPSVPVPENIQVCQPPMSRGSIYDLT

WQMPPTDQIILHFGLKYRVNGETFEAEMTGTIKIDDNKYVYVFPPKEDKRSFQHQFAIKA

FSFNYGWGDYSQFISAGPCRHDEDGGIVTHNPIVISTSDNPDDGGVLKQMCHPVILYGIR

WAETAPGEVRTSACPLVLGEGVMHYKCLSNGMWDPNGPETDKCLEKSSTTKPTTTTTTTT

TTSLPPTISSKPIFAPIRYNKDVIDKIHILVRPGNPKQEKYNLTSFLPDVTKNWTQHIEK

TKEPRQRKAMAEGFKDTMLALSDELLKDGNAWNNMSVSQQRSTAASILDSVESSGLLAAS

TLSVGASNTTRKSNVVMKVMNVGVASGQAAVLDDRSLTGSRAKVNRFLIPHSVLTQVAGH

SKGSVPVVFVVMSNISEWLTPEAITTTSTPGSNVIDARPTQEPTRRLVNTDVISVSVSKH

RVKKLVEPVVFLMEHRQMESVANPQCSFWDPERGGWSTEGCQVSESNKTHTTCQCTHLTS

FAVLMDITGATLEMSDGQRFSLDLITYIGCTISIICLLLSWVTFACFKNLDCDRNTIHKH

LVFCLMIAQIVLVVGIQQTQPPVLCSIIAGVLHFFFLASFAWMCLEGVQLYVMLIEVFES

ERSRVLYYYLFGYGVPAVIVGVSAAVFPRGYGTEFHCWLTIERGFIWSFVGPALAVLLVN

FVMLGIAIFTMVRHSAMSHTMRQKSVPQKMSVSGSIYSVEVQEALHHRPEIPTWMKGAVV

LVVLLGLTWVFGVLYLDNVYHTLIFSYIFTILNSLQGLFIFIFHCLLNEKVQKEYRRVV

>TRINITY_DN383596_c9_g3_i1

MLQSQNMSFLVDSFLMLFSQVLFFGGGWVFFLKQLFRDYEVHHSVVLLVFSVTFSLSCTM

FELIIFEILGVMDSSSRFFHWKLGLYAILFVLIAVLPFYIAFYVIGTIRLVPQKQHVRVL

IAFGAWCAILYLFWRIGDPFPILSPKHGIFSIEQCIGRVGVIGVTLMAILSGFGAVNCPY

TYLSYFVRHVTEADIQGIEKRLLQTLEMIIVKKKRVAMAKRESLRQASLSKSSGGIWSML

KNVASGSNTENVSHLKQETGALEELSRQLFLETVDLQEAMDRIEYSKTFKGKYFNVLGYF

FSTYCVWKIFMATINIIFDRVGKVDPVTRGIEIAIKYFGIQFDVKFWSQQISFLLVGIII

VSSIRGFLITLTRFFYAIASSKFANVIVLCLAQIMVRTHCFVPQVAFYTQDTEI*

>TRINITY_DN116305_c1_g2_i8

VTVLLPVAFILGVFGNVMTIVILRRFQANRSALDRYFLCLAVTDLCVNITGPLLDSIWVL

TRFGLYNIHDVMCKLRYFIQKTAGSCSAWFVVVMVTHRAMSVVWPHRVNVLCTAKKSWGI

IVSIIIFFSGLYCNILYGMEVLNGICLPKKDYLVFVSEVLTKVSTVLTSLLPFTCLMICN

IVLVVKLRASVKEAGDQFAATDRQQSERERKVNSITLTALVVSVSFIVFTLPLAIKNIIS

LTSVYSSQVVDFHKLALHYFQYHLVLCIYYLNYSMNFYMYCLTGERFRNNFLKLLHIVNL

KS*

>TRINITY_DN383687_c0_g1_i1

MQSRWLLPLLVVLAFTQLGSSLVPATCPNLQTNTCYFTNVAKLQPWANTQYYAVGGLFDI

HERGTNAFSCGSKINPYGVLLSQAFLWSLDYWQRQTNSNVSVGAVVFDSCDREDQMIQNL

LGFEQCNIRIQDIDRHNLVGFVGTSNMDAMAAASLTADMDLTLISPMANSISLSNHKEYP

YFLRTVPSLQTDVDIMAAVLDHVETKYVAAIYQESEMATFKAFKMKMEAGSICLTANFSM

MGDESNDTIQNFVATSLASVEKTRFVALFLTQEFAVRFMKQVTAVEKVKNMNIVFLMTSS

ILPTSALFDGTNAVAASDAIVLERSQQTSVQNAVVEFKQYWDQLVQKTALPNSRTSSPFL

TQYLNSITSSMQAPTMHWALNNTIMATKAFVEGTRQAGVELCSNTNSYLCSSFFDVRSRG

SNINQKIRSKSNYFTPTTADGKFQNGDLNANFISYKVLRYQQNGDFMQVATYSNSDLSFS

VPIKSLGPYRPGQCQDSPCLQCGELPTTTTTTTQSTTTDAQPCKPENAGNF

>TRINITY_DN383907_c1_g10_i2

RYFLCLAVTDLCVNVVGPLQIWLILITKFKLLAIHDVMCKTNYFIVNTAFACSKGFVVVM

VTHRAMSVVWPHRVNVVCTAKTSWGIIVGIVIFFSMFYSHLFYGMEVSGGNCGPKIYYVD

FVTDVFIKIDLFLSSLLPYICLMISNIVLVMKLKTSVKEAGDQFAATDRQQSERERKVNS

ITLTALVVSVFFMVSTLPLHIVVTVINYNPKVDGYEREILKFMLSLTGSLVYLNYSMNFY

LYCLTGRRFRKEFLKLVSGL

>TRINITY_DN383907_c1_g13_i1

MNMTNITRWIMTVSSITMGESTSSPNITDYNLIQINKWIDFTYSVSILILGLFGNMMIII

IIRRFQSNCSSLDQYFLCLAVSDLCYLITGPLPNCIVISTRFRWIHAHDVSCKTGFFIMN

MARACSVWFLVAMVTHRAMSVVWPHRVNVVCTAKTLWAI

>TRINITY_DN116305_c1_g6_i1

ILGVFGNVMTVVIVRRFKDNRSALDSYFLCLAVTDLCVNFTGPFPDWITTMGNFDVFAIH

DFVCKMDFFILNTASASSSWIVVVMVTHRAMSVVWPHRVNVICTAKTSWAIIVSVVVILA

LAWSHTIFGMEVSNGACILKEEFMVFVVSVWTKIDILLSSVLPFICLIISNIVLVVKLRT

SVKEAGDQFAATDRQQNVRERKVNSITLTALVVSVSFIVF

>TRINITY_DN451059_c0_g2_i1

MSVDVTTVVDFLTSTAASIIDTGSEISTGSGSIGDGHAHVERKHDDKMAQMLTTAQRMHY

AASAVVGPIFVALGMLGNILSIVVWSRRNMRSSTGRYLTALAVADSGVLLWFILTDTVKM

MHPEVVNSTAYAVFFAYLGYPFFFLWVICSIWFMVGVTVDRFIMVCLITKAKEYCNEKRA

NVGIGLIGGLCFLINVPHFWSFTVDWDKGVNATGPALLKTEFQKGEGGMSYELWVHCIFL

VLVPWFTVFTLNLLIISKIGKTNRKMSSTKTAQSADKSRQSENQITRLLLIVTFTFLVLL

GFQCITQCFYMVMPDGFDKTIIDESFAVAKLGVIINSSINFFLYCVSGRRFRQELLALLG

LKRRTLGLFGSSDHSSSTGTRSGTGTTGV*

>TRINITY_DN502148_c0_g1_i1

MDEKEQLKHLAQARYACWLQVKHYFNVSQYADPAVVQCPVAWDNILCWSEAPPGTVQSQP

CPDYIHGFDTHKNATRECAEDGTWYFSPLTNRTWTNYSACFDPALQPEVKDIAEFAAHAD

SLKLLYTVGYGISLGSLIIAVFIMCCCRRLKSKSNTLHVNLFLAFILRAAVSFAKEALFV

QHLGFEKDVRRMPDGRLEFIHNGTHWECRTLISLFIYAICVSQMWIFMEGLYLHMLIYST

LSTERRGVRLYVLMGWLSPLLFFIPWVIAKSTTDNHFCWTISTKPNLFWILNGPMMATVI

VNFVFFLNILRVLVSRVRSADRHTSRQQQYRRLAKFILVLVPLFGVMYIVIYVVFPMSFS

QLDIKHLYIEMAYNSFQGFILALLFCFLNEEVHAEIRRLWMRKRNMRRDSMALTRSFALS

SYRKQSIPAGKSQGSVVRTTQNNNHPTTNTITTTPSFRLTRTC

>TRINITY_DN532601_c0_g1_i1

MSAMVSEGNGTNGTEGDDGSRPPRMVFLALIMLVSCVVNVLLLYAFAQNKKARQNVQSIT

VINLAAVCLGDCVLNMTLVMGSLSAGSDWTFSEELCRWHSFVMSLVLVETTLLLTVLVCD

RFLAVKYLEKYDSMISSVRLIIVLVFTWIQSLAFSIPFFFNTVKTTFQQKLHTCSVSDET

SLAFICIASVLCFLAPALVIIVVFILIFHASHKQQHEVKIIVTQNHYCDQVKQEPALWKE

MYKVKYVAVLCLLWFILEIPYIVTTYIQQYRRSSELNFSVDYPWGVDVTFIWMRFSFSAL

VPCITFIWRKDLWHCCKECLICHRSNSVLDIRVTVDPTTAKTTRNRTKNVPPSPKVPAKS

KDQPKGEVPNLLSFNVPVLFATSDGIHIETPYSDASDTEDGDADCRDHMPKGRQLDITGQ

SVDHRLPADTSDYDSEAEVYSSQPLSTRHIRGALETNSLPD

>TRINITY_DN57099_c0_g1_i1

DRGIQFYPLVKQPAYMIAIFSVAYGMVLMLALLGNACVLAVVIKDKRFHSATYVFIANLA

VADLLVALFCNPITLLTNIFNGWRFGAFMCKAAPYLQGVSVCASVNTLAAIAVDRYLAIC

HVLRIRMTMRMARIILGAVWLVATTIMVPWAVFYQQQTFELPNQSIPVCVQAWPDPKQPG

EFFLGAVFLFCYALPLSFIVCCRS

>TRINITY_DN88036_c0_g1_i1

KKKKRKKVSTSSSAVMHSWTWNNSTTSSSNNTTARNGTHSCRVSYKKYWDNDNTDTAVGK

VCGMSVGMANVSGVRGKRNCCACADVAVYMAYTGNMYGHTYVNKGVSVYGVYVSGSTVAR

CCTCRAKKKTRTAGMVAVSSVVGVHVAGKYRVDCTYKGTKGSYSSYDHMVDNGVVYGATT

TTTVTAVKKDAAVWRGASATVSKAVTRMVAVSCTCTNVMRTAM

>TRINITY_DN111878_c1_g5_i1

YCGVTNNAVVRGKDRMNCWAMADSVTASCSAAVNTYARYVNAYYTVATSVHTSGCTAVAR

CVCVVKASSTRTMATTSSVVSTDKNTGKTTWSGSWRYDVVVTTMSTVTCTTAWRRMATAW

RTTSSSNASNVVTTKMSCVVCSGCASMRRDVSTYGYSNHNSVAHTNYTSGTNSSVNCVYY

RS

>TRINITY_DN110801_c0_g1_i4

MVTMTNVSIGYMTTVIEKSHPFIPWDNPDNIISAATADKLRGVISDCTKVVLLLGIPGNI

VNLVVFTRQGLKDKVTLMLFCLALVDLLNLLCFFSLNIIFFIIRLIDPLLAWNVLTLQYT

HVLYINMMAAILSVTMVTILSVDRCLVISLPLKAARILTYRRTMVTIVVVSVIIIGVCIP

SLWLNTVTWVTDPVSNRSKAQLIATEFTRQHPLYRQFYGIFRIAIKMVCLVAEVISCSIT

VTTLRAASRVRAKMSETKLQKSDTQITVMLLFVCMVVVMCLLPEQVVTIVFLVVPGFFMF

RKYHNMFFVCQYIVFFTQCFNSTSNFFLYVLLSSKFRATLLELLPCRKKMR*

>TRINITY_DN113643_c2_g1_i1

MTNSTGNDVDSTDSHTTNVDMWNSDVSAHTVYVMTTDYMGGNVCCVMKGSDRNSAVADNV

VSDCYWDKVASNWTMNTKVYGVWASVSGGVMSVDRCSVVMKAGRSYRMAAVMTYGVVHVT

VAWRVDTNHSVAYVTTDWDSNRSTGMKVAVCCTTKRASRKRRMSAKSGRGDNRTMMCVVY

VVDVGATYSYVYKYHNTVVYGVGSSNSTVNAYVSSKRTTRDMCCCGKSSRGWHKD

>TRINITY_DN113643_c2_g3_i2

MTNSTGNDVDSTDSHTTNVDMWNSDVSAHTVYVMTTDYMGGNVCCVMKGSDRNWAVADTA

VYANDCYMTWDVTTNWTMMSCTTYCWTSVSGGVMSVDRCSVTMKAGRTYRMAAVMTYAHM

NYTVNWRSDMTNRSVAYATTGWDSRVMTTGMVKVVVVVTCCTKRASRKRMGNSDGRDNRT

MCVVYVVYVGATTYV

>TRINITY_DN113676_c0_g7_i2

AVHSVTMTNSTGNDVDSTDSHTTNVDMWNSDVSAHTVYVMTTDYMGGNVCCVMKGSDRNS

AVADNVVSDCYWDKVASNWTMNTKVYGVWASVSGGVMSVDRCSVVMKAGRSYRMAAVMTY

GVVHVTVAWRVDTNHSVAYVTTDWDSNRSTGMKVAVCCTTKRASRKRRMSAKSGRGDNRT

MMCVVYVVDVGATYSYVYKYHNTVVYAVTGSSNSAVNAYVSSKTTRCHC

>TRINITY_DN115321_c2_g6_i3

SNHTVVNNSGCTVRNKYWDNDNVSATARRNNVGAANNMAVYKGKDRVNCASTDGYTVMVN

GHMTHDYGVRTATNHNTGGMWSVSAATRCCSKTTKTMAVTVYVGGVRYRGCVYDSGSVSY

HVGYKAHKDTYTVYGAGGVVVVTTTTSVKRATWRAGTSSSSARVATKMGNSACTVGRVVC

NVGGRHNYMSGWVASNSTNCVYYTMGSRYRTWASRHSKNKT

>TRINITY_DN130479_c0_g1_i1

SNCHAPVNEISSCDDLLRSTIYRVFLSVFATLSLTGNIVSLMTRILLQRSNRKSGYSVFV

THLCISDCLMGVYLVIVGIADRLYQNNYLWEDIKWRNSAVCQMAGFLALLSNEMSAFVIF

LITVDRFLVIRFPFSHLHFLPKSAHLICCIHWIVAIILASIPFFPNNTHWKFYSQTNICI

PLPITRSTFAGHNYVFGVMIVLNMIIFIAVAVGQTAIYL

>TRINITY_DN84676_c0_g1_i1

YSVFVTHLCISDCLMGVYLVIVGIADRLYQNNYLWEDIKWRNSAVCQMAGFLALLSNEMS

AFVIFLITVDRFLVIRFPFSHLHFLPKSAHLICCIHWIVAIILASIPFFPNNTHWKFYSQ

TNICIPLPITRSTFAGHNYVFGVMIVLNMIIFIAVAVGQTAIYLAIKSSSMALADFNRKR

QDMNIARRLITVAATDFLCWFPIGLLGCLASTGTAVPGEVNVTMATLVMPVNSAMNPFHY

TMSVVLEKRRQAKEQEILKYLKAQSVKS*

>TRINITY_DN337635_c4_g1_i1

MNVKFWCTIGTLLSVASFIEGRWVEDTIETNEDWAFIARFCFLSKQGALLYSFLYPVSYG

TQEILLYYDEPGQWESVYDSNKNCSTRRSVLSIANNQIIALNTSYTKTSRYSGCSLTTID

SQQYYNCSGGRTFRSMRERWWYIAVARCDDAFGSVTGLNLQYKLHMTNGEPDDLWHYEYS

ADEFYILAENIGFLVVYLVMVVLSAVCAYILKGRQLFHSTYKMYMVAISLWFLGLLLMSI

AWGQYGGSGWQKKSTEVTGRLLQAASNVIFILMLILMAKGYSITRGRLPHMSTVRIIVFL

VLFIVVYITLFIWEGLFFDEGLVLYFYESPPGYGLVTMRLIGWLWFLYAIFFTLKHHPKK

GNFYFPFFIFYTVWFWAGPVVVLVAMFAMAKWSREKTVFGVEQFVGLCGHLFFLILTRPS

AANKNFPYHVRTSQIASLIDPNEDDSPRSGVSTYRMNKDEEPDGAMPYRTEETQLERGPD

LAALFVVQDTRGTPRGAGNVGVPLGPLCPPAYTPLTLPPIRSQAPTVTLPPLQGSTLPPI

RGLSLPPLPVEPTAPPLQNNSTHTEAGPPPSYDSMFMAKSQ*

>TRINITY_DN115848_c1_g3_i1

MSGVTLSQTTEFSPLSQNTQSTHLVFLESSASPEVSHDDDDGDVLLSADVYWRAVPAMDQ

MLCALQVIGLVLNTANIIVMTRRSMRSPTTTFLVALSVLQLVYITVGLVPAVHRIFKPLV

ITDAFYLINSIYVINYGMTCLRRSMYCIQCCVSVERLLAVWLPLKAKQFLLVRRPWLFIL

ATPVVVFLTHIHITLKLEVFETTSPQNQTIHSFRYTQHYRHHSELFDSLSITMKTIFVYL

TLVMLITTNLAMTGVIKRYASMRRQMKTNVDVDAAQKRETQMTLTILVSTFIFVLLCLPT

VTNSLAYNAAPKSYGPFSLNRYLFLFMQKFGGLLFTLAFSTDFFTYVALSSAYRNTLFCM

LKIKKEEMLISTRSGSKTKTSSCP*

>TRINITY_DN375995_c7_g1_i3

MEFTSSTDNNHNHHLHLQPYPHNGHLNVSENHTDLDPHHPHQHHLLLLPTTTTTSNSADD

PAGLSDSLVTFQLWFQGFHGYASIVVCIFGIATNFFNVTILMRKDMRTPTNILLMWLAVS

DILTMMPYIPFVGNFYCPPTTPFSHPERFSYGWMMYMLMMINFVATTHTISIWIGVSLAA

VRFVQMRSTSRGHVANERRIRQAKIVTLLVYLLSCLVMVPNYLTNKLDLVQMGNITFYTI

QDLKLATNQTRSIVLINVVTYAVVAKIIPCVLILIFSGSLVYTLTVKGRGRRRRLATSSC

SKTSARARQATTTRMLLVVIILFIITEFPQGVLILLSATLP

>TRINITY_DN57958_c1_g1_i1

MAAKAAITLLTTTSISTSPANNPMVNSTTMNGTMKSSRWMKRNVPFWDAAFLTVKITGSL

ESILIIAVNLTLLSVILSSPLLRSRMRNHLVISVVIANLIVGILSSPFAVDATVRRKWVH

GCYLYVLLILLTVYVQNFVSVWGIVALLLHYLARLLRYEGPNWLGRLPASVQKAVPGLLI

ASPWIVSMVLLVPLVFGGLHKFVWAVWTYTTCPMVLQEWATFLLNCLSFFIPAVILVVLT

VLIIILHRRRSDKTDSQSAMETGMKVVPDVGQELETCWVHVLVAVLTILMMGPEHSFLMS

RYQIRGPLKSIVIASLSVHLLSDLTPLAVALIWLLMLPDVRGRLLELLSKLPCNFHWWRG

RSPPDASGNTSIAPVAFRDLHDE*

>TRINITY_DN29158_c0_g1_i1

MAATDVLLQQAVDLRKTNTIHASEILTKIVKQEVRTLDDEAIRVKEQAILEIGNLFAETK

QATELAGLIKFVRPFLGMVSKAKAAKLVRSLVDLFLDMEAGTGKEIELCKECIGWAKDEN

RTFLRQALEARLISLYFDTQNYQEALALGSILLRELKKLDDKALLVEVQLLESKVYHALG

NLPKARAALTSGRTTANGIYCPPKLQASLDMQSGVLHAADERDFKTAYSYFYEAFEGYDS

IDSPKALTALKYMLLCKIMLNVADEVQAIVSGKLALKYTGPEVEAMKSIAQASHKRSLAD

FQKTLGTYKSQLAEDPIVNAHLKTLYDNLLEQNLCRIIEPFSKVQVQHVANLIKLPVDTV

EKKLSQMILDKKFHGILDQGAGVLIIFEETVVDKTYANALETIHSMGKVVDALYAKAKKL

T*

>TRINITY_DN110890_c0_g1_i1

YMLLHSTENLYTQILGRFDPYGPVMTFKTNNGLNVLYGFVYASGFITTAIAFERCLCIGM

PFMAKKVLKTKTAATIVVVVVAVLVGMHYIVAEKFKIGCTYYPKMGFTLKGYYPSEFYMK

NSKIIDALSSAVYGITLPCFYAATTIITTTITVIKLKTAVTWRQQTSSLKREEKSKELAL

TRMLVAVSCLFIACTIPNIMVRIVPLPFPDFQLGGRYQNLFLCGVC

>TRINITY_DN108432_c0_g1_i2

MYDSMWNRSVNSSDGDNVTVASVSHRGSDATARVTVVVGMGMAGVMNMSMAVRGSDRNCS

ASDTGYTSTRHSTRDVADYWRCSVGYVGTTSNCTSRCVSKARTMTRHMAVVMTSCCAVYC

KVKTDATSVAYKAVYSKDNKDAYTVVTACGVVATCAVRMHSMAWRSAHTTSDRKASVTRM

ACVMYTVCTSVRVVAVDGKRYNTTMSVHTAVNSCNVYVM

>TRINITY_DN111878_c1_g5_i4

ARSNMSSATSNSNNTHVTNHHSTANWDNWNRTRVAATYYCVVGSANNCVVYRGKDRMNCWS

MVDCYTWVVGTVRTDWSSNYVVKAYYTGCAATSGCVTAVARCVCVVKASSTRTMATTSSVV

STDKNTGKTTWSGSWRYDVVVTTMSTVTCTTAWRRMATAWRTTSSSNASNVVTTKMSCCSR

CTSARRGKVYNYHAAHVGYHGAANSSVNCVYYSRSSKYRTKVACRKATNYSSNTGDSKS

>TRINITY_DN111878_c2_g2_i3

NCVVYRGKDRMNCWAMDMGCTTVASVAVDWYAMTYRVAVYSMTGGNAVSSGCVTAVAMRCV

CVVKASSTRTMATTSSWKYRRVDKGKTWSARNDYSHVATMSVVCTTAWRRMATAWRTTSSS

NASNVVATKMSCCSRCTSARRGKVYNYHAAHVGYHGAANSSVNCVYYSRSSKYRTKVACRK

ATNYSSNTGDSKS>TRINITY_DN195636_c0_g1_i1

MDIFTSALLFFIVALVVYLFTTRQKHSKPLPPGPRGWELVRAFPSTVNGKLVLEAEKWAC

KYGEVVFLPLPQGNLVFLNSPSVAKELFAGKATEKFTNNRMWSYVGEKVCLWKCVAFSNY

SDPSWTKMRKLLHSNLKFYGDGVAKFEETVGKAVDQFVTTLDSYCGQDIQFKDLVEDSLV

GIISVLLTGQAPDKARIQTMKRLSLCALQVDDPGFSFALKVCPLLEYIPGTYYSMVRCEI

QQSKAEIVKCFFEDIKKTRIPGSSRGVVDNLLEYQSNGGADWMTDDHVIGMILDIVVAGS

ATTTHTMNAIVFYLLHHQDVTHKIQEEVDRVIGHDRKPTLGDRHSCSYTEAFILESMRVL

SLAPLGLDHLVIQDVELKGFHIPKGTVAFNNTF

>TRINITY_DN223056_c1_g1_i1

DKGQLCGELRAAYGDIFSFRLGCRFVVVVNGFESLKNIFVHLGGSFVDRPQIFTFTHVGQ

GKGIVHSSGRVWKEHRKFALNTLRNLGIGKSSFEDKIHEEVRAFCKVLDDTEGADFDPGC

AIQTAIANIVCSIAFGKRFQYDDPLFVRFLEIFNENMGLAGGTALLNFFPFLHRLPGDLF

MSHKMLKNVDFVQSYLRKWLDNHQNAFDPDNISDFIDAYLNEIIKRQAKKPRTTFCYDQL

LKTVGDLLVGGTETTGTTLRWFLIFLVTWPHVQTKMRQEIDSIYPARTTPSVHDRRRLPY

VEATIMECQRFADIAPFSVTHAASEDVEVEDMLIPKGTIIIPNLHSVHQDPDLWGDPHLF

RPERFLDDKGEVIRPDYLIPFFLGKRSCIGEGLAKIELFQFITTLLQRYEILPSIQGTLP

SLEGHLGITYTPCPYNVRFVKREPLSGCVLPYEAMNSV

>TRINITY_DN240182_c0_g2_i2

GARNTLTAGGVSALSFICPVLRYLPGDQFQIKKKATLYGQLRDDFVQPIINRHVQQREEQ

GQGQEGEADNFISGYLDVIRRPENERSKEYVNVHNLQRVVLELFGGATDTTSTALLWGLA

YLLHHPSVQERCQEEIERVVGTHRPPSIQDKARLPYVEATIMETLRFSGTSPLAFPHSVP

CDVNFQGYVIPKGTFVLVYLDTALRDPEIWGDTADQFCPGRFLDEEGELKKAEELIPFGA

GKRKCLGENLARTELFLYLSTLLQRFRFLPQDDCHLPSLEGIMGITRCPHP

>TRINITY_DN241360_c0_g1_i1

MSRVSKALQRLVGGSVQLPKGQRAVSTSARVENSLSLDLDVNVKQCPFRATMESLSLGRG

GPEPVPVPEASEGSTVRSFEAVPGPKRLPLVGSILDFFKKDGLKFSKMFEAYQARSLEFG

PIYREQLGPVEMVVISDPVEYSKVMRAEGKYPNRLPMEPLAYYRQQKGIDLGIVSSQGEE

WHRQRTAVSKHMLKLQAVAQFCQPMDAVSQQFSQRLHDIRDEQGEVQGLEQEIFKWAMES

IGVFLFEDRIGCLESKPSQQAQDFIYHLRNAFRLMQPLLYNMPIYKLFKTSRWRKFEYHS

DQVMNIGQTFVDKKMEALKRGEENNTSAFLSDLLANPSLSSKDVTGLAVDLLTAAVETTS

TAMTWCLYCLAKNPEAQNRVLSEVNAAVPVGGEITADALAQMPYIKAVLKETFRMYPITY

ATSRVVPTDIEVLGYNIPAGSHVQANLFGMFRDASLFPEPETFQPSRWLRESNMDPKLKS

LSSLIWGHGARMCIGRRFAEQEIYLLLAKLVQRFQLSFHKEDVEPMLNTVMTPDRPVVIK

FTPRQ*

>TRINITY_DN242637_c0_g1_i1

LRRDEPLYTWFQHLSQLHGPLITVPAGRETVVVVCQQSLTEELWKQSDAFCPSSWLAWAS

KGRRAHRKPLLPDTSCLYNDVITILTQRIQLKVFEECVTSSLDVIIARIRNLHGVAFDPQ

ALMTRLAFDVTFSLFFGRKLPADKYQQFMRAHTQLNEAAEGAVVAQFSKCKDISPATFSK

MRALYDQLCAIIAAELETPAANAQSRTGCGVVREVLKAQASQGTLDEMQTHYSCDVSALT

PALLDIFTDWVDTCRSSLCWSLWYLSHMPEVHSRCQREIDTHLEDHAPGVDDEDCLPFTR

AVLREVRRLRPSQPLSEPWAVTQPASLGGYDVEVGMMVVVNQWSVCRDAYTWSRPTDFTP

DRHLQTPGRPPLFPSRTPTLTSHPRCRSEDKVLTLQILLMTTSLLHNF

>TRINITY_DN249182_c0_g1_i1

MKSPSNEDESTTATSSTFLGLEASTLVVLAALLLATIYVILEARTRRLYPPGPRGYPVLG

YLDVLWSADRRKLFKELRTKYGDVFSFPLGSRVVVVVSGQDKLNQVFVQQGSSFLDRPNI

FTIAYTGQGQGSSRAAKANSSTQIWKEHREFTINTLKNLTIGKHAFQDKIQEELEAFCNV

LDSTKGVDTDPAHAIETAVANIVCAITFGKRFGYDDPIFARFVTIFEESVGLSNGSILLS

FFPFLRYLPGDLFKSQKVVKNAEHIQGCLRQWLDAAKRRFNPLDVGDFMDAYFSAMEEEK

KKRSQSPPSSLSHDRLLKLVGDLLVSGTENTATTLRWLLVFLVRWPHVQRKLRNEIDIVY

PEKTTPSVLDRGCMPYVEATILECQRFADIAPFSLMHAAVEGVEVEGHHVPKGSIIIPNL

HSLHFDADQWGDPENFVPERFLDGDGELLRRDHFLPFSVGQRSCPAEGLARLELFLFLTT

LIQHYEIRPASQGKLPSLDGRIGLTYSPCPYKVRFVRRDQPEEEENDAETVEGEEAKSSA

KDK*

>TRINITY_DN24966_c0_g2_i1

TSKFEVTAAETAKISHNVSAHVSCNYGTKSNARPSRIDPQLEAPSSSKRGNVCRTIAKSN

SAPKPFQEVPCSSDRPFSLMTLLRMAWYSARLNDWFLHNFHSLGPVWRLRAGHFDLVNVM

DPHSVEAVYRQEGRYPQRNRLPLWEHFRHQEDESFGVFLMTGKDWKRTRQVLNKPLLRQG

GVEFHVSELNRVVTQFLQALSSHTTHTGLVTGLQDLLFLWSLESVGHVLYEKELGCFRQP

FDRQAWDFIHSLHDVFDTNNALIALPPALARVIHPLQWRRHMNGWKVCFRIARHAIDEKM

SKLKRTDQRQTAPDGLKGLKATAEDKGLESTANNEGFLAYLLSQSHVLSQKEVIGNVSDM

MMAAVDTTSNTAQMLLYELAHNAQAQS

>TRINITY_DN266959_c0_g1_i1

MLELLSGLCGCGCLTSLGLLGLLVFLAYLYGTWTFSTWTQLGIPGPKPQPFIGNTREFRK

MGGFKASLYWTKKYGKTFGIYFMRQPMLMTSDLDILKEILIKDFNNFSYRPVDNPLTPYP

LNLGLVYVNGEQWRRLRHTMTPTFSSSKLKMMGRFINRCCQNLAGSIEKSSEAGEVLDIK

HLFGAFTLDVIAGTGFGFETNSLTDEESVFLKHAKEVFGKSNPFQVVAVLVILFPALAPL

LRAMGFRTNPKKDVDFLTDTIRTVIEQRKQGDKSGQDFIQLMLDAEASDSEVAREPTKKH

LTKDELIGQGLLFFVAGYDTTSTTLQFLSYLLAMNPDKQD

>TRINITY_DN295583_c0_g1_i1

MDALMIVLLEWLTPVLLFLLTAGLFYLYVMWPYSTWSRLGVPGPKPSPFLGNAKDFNEKG

FHAYMEWTKKYGKNVGIYMTRTPVLLTADLEVMKEILVKDFQNFSNRGLSSNDLLPPVFT

NNLINSKDGAWRRQRHTLLPTFSAKKLKMMGRFITYCCHNLVKSIEMSTDAGTLLDVKTL

FGSYTLDVIAGTGFGIETNSILEGGNEFLDNVKVIFNSFQLGTLPIAIAATLPALAPVMK

KLGFYSMKMKETNFVTDAVRSIIAQKKQSGQTSEDMMQLMLDAEANESEVNNSTNQKVLS

RDEIVAEGLVFFIAGYDTTATTLQFLSYLLARNPEKQEKLYREIVAAIGDAEPTYDNVME

IAYLDCCIRETLRLFPPGPAFDRFCSNTRTINGITIPAGACILINVASLMKDETYFPNPE

EFIPERFQDETRPIPALARDMMFGAGPRHCIGMRLALFEAKMAAVSVFRKFRFVKVKETV

DKIIPKPGGRINVSMTPILIGSERR*

>TRINITY_DN297971_c0_g1_i1

VAGYDTTSTTLQFLSYLLAMNPDKQDRLYEEIVTAIGDAEPTYENVMSIAYLEGCILETL

RLYPPISVLMRKCVKERTIEGVTIPAGSTVMASVIGIMRDEEFFPEPDAYLPERYQDDSH

PIPQLVKELVFGAGPRQCIGMRLALYETKMAMITVLRKFRFVRVEETPAEITLAPIGLTA

SSVPLYIGAKRR*

>TRINITY_DN299683_c0_g1_i1

SFHLEPGRWGTAGTPITCCSSVLGNFVSLNGPDMVREVLAGRHTEDFVNDRPDTFIGPFL

FYGPKDVAMASISPSWGAQRKMFHSSLRLYGDGVHRFESTVQGELSRLIEELEEIEGQDI

CIEDYMSNTLLSILSILMTGERPGKESEVPVAMRSLDRVVSIMGTPSVDILLQLFPFVRH

LPGWFKTQCSQAVYYRNKLVDTLFNYSKSTVVEGEPRGIIDVMVLAQQSGSFSVTDDHVK

GLILDIVVGGYVTSLTSFLSTALVLLNHPHIARAIQAEIDTLVGQRIPTLDDRKNFHYTE

ATLLEVLRFMRFFPMGLPHICHKEIQIGPYRIPKHSMIFANQWECGRDPNTWDHADRFMP

ERFLDSKGHLLPADHPTRKKSVSSYNIQRH

>TRINITY_DN310370_c0_g1_i3

METGVCLSVTGWLVLVLVFLFTWWFWTTRRLFQTIEKMGLPGPPPSFLVGNMKFITSKGF

AASFKEWEKQYGPVYGVYRAKRPSIVIHDPDMLREIMVKQFDTFPNRLRFQISDRKPLCN

MLQFMVDDHWRHVRKTLSPSFSSGKLKSMLPVIQRACRHLQERVQQMAQSKEDVDVKVLS

NAFAMDVTAGTGFGLEVNCLQNPNEPFGTIAKRILYPPQWKFTLLFLFPAFTNVFSALGL

DIFAPRDTGYMFDVIRTALKERRQDKQQKYSDHLQCLVNAERGGRLEETNPYPLDPELDH

GNHLNTQSSWNRKGLTEEEIEANSLNFLLA

>TRINITY_DN311406_c0_g1_i1

MWLQGPAWSLLPFVVAILSWQAYKLFIKPFLSPLRKIPGRRYKPIVGNVLEAQKEEAMTS

TIKWMTEAKSRFIRFYFLYGEERLLAADPAIIKYICVTNSKNYHHSSGLRIMQSLAPKFL

LTLNGQPHHSLKRLLTPAFNTQAVNEFIPVFDKKTLEMVAQWEEQIAIDGAHECTVPAQS

CMAHITLDAICECGFDYQLHCIQDPDSAGVHYMKKILEGFRIRIRDIIPLISRFPSQEKR

EQDKAKEFFSSTIWKVIKDKRRKMETGTDHHSQDLLSRLMAARDEEGNTLSDDVIYSQVA

GFLFAGFETTSISLTWTLLMLAQYPDVQEKVRQEVMSLLPGQQPITAEMVQQLSYLTAVI

KETLRLFPPIPLHFRQAVSDDVIQGYTIPAGTTIGLSSGALHRLPENWPDPESFKPERFL

KEYDPYAFLPFSAGPYICIGHNFAM

>TRINITY_DN317004_c0_g2_i2

CSDLSPLGLDHLITQDVELKGFRIPKGTTAYNNTYVSHKNSDVWPDPDSFKPERFLDQDG

HLVPADHPNRQNTLTFGLGKRSCPGETFSRARIFLYVTTILQQFDIIPKVHESFSRGSSF

SSTGGRMSNCCKIVVT*

>TRINITY_DN322174_c0_g2_i1

HMRTEERLRYYLDLTAKYPKFWRVWFGPFRPSILLHHPDTVRALLRTAGPKPWIYDLFLP

WLGEGLLISKGSKWSRARRLLTPAFHFDILKPYCHVSNQSCDLLVQKVEKCASEKKSIEM

YSNVSLCTFDIILQCAMSYTNDIQTRGENHPYIHAVSTISDMNIKRSRNPLLHLDWVYAL

TPDGRKNRQLCDYVHSISDKVIKTRQQTLEKEGPPKKRYLDFLDVLLTAKDSNGQGLTPL

EIRNEVDTFLFEGHDTTASSLSWTLFALCQYPQYQKRVQQEVDTVLEGRHCHDITWDD

>TRINITY_DN329548_c0_g1_i2

MAVEIVLSCAALFFLVVFVVRNRLVKQNRKHNGKPIPKVEPCLPFFGNALHLDTTKCHLV

LSELRQKYGPVFFIRMFKEKILVLNDFASIHDALITKGSDFAGRPFMYRTSHAERNKHSI

VWQTYTSKLMFLRKSVLKCLRMYGTGLGKLEERCAPDILHMCKRLEAMEGKAFDPQNPIY

DSVCSIMLRLTLGTRFDYGSSSFQRIKEMNVLFNDTFGSGRARRLDFIPLLWFFQGDSYC

QMQKALQLRDEFWDEQLLNIKGSKVEADCIVQTLLDQTDSSTDSAATATLSSPSSSSTAT

TVNDKQQGWNQPELSISTIKEVFTNLILAVIHQMAVEIVLSCAALFFLVVFVVRNRLVKQ

NRKHNGKPIPKVEPCLPFFGNALHLDTTKCHLVLSELRQKYGPVFFIRMFKEKILVLNDF

ASIHDALITKGSDFAGRPFMYRTSHAERNKHSIVWQTYTSKLMFLRKSVLKCLRMYGTGL

GKLEERCAPDILHMCKRLEAMEGKAFDPQNPIYDSVCSIMLRLTLGTRFDYGSSSFQRIK

EMNVLFNDTFGSGRARRLDFIPL

>TRINITY_DN329548_c0_g1_i3

GRARRLDFIPLLWFFQGDSYCQMQKALQLRDEFWDEQLLNIKGSKVEADCIVQTLLDQTD

SSTDSAATATLSSPSSSSTATTVNDKQQGWNQPELSISTIKEVFTNLILAGTDTTATALT

CFLLVLLHHPHIQQRLQQEVDTVVGSARSPSLADRPLLPYVEAVLMELLRYISHVPLAVP

HFTMCDTSILGSWVPADTTVYINLWSLHHDEAEWTDPWQFDPSRFLDDKGQLIPPSHKNR

RKLLPFGAGRRVCLGEVLAKNRLFLFVTSLIQQFHFEPEDSNNLPDVDPRSYEMGLVLHP

KPFRLCAIPRNPSDCAEGRKLRPHAIGI*

>TRINITY_DN331664_c0_g1_i2

MIIGRCYMVLPISSMRCVRDGHGFAYRARQLAYCQCRRHLADTTRLLSSVASSTSGDIKP

FEDIPGPKGLPYFGTYFKYKLGAIDVFKLFDTIKEWHGQYGSVVRETCLGRTMIHLSDPE

DIRTVYAHEGKLPRIDPLAGPTLYYRRKKGISLGLGNTNDEEWYRVRSAVQQLMMRPKQV

VPFLFAADQVAQDFISHLKGLRDANMEVPGFATELARWSLESFGVHCFNTRLGYLTEAKR

REVEKIVEANNTLFDVLTRLYAAFPVYQYTHIITPKWKRLFEAEDFLYDVFLQKFNTAMG

QLREKTESGDLKEGDFLFLRYLLSREELSLSDMAIITHSLLYDPLPTVRPQVQFLLYLLA

VNPGVQERLYEEIQSVAPPSHSPLT

>TRINITY_DN331664_c0_g1_i4

TEAKRREVEKIVEANNTLFDVLTRLYAAFPVYQYTHIITPKWKRLFEAEDFLYDVFLQKF

NTAMGQLREKTESGDLKEGDFLFLRYLLSREELSLSDMAIITHSLLYDPLPTVRPQVQFL

LYLLAVNPGVQERLYEEIQSVAPPSHSPLTADAVYRMHYLKACFKESVRMMPMINENKRI

IRKDMVLGGYHIPADTLVLLNNYVLYRHPDYIQDPEVFMPERWMREEAEQKIDPFIVQPF

GHGARMCIGRRFAEQDIAVLLTRLLQNFHLEWRHAPMGVKYMTLNLPDCPTQYTFRDR*

>TRINITY_DN336488_c0_g1_i1

MSLQEQFDTTTLLVGGVLSLSLLWWLSTRRPAGLPPGPGPALPLLGHIHLMEKDPRAKFH

KWRRQYGDIFSLYMGTRPVVVFNGFPVIREAFVKFADVFSDRPHMFTTDRISENKGLILT

SGAAWKEQRKISLEILREFGMGKNMLAEKIQEEITHFIRAIEEHQGAPAALHRMTTISVS

NNICSIIFGKRFEYDDPVFTKYLESVEDNIKHLGGAAIINFFPFLEHIPGDIFGAQKILS

NVKHVESTFIQPQLDIHTKTLTGETADYIYAYLREVKKREKQEGDTTLTEPHLLKTVGDL

FVAGTETTSTAISWTLLLFLHHPHVQDKCYKEICDVIGTDRLPNMRDRANMVYLEATINE

VLRWVDIAPFSVQHATPHDVSFRGYVIPKDAIILPFMDSVLHDPEVWGDPENFRPERFIG

PDGKLSRPDEFIPFGIGRRMCLGEALARMELFLYLSTLIQLF

>TRINITY_DN338741_c0_g1_i2

KRVQQEVDTVLEGRHCHDITWDDLPKLEYMTMVIKEAMRLHCPVPGFSRHLTEPLTIEGV

RLPEGTTCTVNTYNLHHNPDLWPQPDVFDPDRFLPENTKDRDNYAFIPFSAGPRNCIGQH

FAMNEQKVMLARLLRRFTFSLDPSHKVEKNEAGIMRTENGMKMFAVARTPTV*

>TRINITY_DN341250_c1_g1_i1

MTSYGSFSLAALLYRLVQGVAERWTLGSSVLMVFAVLTLFTYLWVTHRWTSSFERMRDIT

CPLPLPPGSMGCPFFGETLEFIRKGGEFYASRLRALGPVYKTHVIGSPIVRVVGGEYMRH

VLMGEHVTVSSSWPRCVRRLMGPHSLLGMSSDEHRKIKPLMGALFTPTVLSSFVPRIQMV

VSQHLDQWCQTSAEEGQVLGFPSCQRLSLDLSLEVTLGQNMDRSQDHSLQRAMKQFAENI

FSLPVAIPGCGMWKGLKARERIVEEIRHKLDMAEGRSEADFLSIVELMQTEPTLGAQRQL

IEDNALELFFAGYSTTSSALCSSLLCMGRNPEALQKVEKELEEEGLLEDRTKELTYDVLE

RLHYVYSCNSEVLRFYPPAGAVFRTALRTLQIGGFQVPKDWVVAPSIRETQQTSALFPDA

ERFSPDRWMHMSEEQRERSRFEYLPFGVGARMCVGRRLAILIQAVFLVEVVRRCRFQLKN

PHPEMTFIPLSKPKDDLPVVFHSRLDDCSNSHSSC*

>TRINITY_DN341451_c1_g1_i3

MACNSAEKQGLPAGPKGIPFLGAALKWKGPQTNLEWTKEYGPIYSVQIGPNMLVYLNSIR

LVEEYMEQKGAALLGRPKGPAGIANGLLFGQGEPWQQNRQAFKCAMWKASMNEPYEAIIL

KEADLVIQQISQCLGTPLEIDTLLLPALSSRLVTFLVGESLDREHEEMKVLVSQMNDLEE

VDLTSKSTQMFLKLRQFRRPLQKVSGRTIPDMFKMSQAMQGLIHAWIDRRRAALKASGGT

PNPDVLLDLILTSQEYADRTEDYDKEMIQSIMDLFMGGVTSSLSALEFSILYLIHHSQVQ

LQVKTEIDKVKANGGTISWANREEFPYTQATLVEVLRLASVTPSSLPHVSTEDVMIDEQY

EIPKDVFVMAGIYSMHRDPEFYKDPEVFRPERHLNSEGKVVRPRSYRPFGVGERLCLGYH

LAEMELFLFLTKLLSVYRIRAEDPSNPPSFDALMRVVRRLKPFACILEPWET*

>TRINITY_DN347501_c0_g1_i2

VNETLTLFESACINIGNFTATPPEYPVTALSRASFIWKWLCEHTNKTMFGLGDWSLLSTG

LAVGVVSLVLVKIWQTYYYLNTFKRMGISGPRPLFMVGNTMAFRKQPFAQVFKDWENEYG

KVYGIFFPTPTLIVSDMDLIRDVLIKKFPAFSNKMNFFRLEADEATKDNLLSLTGDHWKH

VRNQLSPAFSSNKLKQMVPAIERVAKNLRKCLTDKAAKGEKIDLKDLCKTVTMDVIAGVA

FGLQVDSLHSTSDPFYQHGNSLVTTASKRFPLFLMFPLLEKVFLFLGITPFPKSDTKYLV

SVIDTALKERQTDTQAHNDFLQLLVDAQKAAGDQEQVDSEIDFRQQLRTSDTWTRKGLTR

SEMHANSIIFLFAGYDAVGNVMSMTLFALAANPECLKKAQEEVDEKIGKNFVTYTTAMSL

TYIDRCINEVMRLFMPGFLDRKVSEDVEIAGFHLTKGMRVGVPTSVIHHDPNIWPDPLKF

DPERFTAEARAARHPLAFMPFGVGPRNCIGMRLAQLEIRI

>TRINITY_DN354460_c0_g1_i2

MLWTLTFMLTDEKVLEKAKSEVTAKVGANGEVNEENMASMTYLKYCILEGIRIRSPGILT

RRVVEPVKVKDMTIPAGDLVIVSPLWAHRNPRLFPDPDKYIPERWEKADVEKNVYLDGFI

SFGGGRFQCPGRWFAMLELQLFLALFLQRFDCKLQVPVPDMCRLHLVGVPQPVGPCPVHL

KKL*

>TRINITY_DN354460_c0_g1_i6

MHDVVKGKLSGTNLRPMFFSLRQGFLDGLDNLDSGDYELHDIVRRVMYRTVLDNVFGKGT

LPTADQAKYKELEQHFLTFDDQFEYGIQLPTFFLRDWSNSRSWLLQLFTKAVQKYRQPKD

NEQKSLFQALMETVDTKCAPHYSMMLMWASLANAIPIMLWTLTFMLTDEKVLEKAKSEVT

AKVGANGEVNEENMASMTYLKYCILEGIRIRSPGILTRRVVEPVKVKDMTIPAGDLVIVS

PLWAHRNPRLFPDPDKYIPERWEKADVEKNVYLDGFISFGGGRFQCPGRWFAMLELQLFL

ALFLQRFDCKLQVPVPDMCRLHLVGVPQPVGPCPVHLKKL*

>TRINITY_DN356979_c0_g2_i1

QKGTRVLIPIYAATHDPEIYPDPNTVKPERFADGKDSLMAALPFGVGPRQCLGMRLALQE

IKTAVVYVCRQIKFVACDKTPKTLEFEPNLLLSPREPVIVRAELRT*

>TRINITY_DN357447_c1_g2_i1

MPSFFSFFCKTSMLDIILIFFTVFLALWWWSSSERITDLPPGPPHSLPLLGHLPLLRKDP

RPQFELWRRQYGDVFSFFMANRLVVVLNGYQTIREALVKNADVFSVRPSMFITDLVTHSG

IVFNSGPSWKEQRKLTLEILRSLGMGRQKFTDNILKEVHEFLEVVHGHQGRPTDFSQLIN

VSMSNNICNVVFGRRFEYDSTSFLRYLDLIRDNFRLLPPSNLLNYIPWLRFLPGDWFGAK

KVLQNVAEMERSFLTPEIQQHIDRANLSHAPHNNNFHVDIGDDDDDDDDDEHVDFISAYL

THIKRQRANGSSESSMKDDIENLLWTVSDLFVAGTDTLTNALLWTLLYLIHFPRVQDTCF

RQIRQVLGTTTPPGACHRLELPYVEATVMETLRISDIGAFGLQHGVSRETLFRGNRIPKD

AIVVAFLHTALNDASVWGDPSSFRPERFLDPDTGGLLKKEEFIPFSIGRRSCPGEGIARL

QLFLYVTSLIQRFRFLPAEEGQLPSLEGIMEFTHTPEPFLIRAVPRERED*

>TRINITY_DN357447_c1_g2_i4

MLDIILIFFTVFLALWWWSSSERITDLPPGPPHSLPLLGHLPLLRKDPRPQFELWRRQYG

DVFSFFMANRLVVVLNGYQTIREALVKNADVFSVRPSMFITDLVTHSGIVFNSGPSWKEQ

RKLTLEILRSLGMGRQKFTDNILKEVHEFLEVVHGHQGRPTDFSQLINVSMSNNICNVVF

GRRFEYDSTSFLRYLDLIRDNFRLLPPSNLLNYIPWLRFLPGDWFGAKKVLQNVAEMERS

FLTPEIQQHIDRANLSHAPHNNNFHVDIGDDDDDDDDDEHVDFISAYLTHIKRQRANGSS

ESSMKDDIENLLWTVSDLFVAGTDTLTNALLWTLLYLIHFPRVQDTCFRQIRQVLGTTTP

PGACHRLELPYVEATVMETLRISDIGAFGLQHGVSRETLFRGNRIPKDAIVVAFLHTALN

DASVWGDPSSFRPERFLDPDTGGLLKKEEFIPFSIGRRSCPGEGIARLQLFLYVTSLIQR

FRFLPAEEGQLPSLEGIMEFTHTPEPFLIRAVPRERED*

>TRINITY_DN358272_c0_g1_i1

MDTLLTILLNWIIPVTLLVLALTTFYLYVTRPYTTWSKLGVPGPKPLPLVGNGKAFNEKG

LSVFPEWSRQYGDLCGIYFGQIPVLLTTDLDIIEHVLVKDFGNFTHREVISNTLLPPLVK

DTVGFTTEDERFWRQRRTFSPFLTANKLKMMNQVVKRCCHNLVKAIETSTEKGKALYVKR

LFGAYTIDVIAGTGFGLETDTLLSDSTELADNIMVILSSLQIATVPVSLAQAFPGLIPVM

KLLGFHGIQVKEVHFVAGVIRSIMEEKRHSGKISPDILTRMLEAEIDEEQAAKNPDKKGM

TSEEIVALGTILFVAGYDTTATTLQYLSQFLALNPERQEKLYQEIVTVIGDEEPTYDTVA

NMPYLDGCLRETARLHPPAPMNDRLARKSCTIKGVTIPAGASVMVSTAAVLRDEKYFPQP

DKFIPERFQDDSHPIPKLLRDLMFGAGRRKCIGMRLALFEAKMAVASALRKFRFVEIKGT

ADEITSAPGAFVDTATTKVVIGSERR*

>TRINITY_DN358456_c0_g1_i3

MVAVSLYLAVAVIALTVAYFKFFFRTRREGEPPYVPGHLLWGNGAEFAQHAVRFLHKSQK

SLGDIFTIRLLNQYLTIITDPHCFEQFSKEKNFDFDPIQKQVNHNVFSFELKQARKMISE

AGKKVNGKYLSSGLESFADNLNTAFKTFHEQDKLDMNGNVRDGQFSQQGLRTLTANTLFN

ALFYTIFGRANQQEGPELQMEKTFNPETFHENFDIFHKFFNYLWLGVPVNVFPKACQALQ

VLCQQPVAHDMMNREGVSEYIKFSTSYMLQNGQSMQDIVGHNLVFLHVNYNTFRVAFWCV

YQLLQHQEAREAMIQELQDAIEQRRSEGDNKVGFTIEEFDKLPQLDSFLKETLRVSSGVF

MVRKVVEDTQFTMPNGQTYTVRKGDRVAMYPPAHHMDSEIFEDPETFKHDRFVDAKFYKY

GQELKNPLIGFGSLCPGKRMSMLQIKWFLLNILNNFSMELLDGEKTEPNTAYYGHEILPP

VNDVQVRYTLKKEAPQLVFVPRRYSS*

>TRINITY_DN358456_c0_g1_i4

MVAVSLYLAVAVIALTVAYFKFFFRTRREGEPPYVPGHLLWGNGAEFAQHAVRFLHKSQK

SLGDIFTIRLLNQYLTIITDPHCFEQFSKEKNFDFDPIQKQVNHNVFSFELKQARKMISE

AGKKVNGKYLSSGLESFADNLNTAFKTFHEQDKLDMNGNVRDGQFSQQGLRTLTANTLFN

ALFYTIFGRANQQEGPELQMEKTFNPETFHENFDIFHKFFNYLWLGVPVNVFPKACQALQ

VLCQQPVAHDMMNREGVSEYIKFSTSYMLQNGQSMQDIVGHNLVFLHVNYNTFRVAFWCV

YQLLQHQEAREAMIQELQDAIEQRRSEGDNKVGFTIEEFDKLPQLDSFLKETLRVSSGVF

MVRKVVEDTQFTMPNGQTYTVRKGDRVAMYPPAHHMDSEIFEDPEVSFSTSLQFSLRLSV

CLSVCLSVSLSRS

>TRINITY_DN358456_c0_g1_i5

MDGSLLVADSFLKETLRVSSGVFMVRKVVEDTQFTMPNGQTYTVRKGDRVAMYPPAHHMD

SEIFEDPETFKHDRFVDAKFYKYGQELKNPLIGFGSLCPGKRMSMLQIKWFLLNILNNFS

MELLDGEKTEPNTAYYGHEILPPVNDVQVRYTLKKEAPQLVFVPRRYSS*

>TRINITY_DN358456_c0_g2_i1

MDPHCFEQFTKEKNFDFDSIQKQVNNNVFNFELKQARKLISEAGKKVNGKYLSTGLESFA

NNLKQAFDNLRQQHCPASDLNGNVPEEKKLSQDGLRNLTAATLFDALFYTIFGRAKGTPE

VEQFTPKDFHTNFNIFHKFFNFLWLGLPVNLFPKACQALKVLCQQPSAHDMINREGVSEY

IKYSTNFMLQHGQSMQDVVGHNLVFLHVNYNTYRVCYWCIYHLMDKPHVYQALLEELQEA

IEIRREEGTDKVYFSIEDFDKLPRLDSFVKETLRTASGVFMVRMVMEDTEFTMPDGQTYT

IRKGDRVAMYPPAVHMDEEIFEDPEVFKYDRFVDATFYKDGKELKTPLFGFGSLCPGRRL

SMLHLKWFLINLLNSFTMELVEGERTELNTKFYGHEVLPPVNDVQVRYAIKTDAPQLEFA

KRRYYS*

>TRINITY_DN359345_c1_g1_i1

MSGTGDHAAPRSDTKSASDKTVISDNTQRCRYAPGSQDKTPVSDTAQSCGNVLSSNSGPN

STNGASNPDSNAPNSDKNTQDSDNTLCCCCDQTASQKKKGGLPPGPGGLPLLGASHRWKG

PQTNFEWAQQYGPIYSVRMGSTQLVYLNTIELVEEYMELKGETLLGRPEGPAAIANGLLF

GQGERWQENRQAFIRAMWQTPFTQHYEDIILQEVDHVVDQLSTNLGKPLELRRLLLPAVT

HRLVTVLLGQPLERDGEDMVKLVEQMTSLADDVDLMSMALNIFLKVKHFRRFLQFVSGHT

LPDMFKMSEAEQTLIKGWIAGRRAALTQSGGQADPNSLLDQLLTSQEYVNRNEEFDQELI

QSIMDLFNGGVAPVLSVLDFALLYLLYHPHIQDKMRAEIQQATADGHSISWAQRHHFPYS

HATLIEALRLASVTPSTLPHKATEDVVIEDFVVPQDVVVVASIYTLHRDPRYYDDAEVFR

PERHLDKEGRVVEPRSFRPFGVGGRLCLGYRMGEMELFLFLVKLFSTFRFRAEDPSHPPP

FRTMMRVVRR

>TRINITY_DN359822_c0_g1_i2

MAFTFSKFIDGVVLWAYRYSNFLILFLIVLIILYLWVRVPSGLPPCPKWVWPGFAHSLMV

KGDLAALFRRLRGEHGDVFSFWLAGQLVIVINGFQKIKETFIDDAANFGWRPDILNCDLF

HYGIISTSGRHGREQRQVTQSALDHLQGRGLAPMLAEEVKAFMAAVEREEQAAFDPRILT

QSAMYSTLCGVLVGHRLDYSDQKLCEGVSHFNDNIKLFSDCRLPNFFPFEKFLIGDTFDT

KFLRINMADIHRKLINEETELHLEQDSGDRTDDFLNFYMSAIVQQEELDRKTKEEAAGKK

TGKNEEKQTTINRMNMQAAVYDILWHGTVSTAAALQWALLFLLHNPDVQARAQDEIDANF

ARGSVPVSAEDRGKLPYLEAMVFEVFRLANVVPFSMAHSVLKEVKFQGYRIPTNAVVVPN

LDSVLMDPKLFPTPHAFKPDRFLSASTGQASCPAYYIPFFFGRRGCLGDKLSRDALFHFL

FGILQNYQLQPEDTGKLPPLTRGYKLVSLPQDYKLRFMPRS*

>TRINITY_DN360565_c0_g2_i1

MREQVVRTAVDKGLRMAAASTRTRNHVPSSTSVVLTGSTRPDGPSVCPHHHHHHPQPPTP

YSRCVFNGQRQVSGASDDASRAALSTTTHTATVTHAATDTSQVRSFEEMPGPRGYPVVGT

LLEYFRKENQGRMHEIQRRNHEQFGTIFKEPFGPAKNVCVADPSIVEEILRSEGQFPNRP

PYESWVLYNKLRNRRGGVMTSQGDYWKRSRRTMNPKLLKPQSVGDFISGMNSTTDEFLTR

LRRLKAAQSDRQSVQQLPFEINKYTMEAVGKVLLNVRIGTLDPNMSPKIQEFIEAIGTMF

LTGHQLMAFAKVHQILRTRVWRQHVQAWDTIYTISNELMADQVDAARQRVEEGQCPAPGD

KLDLISHMVSAGTLSREEIIDNLTETFMGGVDTVANGVSFMLYLLSINQSVQSKLRDEVD

SVLGQRWCTMNDLQAMPYVKAVVKETLRLFPPIPLNARVTQEEVVISNYRIPKGTIIMLN

NYTMSRDERIFSDPDAFVPDRWLRAEVSSWHPFSAIPFGYGVRSCVGRRVSENLMYVAAI

QMVQNFVLKKDPSYDITPLVRTQFTPGPELPVLFEER*

>TRINITY_DN361005_c0_g1_i1

MVSLQGYVLSENAKGGATYALVGAVVILAVSWLFGKWQREHGTEGKIPPHIPSSIPFLGQ

AVSFGQSPIEFLLAAYQKYGPVFSFTMVGKTFTYLIGSDSAALFFNSRNENLNAEDVYSR

LTTPVFGKGVAYDCPNPVFLEQKKMFKTGLNIARFREHVHMIEEETISYFKRWGKSGEKN

LFMAMSEVIILTASRCLHGKEIRAKLDESMARLYMDLDGGFTHEAWLLPGWLPLPSFRKR

DQAHQEVKRIFYEVIQQRRDSKETEDDMLQTLIDSKYKSGRNLTDDEIAGMLIGLLLAGQ

HTSSTTSTWLLFFLAKHQDIQEAVLEEQKRVCGVSGDSEWPPLDYDIVKDLQLLDRCLKE

TLRLRPPIMTMMRMCRTTQTVMGYTIPPGHQVCVSPTTNHHLPDTWKDVGKFMPDRFLDA

DVANSEKFAYVPFGAGRHRCIGESFAYVQIKTAVSTILRTYKLELVDGYFPEVDFTTMIH

TPKRPIIRYSPRTL*

>TRINITY_DN362126_c0_g1_i5

KEFNKYSDRYSGLEPIISDFSWKMTGNAKVPAWTRYRHVMTPAFTTGKLKALEGYIIQCS

KVLCQVLKDKADKQEDIDLKAIFQRFTMDVIAGTAFGLDTNLQRGEDTQTHDMIKAMKGI

LSNLSSTGIILIAVAFPFMAPLLRAMGYDMFPPKDLNFFKQCISAIIHERDQNPEEAKKR

VDFLQQMLDIRVAPGEEVTDLDDASFGTKTNKKLTEEEVVAQCILVFNAGFETTSSSLRF

LSYCLATNPDIQEKLYQEIQEVIGDGELRYEQLQELKYMDLVVTETLRMYPVVPGVTRLA

SETVNVKGYCIQKGMPVLIPIYMATHDPEFY

>TRINITY_DN362368_c0_g1_i1

MGPNMAVMSPLMQAAGVLVVTVVTVKAVRWLLWYRDYFRFFNSLPGETSFSWIWGNLHVV

RGQDAEGRLRYQHTLVQKYPKMYRFWLGPFRAVISLVHADAIRDVLKTTEPKPNMYNFAL

PWLGNGLLLANGDRWARSRRLLTPAFHFDILKPYVAVSNSAADQLVNRISDHVREKRSFE

VFSLISLCTLDVILQCAMSYNDDIQNKGETHPYVQAVNELSELWFARGRNPFVYSDFIFK

LTSSGRRFMKQCHFVHSVSETIIHARQDKLMKEGASKKKYLDFLDILLTAKDETGQGLTP

LEIRNEVDTFLFEGHDTTASAISWIMYSLAQHPQYQEKAQAEVDAILQGRESTNIEWGDL

SKLEYLTQVIKEGMRLHCPVPFIQRQMTRPTTIEGVTLPEGTQCTIHLLNLHHNPAIWKD

PLTFDPDRFHADNIKDQDTYAFVPFSAGPRNCIGQHFAMNEEKVILSHLLHKFSFTLDKN

HVVKKRPAAVMRAENGIKIFATLRR*

>TRINITY_DN362368_c0_g1_i2

MMLLSVDLLCYRNPFVYSDFIFKLTSSGRRFMKQCHFVHSVSETIIHARQDKLMKEGASK

KKYLDFLDILLTAKDETGQGLTPLEIRNEVDTFLFEGHDTTASAISWIMYSLAQHPQYQE

KAQAEVDAILQGRESTNIEWGDLSKLEYLTQVIKEGMRLHCPVPFIQRQMTRPTTIEGVT

LPEGTQCTIHLLNLHHNPAIWKDPLTFDPDRFHADNIKDQDTYAFVPFSAGPRNCIGQHF

AMNEEKVILSHLLHKFSFTLDKNHVVKKRPAAVMRAENGIKIFATLRR*

>TRINITY_DN365356_c4_g1_i1

MLESLTSPGDMLTPQSLILEQLQAWAGKAATPLTLLFASALLGSVLKWTRRHHHEHQTAG

PLPPSPGLALPLVGHLHLMKGGSDPRVLFADMRDKLGDIFSFYAGSRLIVVLNGYDVIRE

ALVKKAAVFSHRAHTFMSQKIGQGRGIVNTSGRHWREQRQFAERALRALGVGRTSVMSLK

IQKEVDCLLERLEMSRCQGQPVDPGPLLQASFSNVIISIIYGHRFELDDPKFLNLMTALT

NCFKNFGNTEPLNFFPAVRFLPGDPYRYWFCIQNMNFLESHLVYPELTEHEQKLKESAES

EDVISSYLREMTNKLTNGMPTFMDKENLVKVAGDLLAGGTEPPSITTMWILLYLIHFPDV

QEKCYQEILAQIGTERSPTLEDRSSLPYLEATIMEIQRHADVGPLAMAHGLARDTEFRGY

SLPKDAIVLINIHSVHHEVKGWGDPEVFRPERFLGEDGKVVHHNNFVPYSMGPRVCPGEN

MARMEMFMYVSCILQRYRLKPHASTPPSLEGYMTLMHHPDPFTLMVEKRE*

>TRINITY_DN365966_c0_g1_i3

MDSDSLSLLKSTLLFSFVGYILFRMVSAIQNLRSYVKIFHNCPGEADYHWVYGTLHKYPG

PNEAGIRYDIDAMKKRPRFQRVWAGPFVPILIFYHPDLVRLVLKSSAPKPRGGLMVSVYE

MGLGWLGEGLLIANGSRWARSRRLLTPAFHFDILRPYILVKNKAADVLLSKLQTYAEEKT

SFEVFHHVGLCLLDVLLQCAFADERNCQTTEKTDPFVQTVNELITVWTERSLKPWQHIDG

LFRLLPVGRRWKKLCDFAHSVSEELINKRRKILEESQDESQDDSEEMSEEDESKPGKKRL

MSFVDILLTAKDEDGKGMTPLEIRNEADTFLFEGFDTTTSALSWTLYAMARWPEHQAHVQ

EEVDAILKDRSSDHILWEDLSCLPYTTACIKEAMRNYSTVPFIERETLEPLNVDGHIIPA

GTFVAIQLWCLHHNQAVWNRPHDYLPERFLGDNLTKMDPFQFVPFSAGSRNCIGQSFAMH

ELKVMVARICHRFTLTLDHDHEVLRAPLATFKAEKDIKLFVTPRQH*

>TRINITY_DN365966_c0_g1_i4

MDSDSLSLLKSTLLFSFVGYILFRMVSAIQNLRSYVKIFHNCPGEADYHWVYGTLHKYPG

PNEAGIRYDIDAMKKRPRFQRVWAGPFVPILIFYHPDLVRLVLKSSAPKPRGGLMVSVYE

MGLGWLGEGLLIANGSRWARSRRLLTPAFHFDILRPYILVKNKAADVLLSKLQTYAEEKK

SFQVFHHVSLCLLDILLQCAFAAERNCQTTGENDVFIQTVNQLLVVWNKRGLKPWLAIDW

VFRLSSEGRRWRKLCDTAHAMSEELISKRRKILEESQETSSAEYESKPGKKRLTCFVDIL

LMAKDEVGKGMTPLEIRNE

>TRINITY_DN365966_c0_g1_i6

MVSVYEMGLGWLGEGLLIANGSRWARSRRLLTPAFHFDILRPYILVKNKAADVLLSKLQT

YAEEKTSFEVFHHVGLCLLDVLLQCAFADERNCQTTEKTDPFVQTVNELITVWTERSLKP

WQHIDGLFRLLPVGRRWKKLCDFAHSVSEELINKRRKILEESQDESQDDSEEMSEEDESK

PGKKRLMSFVDILLTAKDEDGKGMTPLEIRNEADTFLFEGFDTTTSALSWTLYAMARWPE

HQAHVQEEVDAILKDRSSDHILWEDLSCLPYTTACIKEAMRNYSTVPFIERETLEPLNVD

GHIIPAGTFVAIQLWCLHHNQAVWNRPHDYLPERFLGDNLTKMDPFQFVPFSAGSR*

>TRINITY_DN365980_c1_g1_i1

MADTEGWDSLTTALVIGVFTLVLFYIWRTYRYLTTFSRMGIPGPRPLPFVGNMIPFLKES

SLEVTQRFDKEYGKVYGIFFPVPTLVVTDKELAREILTKKFSSFADRNDLPGFTSGAMKD

SLLSIKGDHWKHVRKQLSPTFSSYRLKQIVPVLSKVADNLRLHLEDKAKAGQMVELKELC

QNIMVDCIAGVAFGLEVDSLKNPDDPFYRHSDHTLSFFNKMMGLLMAFPSLEPLLRLFGI

HAFPQSDMSYLVSVINAALEDRRQDKQKYADFLQLLVEAEKAGSEQGQVDADIDFTHQLK

TSSQWTRKGLTLSEIHANSITFLLAGNEQVSSVMSLTLFALAANPHCFQKAREQVDLKMG

KGKPDYDSVMDLTYLDMYMSEAIRYYVPGMLMRVATEDVDLGAYHVPKGMQILIPSCVMH

HDPQEWSDPDVFDPERHAPENRGQRDQVSFLPFGQGPRSCIGIRLAQLEIRLILATVLQH

FTPVLCEKSVVPPVMMPLTVGNTRDGLWVRFEPRADTQ*

>TRINITY_DN368246_c1_g3_i1

MSDLLQTVQDFCQQAIVAVKDASVTAPGTITTQALAVGALMGFLAYKLMKKRYRLPPGPW

PLPLLGNLLSMTSSEQLYTKLARWAKENYGPVISFYFGPVLCVTLNDMQSINEALVLKGS

DFAGRPFLFSLDLVTEGSKDIAFADYTPAWKLHRKISMQAIRHYMTGSHLEQVVHKALAM

VTERMAAEHGPFDPHPYNTALMFHIIDFICFGESKPYDDPSIQKLADIFESVNEKLGNGF

FEDIIPLLKYWPTKKFQHFLIENQELLDYMETNIQKHRERFSPDNIRDITDSLLMAQKEA

AREESAEVMAMFTDAHLRETVSDLFGAGVDTSRITLDWAILFMAGHPEIQKRAQAEIDAA

TGGRMPGVGDRSKLQYTEAVLYETLRLGSVAPTALPHKTTCDTTVGGYDVPKDTMVLVNI

WAVSNDPKLWDQPEQFNPERFLDDQGKLKTRPEGWIPFSAGRRVCLGESVAKPELLLLLA

CFLKRFTISLPEDIKFKTDHQLAGLLVHAPKPYKIVVTPR*

>TRINITY_DN368246_c1_g3_i3

MSDLLQTVQDFCQQAIVAVKDASVTAPGTITTQALAVGALMGFLAYKLMKKRYRLPPGPW

PLPLLGNLLSMTSSEQLYTKLARWAKENYGPVISFYFGPVLCVTLNDMQSINEALVLKGS

DFAGRPFLFSLDLVTEGSKDIAFADYTPAWKLHRKISMQAIRHYMTGSHLEQVVHKALAM

VTERMAAEHGPFDPHPYNTALMFHIIDFICFGESKPYDDPSIQKLADIFESVNEKLGNGF

FEDIIPLLKYWPTKKFQHFLIENQELLDYMETNIQKHRERFSPDNIRDITDSLLMAQKEA

AREESAEVMAMFTDAHLRETVSDLFGAGVDTSRITLDWAILFMAGHPEARN*

>TRINITY_DN368246_c1_g3_i6

MSDLLQTVQDFCQQAIVAVKDASVTAPGTITTQALAVGALMGFLAYKLMKKRYRLPPGPW

PLPLLGNLLSMTSSEQLYTKLARWAKENYGPVISFYFGPVLCVTLNDMQSINEALVLKGS

DFAGRPFLFSLDLVTEGSKDIAFADYTPAWKLHRKISMQAIRHYMTGSHLEQVVHKALAM

VTERMAAEHGPFDPHPYNTALMFHIIDFICFGESKPYDDPSIQKLADIFESVNEKLGNGF

FEDIIPLLKYWPTKKFQHFLIENQELLDYMETNIQKHRERFSPDNIRDITDSLLMAQKEA

AREESAEVMAMFTDAHLRETVSDLFGAGVDTSRITLDWAILFMAGHPEIQKRAQAEIDAA

TGGRMPGVGDRSKLQYTEAVLYETLRLGSVAPTALPHKTTCDTTVGGYDVPKDTMVLVNI

WAVSNDPKLWDQPEQFNPERFLDDQGKLKTRPEGWIPFSAGRRVCLGESVAKPELLLLLA

CFLKRFTISLPEGAKFKLDHQMAGFSAHSPKPYKIVVTPR*

>TRINITY_DN368918_c0_g1_i2

MMTVTVAVLSVAAVVAMSFLLIKFVMAVSDYRKLCLVYRQFPHNPGNLIWGNLFEYPGPD

DRGLKFQREMTATFPRVLSAWMGPFFPMIIISHPDTIKIVLRTSEPKGQRIYHLVEPWIG

NGLLLSKGQKWNRNRRLLTPAFHFDILKPYLNIKNKAADTLLEKFQGYHERGEYFEVFGD

ISKFTLDVILKCAFSYDIDCQRLGDQHPYVKAVFALSELAVKRFFEPWVHSDWIYFLTPS

GRKFRKHCQEVHAVAEEVIQKRKQALANGLKPGPGEQGHKRRCLDFLDILLTAKDENNEG

LRPDEIRAEVDTFLFEGHDTTASAISWALYSLAEHPDIQADCQREVDELFEGRKTDDVVW

EDFSKLPYLTMCIKEAMRLHSPVPFIQRQLTQDTLIDGHIVPAGAVVNIVIYNIHHNPVV

WEDSMEFRPERFTEDNCNARSPYAFVPFSAGPRNCIGQTFAMHEIKLILARILHKYTLEL

DPHHKVEKFESLVMKTKTGIRMKAIPRQSAC*

>TRINITY_DN368918_c0_g1_i5

MMTVTVAVLSVAAVVAMSFLLIKFVMAVSDYRKLCLVYRQFPHNPGNLIWGNLFEYPGPD

DRGLKFQREMTATFPRVLSAWMGPFFPMIIISHPDTIKIVLRTSEPKGQRIYHLVEPWIG

NGLLLSKGQKWNRNRRLLTPAFHFDILKPYLNIKNKAADTLLEKFQGYHERGEYFEVFGD

ISKFTLDVILKCAFSYDIDCQRLGDQHPYVKAVFALSELAVKRFFEPWVHSDWIYFLTPS

GRKFRKHCQEVHAVAEEVIQKRKQALANGLKPGPGEQGHKRRCLDFLDILLTAKDENNEG

LRPDEIRAEVDTFLFEGRTFPSCPT*

>TRINITY_DN368918_c0_g1_i6

MMTVTVAVLSVAAVVAMSFLLIKFVMAVSDYRKLCLVYRQFPHNPGNLIWGNLFEYPGPD

DRGLKFQREMTATFPRVLSAWMGPFFPMIIISHPDTIKIVLRTSEPKGQRIYHLVEPWIG

NGLLLSKGQKWNRNRRLLTPAFHFDILKPYLNIKNKAADTLLEKFQGYHERGEYFEVFGD

ISKFTLDVILKCAFSYDIDCQRLGDQHPYVKAVFALSELAVKRFFEPWVHSDWIYFLTPS

GRKFRKHCQEVHAVAEEVIQKRKQALVCILCVYVCVFPCVPACMCK

>TRINITY_DN370357_c0_g2_i1

ATALQFLLYLMAMNPDTQEKLYQEIVAAVGNDEPTYENVSNIRFLDSCVRETLRMYPPAT

LFDRVVINTRTINGVTIPAGAGVVANVVLMMKDEEHFPQPDHFIPERFEDNDVPISSFMR

EMVFGAGPRQCIAMRLAVYEIKMAAVSILRKFRVVKVKDTPEKIIMKPGTIFHVSLKPVW

VGTEYRAAE*

>TRINITY_DN371969_c0_g1_i1

MWLLSSLSLDITTLLLGGVLLLCLLWWLSTRRPPGIPPGPGGALPLLGHMHLLKPDPRAQ

YRAWRRQYGDVFSLYMGGKLMVILSSYSAIREALVKFGDVFSHRPNSFTTEKILQGNGIS

FSSGQQWKEQRKTALEILREMGFGKNMLAEKIQKEITHYIQVILGHQGAPFGFGQLTQIS

VSNNMTSIIFGKRFQYEDSEFKSYLECVNRTMKLLPETGILNFLPALDYIPGDPFHVKEI

LSNERFHFGFIDSQLADHNVALDPHRIHYQMTPGEEKGEEDKRENGEKQDKGEEDGEERD

TDYIYCYLREIKKHKAAGHVDTTLGGDNLRMSTSDLFVAGTETTTSALQWAVLFFLHNPD

VEEKCFKEISEVIGFHRYPTMQDRPDLAYVEATIWEVLRKGDIVPFSLLHASSEDVTFLG

YTIPKDTILVPYLSSILQDPEIWGDPENFRPERFIGPDGKLTRPDEFIPFSIGRRACLGE

SLARMELFLYLTTLIQRFRFLPAEEGKLPPLEGVLGMTHPPQPYTVRAVPRI*

>TRINITY_DN374003_c1_g1_i2

MPSLKFTMDLPTATTTTTTFLIATAATLTLALLWWRSTRRPPDIPPGPGPALPIIGHFYL

MEKDPRGQFRTWRKQYGDVFSLYMGSRLMVVLNGYEVIREALVKNADIFSDRPSPSMFDL

VTKAKGVGDTSGPLWKAQRKTMLEILRELGMGKNRMAEKIQEEIDEFIKVISSKNSVPFD

PSRLVQVSLSNNICSITFGERSDYDDQHFLHLMESMEEMFLLAGGASFTAFLPFLRHLPG

DPFHIHKAMDCYNYIVDTFLVPAIEQHVQDEEEGKGDDFISAYLRECKRAPESGIADYVN

EFNLQKVLMDLFAAGTDTSATAIRWALIYFLHYPEVQEKCYQDIVQALGHPPSRTVELAD

RAEMTYLEATINEVLRVAEVVPLGVLHAPACAVQFHGYTIPEDAYILPNLDSVLHDPTVW

DEPENFCPERFIGVDGKLVRMVEFLPFSLGRRVCLGESLARTELFLYLSTMIQRFRFLPP

EDGQLPSLQGDVGLVHAPKPFLLRAVPRN*

>TRINITY_DN374704_c0_g1_i2

MSAALVLVGVVSAVCALVWFLQTARLRKLINQIPGPASIPVLGNAHQMTSGPEWYKQVMQ

WGQTYRQEGIFKLWLVNKPVVGVTKAETVEVLLNSSKHIDKAGEYDFLHVWLGTGLLTST

GEKWRSRRKMLTPTFHFRILHDFVEVFNMQSKVLIDKLQQHANKGPFNIFQDIALCALDI

ICDTAMGQHVSAQTKDSEYVRAVYKMSGLVEHRMRSPLYWNKTTYDLFGPGKEHDRCLKT

LHDFTVKVIKDRMKNFDAKRAEAMLGDVNSNSRGNNTLATEDEVCSRKVRLAFLDMLLYM

SDNGRALSIDDIQEEVDTFMFEGHDTTAAAMNWCTYLIGCDDKVQEKVHEELDRVFGGSD

RTPTMNDLKELKYLECCIKEALRIFPSVPYYGRTTTEEAKFGPYTVPEGVTVVLFAAAIH

RDERFFPDPDRFDPDRFLPENAAHRHPYTYIPFSAGLRNCIGQKFALLEEKSVLSSVFRH

FRVKSCQTREELLPVGELILRPQKGVLVELTSR*

>TRINITY_DN375714_c3_g1_i1

MLDFVIFAVTAVVVLLIAVIYMYPSSRRISTIPGLDSTSAQDGNLVDIQRAGSLHQFLLQ

QHSERGSITSFWIGDEFVVSIASPGLFKQQTNVFDRPGELYSIMLPVFGEKSICFLNGAE

GRARRQLYDKCLQHEKLHEYSQLLNKISTDLATKWGTVAKGEHISLQQYMSAFAIKAVLQ

CVMGSCFTDDKEILSFMRHFDQVWVELEYRIRDPEIPADDSPRGKMFESALKNMRALISH

ALEHRIKNKRGNKEELIVDHIIAAHKNDTDGQVGDCLTYLVTGIPRLASMLTWCMYFLAS

HPEVQDKLNAELQHEFTGDSSPAAVFKLRYLRQVLEETMRCAVLIPFAARCQDFSTEIGG

HKIPKNTAVIQALGVVMQDEKLFPVPNKFDPDRFSVENSKERDTLAFCPFGFASRRHCPA

KDLTYMMASALVAILIRKFSVSLVEGQVVTPFYGLVTKPEDDVWVTVQKRK*

>TRINITY_DN375757_c3_g1_i6

MEILGLVDIPLWLLLLLSFTLLLYVYGTWTYSTWSSQRIKGPKPWPLFGNSREIFKKTYF

RVFSEWQQKYGRVVGYYAGVRPALMVTELDMLREIMVKYFNHFTDRNILPGLRPREVDMG

LFFAKGAAWKRIRTTMTPTFSTGKLKLMSHYVHRCSDLLTKSLENKARSGQLIDVKDVFG

SFTMDVIAGTGFGLETNSQTEEGEPFVQHCKGLFASIGLRGALRAILYLAYAFPILAPYV

GMLGVSAFKTDHVEFFFAALETMINERREMNMNSTVKNGRKTVDLLQMLVEAEADPEDIH

DKADDSDDSKNCRRMTKEEIKAQGFIMLIAGYETTASSLQYLAYNLALYPDVQEKVFEEI

KDILGEDDPTYENVSELKYLDCVIHENLRMFPPVPLIDRKADEDLVIKGVRIPAGCGIAI

PIYALMHDPEFFPEPHVFNPDRFLEENIRRLDPLLVELPFGHGPRKCIGIRLALLEIKFA

AVRICRQFRFVKCAETPEKIRLGKLIGLSAPDVTIMVKAEIRSA*

>TRINITY_DN376007_c3_g1_i1

MDEGKVEGAESGGLMDEGKVEGTESGGLMSMSLTGWLVTGIVVLLVLWIWKSYRSFTVFS

KMGVPNPPPLPIFGNMFDFFKYGFVGSMLHYTEHYGKVCGVYGVTGPTLVIADMDMLRDI

FVKKFQSFPNRITRAQMQYKPWKDMLTQLEGDHWKHVRSTLSPTFSAGKMKRMMPAIQRI

AVSLTDYLNDVAAKGDMIELKDICGRFSMDVIAGTAFGFDVSSIQQPDHEFVKHAHSIMN

PSKLQVSLIMLFPNLAEFLNRHVVRGSMKSSLDFFMGVTDAALKERYTDMQRHHDFLQLL

VEAEKEGNQGPVDAEINHSEQLTTSEKWTRKGLTKDEIQGNAFIFLLAGYETISTVMSFT

LFCMANSPEALKKAQDEVDRILGKKVADYESAHELSYVDMCVNEAMRLFPPGFILSRKPT

EDVEVKGLKIPKGMGVLVPVVNLHKDPDVWPEPDTYDPLRFTTEEKGKRHPFSFMPFGMG

PRNCIGMRLAQLEVRMGVATVIQHFTPILCEKSVYPPKLSKIRMQANDGLWIKLQKRE*

>TRINITY_DN376007_c3_g1_i4

MDEGKVEGAESGGLMDEGKVEGTESGGLMSMSLTGWLVTGIVVLLVLWIWKSYRSFTVFS

KMGVPNPPPLPIFGNMFDFFKYGFVGSMLHYTEHYGKVCGVYGVTGPTLVIADMDMLREI

LVKKFQYFPNRITRAKMNYKPWKDMVSEMEGDHWKHVRSTLSPTFSAGKMKRMMPVIQRI

TVCLTDYLNDIAAKDEMIELKDICGRFSMDVIAGTAFGFDVSSIQQPDHEFVKHAHSIMN

PSKLQVSLIMLFPNLAEFLNRHVVRGSMKSSLDFFMGVTEAALKERYADTQTHHDFLQLL

VEAEMDENQGPVDAEINHSEQLTTSEKWSRKGLTKDEIEGNAFTFLVAGYETVSTVMSFT

LFCLANNPEILKKAQDEVDRILGKKVANYESVSELSYVDMCISEAMRLFPPGFILSRKPT

EDVEVKGLKIPKGMGVLVPVVNLHKDPDVWPEPDTYDPLRFTTEEKGKRHPFSFMPFGMG

PRNCIGMRLAQLEVRMGVATVIQHFTPILCEKSVYPPKLAKMRMLANDGLWVKLQKRE*

>TRINITY_DN376021_c0_g1_i16

MDIFSSALVFLIIVLVVYFFTTREKHSKPLPPGPRGWELVRAFFSSLNGTLIYQTKIWAR

KYGDVVFCPLPQGNLVFLNSRSVTKELFTGKATEKFANDRMWSYAADKLCLRKCIGFASL

SSPAWTKMRKLLNSMLKIYGESVAKFEATNTEALDHMMTALDSQCGQDIELVGYLNHSLH

SVMAILLTGEVQDDARVRAMAKFNAVIIKILNPAFSFPVKMCPILDYIPGTIYTKTRQEM

ERRKAEAMKIYFEDMRDTREPGSPRGFVDHLLEYQSKEGAQWMTDDHAKGLMMDITAAGI

ITTTHTLNACIFDLLHRQDIIRKIQEEIDRVIGHDRRPALSDRSSCHYTEAVVWESLRYS

VT

>TRINITY_DN376021_c0_g1_i9

MDIFSSALLFLIIALVVYFFTTRQKHSKPVPPGPRGWELVRAFLGTLNGTLINQTEEWAH

KYGDVVFCPLPQGNLVFLNSRSVTKELFTGKATEKFANDRMWSYAADKLCLRKCIGFASL

SSPAWTKMRKLLNSKLKIYGEGVARFEETTAESLDHMLTTLDSQCGQDIEILDYLNHSLY

SVIAILLTGEVQDSERIQALRQFNSCITKILNPSFSFAMKVCPVLDYIPGTYHTKTRREM

EKVKAVVMKYYLEDVKNTREHGSPRGIVDHALEYQSNEGAEWMTDDHIKGLIIDIIVAGI

ITTTLTLNACMFYLLHRQDVMRKIQEEVDRVIGSGRKP

>TRINITY_DN377631_c4_g4_i2

MAGEESISFGISLIDLKATAAVFAIALLVIKLMTAVNKKRYKLPPGPRGLPIVGYLPFFG

PEPPVTFTQMRKMYGDVISITMGSWPAIVINGRDAIMEALVIKGDDFSGRPAFTTARLLN

NGRSLGFASFGPIWKVHRKIVSNVLYTFSNARKNPIEDIIRSDAKIVIREFLSHGDKSFC

PLNSLKVAASSMVYQLCYGRQDNIRDDKDFMGFVNGMREFTEFTGTGNPVDVMPWLRYVM

PSKVSKFLELIHVSIKCRCKKVEEHESTFDATNLRDITDGVIHAGNNLSEGEKAVGLDKR

WVIESLDSIFGAGVNTVSTVLEWFVGLMAAYPDVQQTLFQQIDTVVGQGRDATLADRADL

PLVEATLYEVLRYTASFPFTLPHMTTCDTTLQGYDIPQGTVVLVNLYSIFMDKELWGDPN

TFRPERFLDTEDHLDRALIEQVSTFSLGRRRCVGEFLARMEMFLFCTTLIQRLKVFKPPG

QPGIVLKAHFGLTRDVEPFEVRVSQRD*

>TRINITY_DN378118_c1_g1_i1

CKASCSRAALLYTCAHLSTVSRINSGCHSKVPDWLNSVSWRSGTPTRQFQLVPFLDFCCS

SQPSRGGLRRPKQLGTAVDDNMSWLGVAIGVGIAVVFLSLVFLILWLFPDDGKRPKPVRR

KRERTLCTYQLRGDVDVPESEETYKQVVTLRSCFRFREDDSRRSTVPGMEPSSNQNGNIN

DIGKAGSLHEFLMGLHKDFGPIASFWWGTQYTVSISSAELFRQHQNLFDRPPELFRMFEP

FLTPFSIQYANGSDGKLRRQAFDKVFNYDGLGIYYEKLQKVAIDITKKWDHAEADDHFPL

GEHMFLFAVKAALVALMGDKFKDDKEAMAFKHAYDVAWEDMEQRLINPILPKENSARAQN

FKKALSALKDIVARAVKERESRGKDSRDFLLIDAIIGHHPDDEERRFADAITYTVGGFHT

TGNLLTWCIYFLCMHEDAQEKVYKEINDVLGPTDPVTHQTIGKLKYLRQVLDETLRCSVL

APWAARFSDEDIELGGHRIPAGTPVVHALGVSLMDEKVFPAPMVFDPERFSEKRSKGRPT

VAFSPFGFAGRRQCPGYRFAYVEASIILVTAIQKFKFLLVPGQDVKRHYGLVTHPKEEIW

FKVVKRE*

>TRINITY_DN378118_c1_g1_i2

CKASCSRAALLYTCAHLSTVSRINSGCHSKVPDWLNSVSWRSGTPTRQFQLVPFLDFCCS

SQPSRGGLRRPKQLGTAVDDNMSWLGVAIGVGIAVVFLSLVFLILWLFPDDGKRPKPVRR

KRERTLCTYQLRGDVDVPESEETYKQVVTLRSCFRFREDDSRRSTVPGMEPSSNQNGNIN

DIGKAGSLHEFLMGLHKDFGPIASFWWGTQYTVSISSAELFRQHQNLFDRPPELFRMFEP

FLTPFSIQYANGSDGKLRRQAFDKVFNYDGLGIYYEKLQKVAIDITKKWDHAEADDHFPL

GEHMFLFAVKAALVALMGDKFKDDKEAMAFKHAYDVAWEDMEQRLINPILPKENSARAQN

FKKALSALKDIVARAVKERESRGKDSRDFLLIDAIIGHHPDDEERRFADAITYTVGGFHT

TGNLLTWCIYFLCMHEDAQEKVYKEINDVLGPTDPVTHQTIGKLKYLRQVLDETLRCSVL

APWAARFSDEDIELGGHRIPAGTPVVHALGVSLMDEKVFPAPMVFDPERFSEKRSKGRPT

VAFSPFGFAGRRQCPAYRFAYVQASIMLVTVIQKFKFLLVPGQDVKRHYGLVTGPKEEIW

FKVVKRE*

>TRINITY_DN378118_c1_g1_i3

CKASCSRAALLYTCAHLSTVSRINSGCHSKVPDWLNSVSWRSGTPTRQFQLVPFLDFCCS

SQPSRGGLRRPKQLGTAVDDNMSWLGVAIGVGIAVVFLSLVFLILWLFPDDSRRSTVPGM

EPSSNQNGNINDIGKAGSLHEFLMGLHKDFGPIASFWWGTQYTVSISSAELFRQHQNLFD

RPPELFRMFEPFLTPFSIQYANGSDGKLRRQAFDKVFNYDGLGIYYEKLQKVAIDITKKW

DHAEADDHFPLGEHMFLFAVKAALVALMGDKFKDDKEAMAFKHAYDVAWEDMEQRLINPI

LPKENSARAQNFKKALSALKDIVARAVKERESRGKDSRDFLLIDAIIGHHPDDEERRFAD

AITYTVGGFHTTGNLLTWCIYFLCMHEDAQEKVYKEINDVLGPTDPVTHQTIGKLKYLRQ

VLDETLRCSVLAPWAARFSDEDIELGGHRIPAGTPVVHALGVSLMDEKVFPAPMVFDPER

FSEKRSKGRPTVAFSPFGFAGRRQCPGYRFAYVEASIILVTAIQKFKFLLVPGQDVKRHY

GLVTHPKEEIWFKVVKRE*

>TRINITY_DN378118_c1_g1_i4

MAFKHAYDVAWEDMEQRLINPILPKENSARAQNFKKALSALKDIVARAVKERESRGKDSR

DFLLIDAIIGHHPDDEERRFADAITYTVGGFHTTGNLLTWCIYFLCMHEDAQEKVYKEIN

DVLGPTDPVTHQTIGKLKYLRQVLDETLRCSVLAPWAARFSDEDIELGGHRIPAGTPVVH

ALGVSLMDEKVFPAPMVFDPDRFSEKRSKGRPILAFSPFGFAGRRACPGRRFAYVEASVM

LVTAMQKFKFLLVPGQDVKRYYGLVTRPKEEIWFKVIKREQDPGGNVDQSGKNDA*

>TRINITY_DN378236_c0_g1_i1

IKEYIEDSITGIFVILLKGEFDKKAGKAMTKFNATALPVLDFSFTFAIKILPFLDYIPGT

SYTQARRNVEEGKKNLMECVLEETKATRVPGKQRGIVDKILDFQSNQGTDSMTDDHVKGI

IMDIVVAGITTSTHTLTTMMFYLIHRPDVLHRIQEEVDKLIGPDRKPTLNDRRKFHYTEA

FILETMRVLSLLPLGADHYISEDLELKGYFIPKGSVAFSATYAFHKSKELWTDPDSFKPE

RFLDDEGELLPATHPTRLSLLPFGTGKRSCPGETFARARIFLFITTLFQHFNILPPAKEP

TLAMERDSWIPGFVIAMKPYRCRFVKRQTKA*

>TRINITY_DN378506_c1_g1_i2

MAELPGSVGWPLLGDKSIDFYKDPVKFVRKNIEQHQSRIFAARFLNKPTAFVCSNAGVRE

ILADRDNAFDLGYKAFMGQIFGDNILFTDGDEAHMFRDALAHLFTSDALQSYQDIIRRIT

QKHVEAIDHRKPLCLYWFFKRVVTEISLTLFLGLDFADEKEAAKVVSLTIKHWHGIISVP

VKIKLPGTGSESSFGKAMDAKKSLLEIIKSQRVKAQEGFVQRMEHISHKEEDVFVNNHLL

LFTSALVPKAISSILTSFAVQVGLSDMMGVQREVLANAELKTSLFREVHRMFPPFLGGRR

VAKKDIVIDGYKIPADHAVVYMTGEAHRDPAVFTDPDTFQHQRWNTDRGLSEENLFGFGY

GPRGCIGQKLVWNIIDEILIQLLSRYNWTLQEQQDLTHKQLPVSRPKGAVMVNLTPVTSP

GQTGDIPDTVCPQPAHLASGDSSAAS*

>TRINITY_DN378921_c1_g3_i4

VLKLLGTTSVVYILPFLRHVPGDLFKVQRVVNQYQHIVNTFAIPAIQQHIKDYREGQQED

FISAYLRETKSAPERQLADIVNEFNLEKVIMDLFVGGTDTTSATIRWAIIYLLHHPEVQD

RCYQDIVNCIGTSRPPEVGDRAGMVYVEATIMEVLRICDLVPIGVIRSTSSSIQFRGYTF

PKDVHVIPNLDSVLHDPAVWTDPSIFRPERFIDDEGRLVKPDQFLPFSAGRRNCLGESLA

RVELFLYLSTMIQNFRFLPPEDGQLPSLQGNMGLIHSPRPFLVRAVPR

>TRINITY_DN378921_c1_g3_i6

SSMIYVFPFLRHVPGDLFKVQRVVNQYQHIAHTFAIPAIRQHIKDYREGQQEDFISAYLR

ETKNAPERQIADIVNEVNLEKVIMDLFVGGTDTTSATIRWAIIYLLHHPEVQDRCYQDIV

NCIGTSRSPEVGDRAGMVYVEATIMEVLRICDLVPIGIMRSTSSPIQFRGYNIPNDVFVI

PNLDSVLHDPAVWIDPSTFRPERFIDDEGRLVKPDQFLPFSAGRRNCLGESLARVELFLY

LSTMIQNFRFLPPEDGQLPSLQGNVGLVHSPRPFLVRAVPRN*

>TRINITY_DN379538_c0_g1_i2

VFTKQFIHFPNRANLRLMDYKPWSDSLVLLKDDHWKHVRSQLTPTFSSGKLKKMMPAVER

VTQNLDKYIREKAQTGEEVELKALAASFALDTIAGTAFGLQVDSLANPSDPFNTNALSLL

RPCSNLLSLMFSFQWVNTLLRCCFGITFFPAKPTQFFYNILTTALKERRLDPKSYNDYIQ

LIVDTEREGGAKGAKGAIDPEIDHSAHLTTSGTWNKKGMTDDEVVANGLVFFAAGYDTLS

TAMSFTLFALANHPEVVIAAQQEIDEKLGSKPVSHNNIAELTYIDMCLNEGLRLYPPVPN

LNRELADDLKVGPYQMKKGMRVLIPIWSIHRDPENWSEPEKFIPERHTPEARANRHPLSF

LPFGYGPRNCIGMRLAQLEIRMALA

>TRINITY_DN379927_c14_g3_i3

PQPIMQFKPWEDTLPQLRDDHWKHVRSQLSPTFSSGKLKKMIPAICRVLENLEKHVGEKA

QTGEEVELKYLAGCFTMDAIAGAGFGIQVDSLANPNDPFIVNGNDLLKQRVWLFPLYMVF

PWLGPLLRKVGLSVLPVKPTNFFVDILTLALKERRLDRKPGRMFYHGCNSRGRIWHTSGF

PGKS*

>TRINITY_DN380295_c2_g1_i1

ADYTPTWKLHRKIAMQAIRHYMTGSNLEKVVHDALTTVADQMAAEPGPFDPHHYNTTLMF

HIIDYICFGEAKPYNDPSIKKIQDIFDQVAKISGNGFLEDVIPLLRYWPTKKFCMMMTLF

QPFMDYMESHIQKHRQTFSPDHVRDITDSILLAQMETAREESAEVMAMFTDVHVRETIAD

LFGAGVDTSRFTLDWAMLFMAGNPEIQKQAQAEIDAATGLRMPGVGDRSKLQYTEAVLYE

TLRLGSVAPITLPHKTLCDTTVGGYEVPKDTMVIVNLWAVSQDPKLWDQPATFNPERFLD

DQGKLKMTKPGWLPFSAGRRACLGENVAKSELLLLLACFLKRFTISLPEGDKFDPSHKLA

GMLIHVPKPYKIVVTPR*

>TRINITY_DN380742_c1_g1_i4

MEDRKNMPYIEAVMTEIMRIKTIVPLALPHQAVKEGEIKGYTIPAETIVLTNISEIHMNP

DAWSEPHVFRPERFLDQNGKFCPPKENFLPFSIGPRVCLGEMLARMEMFILFTRLLQCFT

FTSVPGQRPDLEGHMGITFTAKLQNIVAKRR*

>TRINITY_DN380742_c2_g1_i7

MWLVTSLLTSVNLTSVVIFALVFLMGYYYLARPPNLPPGPPGLPIVGNMFSMSSSDILNK

LKQWHTQYGDVFTLRLGSKYLVVISSYKLTHEAFVKNSDVFSGRPMNLYTNQQIVSATGR

GVLLSDGSVWKALRRVTLMTLRDFGVGKKSLEQRVQEEARAVVEVIEKSQGQPMALKPLL

AKFTSNIICSVIFGNRFDYDDATFIELTSLLSETVQQPFLFNPINFVSITRFVPGVRKFL

DTILHTFVSIDKYLEKILKEHMESFDPKEIRDFVDLYIKTSQEDSRVYNARNMRRVIIDL

FAAGTETTATTLDWAVLYLVTYPDVQKRCQKCVDEVKSGWQNLLLGTLASS

>TRINITY_DN381602_c1_g1_i2

MLANFTTMSALVGAMVVAVGTAVVVRCARWLWWYRSYYNFFMKLPGPQDFSWIWGSLHMF

RNKTSQERIDLMTELTLKFPKFYRLWLGPFDTQIHLFHPSTVRELLKTSEPKPFQYRFAI

DWLGEGLLVAGGAKWARSRRLLTPAFHFDILKPYVGVGNKASEILLSKVKKYADDKKSIE

MFSNISLCTLDVLLRCAMSYANDIQTQGESHPYVQAVTELTELWAERGRNPLVYNDFIYG

LTKNGRRFKQQCRFVHDVAERVISDRQKSLESEGPPKKRYLDFLDILLTARDDSGTGLTP

LEIRSEVDTFMFEGHDTTASAVSWAMYSLCEHPQVQAKVQQEIDSVLQGRDSDNIEWADL

PKLEYLTMVIKESMRLHCPVPFISRLLTQPMQLEGFTLPTGTVCTINILNLHHNPAVWSD

PWTFRPDRFLPDNIKDKDTYAFLPFSAGPRNCIGQHFALNEQKVMLTRLLRRYTFQLDPA

YAVKRRFAAIMRTETGMRMFATPRTPTV*

>TRINITY_DN381602_c1_g1_i5

AVSWAMYSLCEHPKVQAKVQQEIDSVLQGRDSDNIEWADLSKLEYLTMVIKESMRLHCPV

PFISRLLTQPMQLEGFTLPTGTVCTINILNLHHNPAVWSDPWTFRPDRFLPDNIKDKDTY

AFLPFSAGPRNCIGQHFALNEQKVMLTRLLRRYELGYC*

>TRINITY_DN381802_c9_g8_i4

MRVVSLVPLGIDHLVNQDVELKGFHIPKGTVAFSTTYVFHKSSNLWTDPDSFIPERFLDK

EGQLLPANHPTRQSMLPFGIGKRSCPGEMFGRARIFLYVTTLLQQFDILPPVKEKLLPME

KDSYTYEFFIRLKPYHCYLRRRQFRNE*

>TRINITY_DN382010_c20_g2_i2

MGMVGPKPIPFFGNTKTLKEKGMFGAQLEWGQKYGKVYGMYFMRQPVLMTTHIDILKEVF

VKDFNNFLERNLASASRRYPLKYALSSVGGDTWRRQRHTMTPAFSASKLKLMGKFISRCC

DNLVAAVHTLTNEGKPMDVKQMFGALTLDVIAGTSFGLDTNSLADQKQSVLLQSVVRMMA

SSDTISPVRSFLALFPALAPIVYALGFRESYATAKETDFIAHTIRGLIEERKSDGNKGRP

DLLQMLLDAESTEAEVKANPQDKRLTPVEVVAQGVLFFVAAYETTASALQYLTYLLAVNP

EKQDKLYKDIVSTLSNEQPSYENVTSIRYLDNCIYEALRMFPPVPLAGRKALKTCTIKDV

TIPAGSDVFVCIYQLMRNEDLFPEAEKFIPERYDDNTHPTPAFVKEMMFGAGPRQCIGLR

LAMYEIKMVMIHLLTRFKFVRIPQTPETITFKQTMSLTIPDKRLLVGTECRLQSS*

>TRINITY_DN382181_c4_g1_i2

MNRSVLEKLCRQLAARARDTTRLASTRHKATRATANSPALTSEAWDSDVMEREVSQCPMT

SAPETAMEGLQHQKRALSTEAQAANYSMSQDITLPEESIADTPTSAYQPSTAVPSYLDFH

AARAFSEMPAPLSLPIIGTMWMHMPGGPLHGMTFEQKTKKMQEMYGSIFRETVMLGFTMV

HVHRPEDLEAVYRVQGSRPRRDAFRMLHKYNTQFNDGVQGLLTSQGDAWHKLRSQAQVKM

MKPKSASAYLHLHNGVADDFASTIHRLRDQDGVIPDLLPELYKFAMEGIGCVCFNRRLGA

LDTNIPADSDTFRFIQAVSDVMEASHNELQTLFLHRYSASFKKLVQAQSFIRQLSIQESM

RTLEDFARRDRDLDGESGDLIPYLMTKTELTEQQVLTLITEFFFAGVDTTSHLLGFALYC

LAKNPQAQDILMEEIDRQVGDNDIITASTLGRMSYLKAVTKETVRMMPITPGNGRTLLRD

VVLSGYHVPAGMNVGMHHDWTGKSEQYVKEPDRFEPKRWLRGSTSPLGDIHPFVVIPFGF

GPRSCVGRRFAEQEFAVALIKILQKYTVEYAHTEDLWYEMCIVNKPMTPLTFKFVPRN*

>TRINITY_DN382353_c1_g1_i1

MSELLASLQSGYQHAMQALVHLNNRAPGSLTTQALAVGGVAGMLVYILLNRRRRLPPGPR

PLPVLGNLLWMRSEEEAFYVTLARWAQEKYGPVVTIYLGPMPCVTLNTIDVITEALVHKG

ADFAGRPFLHSMHVLTEGSKDILFASDSAAWKLQRKIAVQALRHYLRGHHLEKILHKAIS

LNVNKMLAEPGPFDPHPYNTAMIFRIINTICFGEEESLDDDATQYVMTLFDKFNEEFGNG

VLEDVIPFLKYWPTQKFKNLTSSFREFLQHIHKNIERHRKTFSPDNIRDLTDSVLLAQSE

AARKESSEIMALLTDTHVGQTVSDVFAGGWDTSRLTMDWILLFLAGHPEVQKRVQREVEA

VTEGNSLPGLKDREHLQYTQAVLYETLRLGTVVPTLLPHSTVCDTSVGGYHLSKGTMVLV

NAWAVLHDPDTWAEPEVFKPERFLDEHGKVKSKLDSWIPFSLGPRACLGESIAKPELLLL

LAGLLKKVDITLPPGTEYSQDYLPTAGMAVHIPKPYKVVVTARQSAASMNDVTEQRHSTT

AVNDVTEH*

>TRINITY_DN382353_c1_g1_i3

MSELLASLQSGYQHAMQALVHLNNRAPGSLTTQALAVGGVAGMLVYILLNRRRRLPPGPR

PLPVLGNLLWMRSEEEAFYVTLARWAQEKYGPVVTIYLGPMPCVTLNTIDVITEALVHKG

ADFAGRPFLHSMHVLTEGSKDILFASDSAAWKLQRKIAVQALRHYLRGHHLEKILHKAIS

LNVNKMLAEPGPFDPHPYNTAMIFRIINTICFGEEESLDDDATQYVMTLFDKFNEEFGNG

VLEDVIPFLKYWPTQKFKNLTSSFREFLQHIHKNIERHRKTFSPDNIRDLTDSVLLAQSE

AARKESSEIMALLTDTHVGQTVSDVFAGGWDTSRLTMDWILLFLAGHPEVL*

>TRINITY_DN383085_c5_g9_i3

MSALLKMAQDYCHHAITALTDLSNHAPGTVATQALVVGALMGFLAFQLMKKRYRLPPGPW

PLPLLGNLLNMRGKEQFYITMYRWAKEKYGPVISIYFGPVFCVTLNDFTSINEALVTKGS

DFAGRPMLHSIDVLSEGSKDIAFANYSPAWKLHRKIAMRAIRHYMTGSHIEKVAHQVLTL

VADKMAMESGPFDPHPYNSTLMFHVINCICFGDAKKFDDSSIQKLIHTFDQFTKLMGNGF

FEDIIPFLKYCPTRRFRKVQGLNSEFLNHVQTNIEKHRQTFSPDHVRDITDSILLAQMEA

AREESAEVMAMFTDVHVRQTIADLFGAGVDTSRITLDWTLLFMAGHPEIQKKAQAEIDLA

TGPGGRMPGMGDRGKLQYTEAVLYESMRMGCAVPTALPHKTLCNTTVGGYDVPKDTMVLL

NIWAVTHDPALWDQPDTFRPERFLDDKGKVKIPKPDGWLPFSAGRRVCLGESVARPELLL

LLACLLQRFTISLPPGQQYNPTHQVASIAAHIPVPYKVIATPR*

>TRINITY_DN383192_c2_g2_i3

MSELLKTVQDSCHHTITVLTDLSDRTPGTMATQTLAVGALVGFLAFRLMKKRYRLPPGPR

PLPMLGNLLSMRGKEQFYITLYRWAKENYGPVISVYLGPVFCVTLNDYSSINEALVLKGN

DFAGRPTLFSLHTFSEGSKDIAFTNYSPAWKLHRKIAMRAIRHYMTGSLIEKVVHQVLTL

VADKMAQESGPFDPHPYNSTLMFHIIDCICFGNVKKFDDPSLKRLIHIFDQIAKEPGNGF

LEDIIPFLKYCPTRKFRRFHALNSEFRDYVRINIEKHRQTFSPDHVRDITDSILLAQMEA

AREESAEVMAMFTDVHVRQTINDVFGAGVDTSRNTLDWALLFMAGHPQIQKKAQAEIDLA

TGPGGRMPGMGDRGKLQ

>TRINITY_DN383192_c2_g2_i6

MSALLKMAQDYCHHAITALTDLSNHAPGTVATQALVVGALMGFLAFQLMKKRYRLPPGPW

PLPLLGNLLNMRGKEQFYITMYRWAKEKYGPVISIYFGPVFCVTLNDFTSINEALVTKGS

DFAGRPMLHSIDVLSEGSKDIAFANYSPAWKLHRKIAMRAIRHYMTGSHIEKVAHQVLTL

VADKMAMESGPFDPHPYNSTLMLHVINCICFGDAKKFDDSSIQKLIHTFDQFTKLTGNGF

FEDIIPFLKYCPTGRFRKVQGLNSEFLNHVQTNIEKHRQTFSPDHVRDITDSILLAQMEA

AREESAEVMAMFTDVHVRQTIADLFGAGVDTSRITLDWTLLFMAGHPEIQKKAQAEIDLA

TGPGGRMPGMGDRGKLQYTEAVLYESMRMGCPVPMALPHKTLCDTTVGGYDVPKDTMVLV

NLWAVTHDPALWDQPHTFRPERFLDDKGKVKTRKPDGWLPFSAGRRVCLGESVARPELLL

LLACLLQRFNISLPPGHQYSPTHQEAGIAAHSPVPYQVIVTPR*

>TRINITY_DN383776_c2_g4_i4

RYRHVMTPAFTTGKLKALEGYIIQCSKVLCQVLRDKADKQEDIDLKAIFQRFTTDVIAGT

AFGLDTNLQRGEDAETHDMIKAMKGILSNLSSRSIILIAMAFPFMAPLVRAMGYKMFSPK

HLGFFKQCITTIIRERDQNPEEAKKHVDFLQQMLDIRVAPGEEVTDLDDASFGTKTNKKL

TEEEVVAQCILVFNAGFETTSSSLRFLSYCLATNPDIQEKLYQEIQEVIGDGELKYEQLQ

ELKYMNLVVMEALRMYPVVTSIGRIATETVNLKGYRIQKGMPILIPIYVATHDPEIYPDP

NAFKPERFADGKDSLMAALPFGVGPRQCIGMRLALQEVKTAVVYV

>TRINITY_DN392296_c0_g1_i1

TEIGGHKIPKNTAVIQALGVVMQDEKLFPVPNKFDPDRFSVENSKERDTLAFCPFGFASR

RHCPAKDLTYMMASALVAILIRKFSVSLVEGQVVTPFYGLVTKPEDDVWVTVQKRK*

>TRINITY_DN422106_c0_g1_i1

PKDTLILVNLWSLARDENIFEDPERFYPDRFLNSQGQVNRELAEKFLPYSAGRRRCPGEQ

LARMELFIFLTCILQKCCIAVPEGQHPVVDSKYGLTLKPLDFEVCASPRV*

>TRINITY_DN473880_c0_g1_i1

CIKEAMRNYSTIPNIDRKIVEPLNIDGHMIPAGTEVGVSLWCLHHNQTVWDRPYDYLPGR

FLGDNVTKIDPFQFWPFSAGPRNCIGQNFAMHELKVVVARIFHRFNLSVDPDHEILCAPL

ATFKAEKDIKLFVTPRQH*

>TRINITY_DN534404_c0_g1_i1

DFTCFGESKPYDDPSIHKIIDLFDMVAHEKGNGFFEDFIPLVKYWPTKKFRKFVAAFQEF

WDYLETNIQKHRDRFSPDSVSDITDSLLLAQAEAAREESTEVMAVLTDVNLRETISDLFG

AGVDTSRITLDWAML*

>TRINITY_DN11675_c0_g2_i1

MTSPPRRQTFLTLTLITLLLSGALARSLRDENMQNVLRGIMAEDEAEPSEFDTLLHRLLI

PVDEYTPRSSAAESVHLKRSWADDDKRVFNVPEPMKRKMFWTPLGHLPASARMGRPQAGL

RPHMEVSGSSVFRYG*

>TRINITY_DN131742_c0_g1_i1

MQRTTEVLLVVCLMMTLTSFLQQTTAAPSWRPQGRFGKRGDAAPFVESGKPTGLSGEIWV

PVEAMATNERAESLRVVAKLCTLTGVAGFSPCSWTNDNASETY*

>TRINITY_DN136837_c0_g1_i1

LAVPRCCCRCCFSCRCLLPGAYSASWFARHLSDSRLLLFEVTSDTFCLEEMSARLVCVIL

ALCLCLTASHTQGTARRSSLNRLVGQQPLLFGRRGINPNMNSLFFGKRAGLTGPQPSVED

IRSACTMLMTAYEQVAVVDDNES*

>TRINITY_DN148273_c0_g1_i1

MEKFLTAVFALYLLTAAVLATDKEVLESAEHVKRPFDSISGNSRLSAFAKRPFDSVNGNS

RLSAFAKRPFDSVSGNSGLSSFAKRPFDSISGNSRLSAFAKRPFDSVSGNSGLSSFAKRP

FDSVSGNSGLSSFAKRPFDSV

>TRINITY_DN174926_c0_g1_i1

MTWTGIFANHTVLLLMTTATLVSPFLFLDKAPPSPDFFGADDTEQDDLRGLVAAADNPYY

SRQSTLMMAASSPRIGLRSSLIGSSDTCHVDPVKMSIRPPRRYAGLCAPTTEISYGCRGG

CPSYSRVDARNATSVLRSCSCCRPTRFGFRMVKMKCAGFALRTTVKFALGCHCRPCMANV

ASVDIHRLRDLLRESSIANIG*

>TRINITY_DN184774_c0_g1_i1

MTTMAFLLPILAAFFFLGLSSVQGLPTSKASHVTSGVAPERGLHKVTARSSGVKALTKRD

ISLNQDLKSLANMLLAREYDRILSNRMNREFLRKIGKRGSSSLVGGFEDVMDLLPGEPQE

DSLPSWWKCSDCDEENKFGRNSAPSSPPPSPRNRWGLAGSP*

>TRINITY_DN187635_c0_g1_i1

RGFDDLGGFNVHGYKRGFDDLGGFNVHGYKRGFDDLGGFNVHGYKRGLDKLGGTTTNQIK

RGFDNLGGFNVHGYKRGFDNLGGFNVHGYKRGLDNLGGTSVHSVKRSSESGDDKRGLDNL

GGTEVHGFKRGFDDLGGFNVHGYKRGFDDLGGFNVHGYKRGFDDLGGFNVHGYKRGFDDL

GGFNVHGYKRGFDDLGGFNVHGYKRGFDDLGGFNVHGYKRGFDDLGGFNVHGYKRGFDDL

GGFNVHGYKRGF

>TRINITY_DN188816_c0_g1_i1

DVTTDLSRHGADSVTFGQSRKNKGDGIHPWNTPISHYSNRNMGLSTGDVLKTHHPQENKG

FVDSINGGILKRSYPPKVKRYIDSIAGSLLKKSDPRKEKRFIDSIAGDLLKKKSYPRENK

RFTDPIAGTLLKKSHLQEDKRFIDSIAGDLLKKSHPSEDKRYIDSIAGELLKKSKTQKNK

RYIDVIAGNLLKRSDPQEDKRFIDSIAGELLKKFYPQNDKRYFDSIAGNLLKKSQSPQSK

RYLDTIAGDLLKKSVPQENQRYDGWNHQWNQHYTDSVKGYPLKRNGNRPDKRYIDSIAGD

LLKRDSIPDRKDPDEHRMDPVLEDLQLIQDRKENQRYLDSLGDALVSGDDTDKRFLDWIA

GHLLKKHVSHLVKDPSQKHDADSNTGKQVEKNGVSNLTIDFTKD

>TRINITY_DN196626_c0_g1_i1

STLLWLKRNRREILVNAGLNQQLRMSKYRELEQLKEQERTLMQLHMNFNDQLNRLKVEEL

ALTNLLRLQKEREMQAQTGSTDKVGPAEEAIEEETMQVLNLLVSDNPHGRGAEEEEEEEE

EEEEDEEEESELVKGDGE

>TRINITY_DN202052_c0_g1_i1

GGGGTLKATRSQLEGSLAKLACVDTTDRPLFCLFFTPSTNPALSLTHLLPLSHLTSNSSK

QPHHHQHCWSVGRTLCVLSIHGRLLTHHPSQPGPSRKRRRPVQSSFRHPQANSSKALFRL

LLAATYLTT*

>TRINITY_DN210274_c0_g1_i1

MDKLMTAIFVCCLMTSSAAANENEVDERAVDTQRQQRSSARPFFPTNPAAASLRGLGKAS

LESNPTEPINALSKNKRPLDSTSRYRGQGSLAKRSVNSFSEDSQIDSSAKRSFDSISRTG

VMGSFGKRSFDSISGVGSMGSFGGKRSFDPISG

>TRINITY_DN21757_c0_g1_i1

MVALLAAVILLALLGTVQGQNYHYSNGWHPGKRGNGALNKATFYRSITTENLCRYKPRVL

SVINKLIQEEIARMERSCSSEEPINHLKLLMEGSPFHSAEATRQKLEEAVAEDEDEAMNK

W*

>TRINITY_DN242745_c0_g1_i1

RLPSLVICTKSSLEDELSFFVAWITLHSIMCKNEAELTWRRAAGSSLCLLSLLLYGALLS

SFLFPPSRAQSGGDIIQAFANRFRQASREEFLEIWHSDCHRRCRAQLVSHVNLACTFDPY

KMTKRSLTKREVNSTVHHLVKDLAATPESPFLVKTTASSFLNKVSKGKSSRAHKRTKRGI

MNECCYSKSCSWEEYAEYCHTYNRRASLRDTHCLS*

>TRINITY_DN247584_c0_g1_i1

DLSEDKRFMRFGKRFMRFGRNPEDGQDSTDDEATQKDKKFMRFGKRFMRFGRGGQDEGDL

SEDKRFMRFGKRFMRFGREPMEAEKRFMRFGRSSEDAEGTKAEDKRFMRFGKRFMRFGKS

SSGEASESKSEQPSS*

>TRINITY_DN251387_c0_g1_i1

MREVRSVLVALLCMLSQVTGMPEYTCGVDARPHLEGLCGPRLTRARDNLCFLLTADFPEY

FGKRSVKDFFNRPVKDFVDMGILADEMTEAPMDQKESKLYNSWSAVRSLSKRIEPMSHIQ

KRGMVCDCCYNKCLPSVLAQYC*

>TRINITY_DN251761_c0_g1_i1

MSTRFEPACFAISLLLLSMTAALGHADVSTDKQHKLQKRDSPSEPLADSEVEKRAAMDTN

AFFGKLGKREVGQDSDLDELDKREMDTNAFFGRLGKRQMDTSRMFGRLGKRAGMDSFGFL

PRLGKRGMDRYGFWGKLGKRAGVDTYGFWGKLGKRAGVDVYGPSDEELVKRGMDKYGFWG

KL

>TRINITY_DN252481_c0_g1_i1

MDNLAFMPQLGKKDMDSLAFMPSLTKTVKATPNKLNNETTKPEVRDETDRKENSEDNAAR

SKRSLHDFLPFPLFDNRQFSSKRRIDNTMFGPQLGKRGMDSRLYRPQLGRRGMDDWMFGP

QLGRRGMDNTMFGPQLGKRGLDDRMFGPQLGKRGMDDRMFGPQLGKRGMDDTMFAPQLGK

REMDDRMFGPQLGKRGMDDRMFGPQLGKRGMDNTMFAPQLGKRGIDDRMFGPQLGKRQRM

DNLMFGGRLGKRSAADLLRDLESLWVLD

>TRINITY_DN262681_c0_g1_i1

PQNMYSDEDDSSSNIINNDGNRDDNNDIVKRVFCNGFTGCGGRHRELSRRRRIFGKRLIP

VLVKRPFCNNFGCFNSKRNPLAAPSVEGFRARLLSQGFAPAAEGETPGGKQGGVYKGKRL

FCNGYGGCRGGKRSLFSPWMSKMSSVADSLR*

>TRINITY_DN273830_c0_g1_i1

MLAPKGLACITQLLLLLFSFVLITADSEIPDSSNVEKRSVPLFGNHFLYKRLLKLPYQRR

QYRSRYEHSFIGKRSVDSDLENVDLDDLLDELEDLQDNNYDKRVPSFGHRFVGKRIPTGL

GIRAPTFGHRFVGKRQPMFSHRFVGKRVPDGIDERAPSFGHRFVGKRAPAGLQNRAPTFG

HRFVGKRVPDGFAVRAPTFGHRFVGKRYPDGFAARAPTFGHRFVGKRDEDDEFNDYESSD

YYHTIRKRFAVDNYHMEKRAPSFGHRFVGKRLPGGISERAPSFGHRFVGKRIPNGLDTRA

PSFGHRFVGKRQPTFGHRFVGKRIPDGLEERAPSFGHRFVGKRIPDGIDERAPSFGH

>TRINITY_DN274156_c0_g1_i1

TWKFLASATDRALFLKTLQEDTSPPASSPPKSCPPPILTNTFESIPSYLSVVMVRSSAVL

VLLVLAAVVCTSHANWYGKRGDRADFFGLLMQQRLDGLTSRDMNAEVALAAIDQIIQIYR

QHRTQSAMELKQGA*

>TRINITY_DN292906_c0_g1_i1

DSVSGNSGLSSFAKRPFDSVSGNSGLSSFAKRPFDSVSGNSGLSAFAKKDVGDDDDDIVS

EENDVDEEGKRPFDSIHHGGFSSFSKRSVDSVDHDGKLSKRSTDEEKVDEKEE*

>TRINITY_DN295089_c0_g1_i1

KRRFDSIAPSSSLAGFGKRRFDSIAPSSSLAGFGKRAMDSIAPYSHFGEFGKRGLDSIIA

YSPGEEEAELDTLAALNHQLLQMQRESEAQEDVSRLVNNLAGLSSLQTPWHGEDSASDAS

LPDPSGLSLDQEQEERKRSHDPIAHYGRFGRFGKRGGEDEEGEDVAFFPITPSWRQDVEK

LCGPLSPSALSEDGPRTPISKRGFDRIATSGFARFGRLSWTPSSAPASNPTSIPTVPPPS

TRVDKSYLPFCQIAVRLQQYLDTFQRQVRANSHRRSKRELDLSSYGRLATHGQK*

>TRINITY_DN296355_c0_g1_i1

MMKKILLSLTLCFLFQLHRANSEEASAAAETDNDSSSNSGEELSRLKRNAMDMRRLGRGI

QMLRLGKRGVPMLRLGRSSPEDTLSLEDLLNAMEESQYLDDFYPYPLPEEPVHGRFRRSA

DHSQESVPADSVFADGANKSAGDVKRSVDETAEPLEEEESYFNEQGDGDDIEKRPMSMLR

LGKRPMSMLRLGKRPMSMLRLGKRPMSMLRLGKRPMSMLRLGKRPMS

>TRINITY_DN297061_c1_g1_i1

MATLGLCSSLLMLLLLALNVLWVQGGYEHTCTLATRRQGSHRAGICGSRLPKVIQQVCRV

MGRGYAGATVEGRKRSARLQKLSRTTRDASFDENLRDVLLNKREALSYLKKRSTRLSTRG

RLGRFGTQGITCECCYNQCSLSELLQYCN*

>TRINITY_DN297615_c0_g1_i1

MELSQVTHAMVVVLFCLLVAAVWCEEQDQGSALDDLAPSQPQKRSIADLPSDDSAYPAVY

VDDDDLDKRSSLFRFGKRGSLFRFGKKRSSLFRFGKRSSLFRFGKRGSLFRFGKRDGDLD

DEWVMYPDVYIPDDGVKRTVKSFHWGRQTEE*

>TRINITY_DN299316_c0_g1_i1

MQGIGQLLASVLLLLPLLSVLAHNTDDSNPSHVSKRSTGDAAEGQSTWQGMDDVTAAKRF

NEFVGKRAQGFLPAYKRFQIASKRNPYEFIGKRPNRFMAKRTASNFLSKTGKTLTKRSPQ

YEFVGKRLSPPSAHKRALIDFYGKRVQLPYEFVGKRIFGSAWERVSANTVEGDALIKDVT

DVLNTRDTADHDSPALRKKRYSEWLGKRGAPIADQLLRDLVNKRISAMMRNRLQNGANPE

FIGRREDDYPEDLDVSKRYTEFIGK*

>TRINITY_DN299316_c0_g1_i2

MQGIGQLLASVLLLLPLLSVLAHNTGTDANSSQLDSKGHVASSAVTSPSPSGGSNGAADN

AESTASAPSNPLPPQHPDLNPPFVISESGESLVPAASSLTYQLPMTVLLARAPLQAPHRP

YVTLLGPGDEEYAASLIQEPQANKVQDNEITEHREPEDIEAIDQVGSNDNEVTDDFDQTD

DKLSDEEHPNQKLRVKRGGYTAPYFVGKRSLEESEEIEQEELQKRDGDDYGSDEQKEAEN

QEDDAAEKRASPMFIGKRRTPMFVGKRGPAPM

>TRINITY_DN299316_c1_g1_i1

PMFVGKRAPAPMFVGKRRTPMFVGKRAPAPMFVGKRRTPMFVGKRAPAPMFVGKRSATSV

QDDAQLLEEVSSPETSNDLPDELQSSVNPEELEEQSSSESPEKRRAPMFIGKRRTPM

>TRINITY_DN299316_c2_g1_i1

TPMFVGKRRTPMFVGKRRTPMFVGKRRTPMFVGKRRTPMFVGKRRTPMFVGKRQEENLNK

LLSALQTLQAARHYRRMIQADKRHNAPFFVGRRSSSSSEDWTQTAPEITDAYVR*

>TRINITY_DN302254_c0_g1_i2

KRPMSMLRLGKRPMSMLRLGKRPMSMLRLGKRPMSMLRLGKRPMNMLRLGKRDGEDAEPI

DEEEFIEPFEEEGQVEEFPAEKRPMSMLRLGKRPMSMLRLGKRPMSMLRLGKRPMSMLRL

GKRPMSMLRLGKRPMSMLRLGKRPM

>TRINITY_DN303288_c0_g1_i1

MKSLHTLWHQQTAALLLLFCIISNASCAVFYTQSRDNDYPRIGRRAFFTGGGKGSSYPRI

GRSSPSTGTGSSTETGKANAGYGDSFHSPSKRGIFTDGEGQFPRVGRRGLAAILSQSPEL

SAADTLMTSLEIPDVLDTQEDVARTPLRLPLGLLFFSFDTNGDKVLSREEFVEGMSSARE

DGTVCR*

>TRINITY_DN304580_c0_g1_i1

GRFAKELSPLFPETESKGRFAKELSPLFPEIESKGRLAKELRPLFPDTESKGRFAKALNL

LLPETESKGRLANELKPLFPEIESKGRLAKELKPLFPETESKGRFAKELSPLFPETESKG

RLAKELRPLFPEIESKGRFAKELNPLLPETESKGRFAKELSPLFPEIESKGRFAKELRPL

>TRINITY_DN304580_c0_g1_i3

GRFAKELSPLFPETESKGRFAKELSPLFPEIESKGRLAKELRPLFPETESKGRLAKELRP

LFPEIESKGRFAKELKPLLPEIESKGRFAKELSPLFPEIESKGRLAKELRPLLPETES

>TRINITY_DN304957_c0_g2_i1

ALLPPRGWPPAPPQQPLRHHHPCLHTHTHTHARAELPPPEVSSDHVLDTNNEKAELLRQN

PKFSSAEMNPLQQMFALGLLAQLTLLCCHPGVLGEEEAAQELVRDPAQSQSQGLRQKRAP

GWGKRSGDLDTFDSDATDDLLDSVDAESQADKRAPGWGKRDFETEKRAPGWGKRAPGWGK

RAPGWGKRAPGWGKRAPGWGKRAPGWGKRAPGWGKRAADWMSSGLEEKRAPGWGKRAPGW

GKRAPGWGKRAPGWGKRAVAGGLDSGECQLLAEEVDELTLRAAKAESQLQLLCGSQPDMT

EAARRK*

>TRINITY_DN305337_c0_g1_i1

MCRHEAVTWRIAAASSVCLTTVVLGLLMATLLLQPVQSTSDRNIIQAFTERFLRTSGEEF

YELWHSDCHRRCRAQLISHVTLACTFDPYKLTKRTLSKRDVNNTIEHIIKDAEHTTVSPF

LAKPAATTFLHKVTGGKSNAHRRTKRGIMNECCYSKSCSWEEYAEFCHTYNRRPAIRDTS

CFP*

>TRINITY_DN305985_c0_g1_i1

MKTSAILCYTVVLVCVLSGPVVALYSGIDCLSDCLQYGASLFCYCSDLDLQDLSSADSVK

RGGAFPFRYGKRDLGKRQIPFRYGKRSVLNVKRPKIPFRYGKRSVSNSAAAPLAYNPQRA

SAYNHMSSMMAEEK*

>TRINITY_DN305985_c0_g2_i1

ARAVLPTLSTHPATRTPNIKRILDTNNMKTSAILCYTVVLVCVLSGPVVALYSGIDCLSD

CLQYGASLFCYCSDLDLQDLSSADSVKRGGAFPFRYGKRDLGKRQIPFRYGKRSVLNVKR

PKIPFRYGKRSVSNSAAAPLAYNPQRASAYNHMSSMMAEEK*

>TRINITY_DN308467_c0_g1_i1

RNENSGFYKRPLNKMDGLYKRVLHENNAFYKDMLNANAFNKRAQNENIDFHKRDSKETST

FYKRSADRNERLLSAPREYFSTFYKREFPRFQRILQPPPR

>TRINITY_DN313143_c0_g2_i1

MGRDAATELQPRQGGSPSYSYSSTVTMVMTLTLTLTFTLTLHSAGAQGNDNCQLIPVREN

IIKETTVLHLNRQVQASCSAEVNLHKCEGQCESKVIPSVRHARGFRRDCRCCKEGTIRTR

TVPLTRCFHNGELLVGVMDMAEVSDLESCSCVSCMN*

>TRINITY_DN314851_c0_g2_i1

MMSSITMVPMGVCRLWVLAQVCWVGVLLMLPLSACHGPLSELDTCSSPSSVCSEVLSGES

PQRRCTCSEGKECKARFRVTDKLWFGTCGPIEELPLCERHQTAVETSVDKSAIKVVCRCP

YDQRWEDETTFLHNLSCRGPQQDNNNNNKNREGGQDDKERAGLTRRDVSQMEEPLSQDSF

RHTLQQLTDTGDQGSNEDLGTTEDENDDEEEDFLTSTTGNQLTKSEEEEEEEEEEKEEEK

RDHLS

>TRINITY_DN314943_c0_g1_i1

MSSALAFITALTLLLMSSQTMSASSSSGQKPEASSLQGPQLQDLMSEDDARLLKRSLREE

LQQHFELLQEAEENILQKIEALRAQRQAISQRKRSHYMCLVNIVACYK*

>TRINITY_DN319448_c0_g1_i2

MSTYSTCVTFAIILVLSQALCFRLILTLPLHAPNLWLAPPSRFFWGPPYNGPARSYWPTS

GNDNQVAAIRRSHHLIHQDDDDVEDFAGPERRAFEEIGSGLIKRSSSAFRSFMDNEDNYG

DVPDKRQLDQVGMGLIKRPFDRVGAGLVKRGIDSIGAGLIKRPIDRIGAGLVKRPIDHIG

AGLVKRPIDHI

>TRINITY_DN321601_c2_g1_i1

MADKAKRFDRIGQSSFRSFSKREDIAETSDDKAVESSDVAPSPVTPAPSSGNGEASKHIS

KRDTSSMLEADKRRFDSIAPSSNMAGFGKRRFDSIAPSSQLAGFGKRRFDSIAPSSSLAG

>TRINITY_DN325892_c0_g1_i1

MVSRGHILYVILLPVLARVAADDADEQIMSEMMSGDHDGASNSNSLVRSSEHSSLPRSLI

LQMLRSGSLRGAASSAPLPAPPAQPLLSRLADKAEYGQDGEVTKRVFCNGFTGCGGRHRD

RSRRQERYGKRLIPVLVKRPFCNSFGCYNGKRSSSSFSIVDPVAAYRARVFAQMMAAAES

ARRGEEGERPAGVVVPGKRLFCNGYGGCRGGKRSLFSPWMSKLNGVVGEGM*

>TRINITY_DN326225_c0_g1_i1

MTSLHHFLSASAILLLVTSVLARSLKDETLQKEAALMDATSTGNDPYSDPRPLADLYDLL

LTYRHALPDDQSALRMGEMKRSWSEDGFLVPSGMKRKMFWTPLGHLPASARLGRPQALRP

NMEDSGSPVFRYG*

>TRINITY_DN327687_c0_g2_i1

MTSYYRYLLTLAIVIAAVKLLLADDLDFNDDASFALGEDFEPFGDIDFGKRGFGNKRGFG

DKRGFGDKRGFADKRGFGDKRGFADKRGFGDKRGRSYSPSNLMAALFRSYYHRQPLAGSI

ALKRLLEKQGIWQ*

>TRINITY_DN330612_c0_g1_i1

MYRQSPCPHRHHLMLVIIILSSLSLLIPAAAEDVTSTTAESPAANPKQTSTEEEEKGADV

KDDVTKRGFDRINGHSSFAKFGKREDGASQLQDQKRRFDRISAATDFGTFGKRKEDSDSE

VDKRKFDRIAGASGFGRFGKREQQETDSDEQKRRFDRISGASDFGRFGKREESETLSDMD

KRKFDRIAGVSSFGRFGKREEDETAQSDKRRFDRISGASDFGRFGKRADGEEEDTVEDQE

DADVNKRTFDRIAGVTNFGRFGKREDREEEEEMDKRRFDRISSATDFGSFGKREEDDEDS

MTDKRNFDRIAGISSFGKFGKRQEQDESMDKRRFDRIASASGFAKFGKRQSEQATEGLSI

DNDKRKFDRISGLSSFRNFGKRTSDDGLTEDKRFDRINGYSSFAGFGKRGFDRISGMSSF

AKFGKRGFDKIGGLSSFA

>TRINITY_DN332670_c0_g1_i2

MQGYGMKFSVVPVVLFMLLASSYGCFIRNCPRGGKRAFDGGKPCMPCGPDGAGQCVGPAV

CCGKSFGCLVGTREARECEKENESSTACSVQGRQCGRDNSGRCVAKGICCVADACSFNER

CAEIERNGRDELLGLIRRLLVTHQYE*

>TRINITY_DN332670_c0_g1_i3

MQGYGMKFSVVPVVLFMLLASSYGCFIRNCPRGGKRAFDGGKPCMPCGPDGAGQCVGPAV

CCGKSFGCLVGTREARECEKENESSTACSVQGRQCGRDNSGRCVAKGICCVADACSFNER

CAEIERNGRDELLGLIRRLLVTHQYE*

>TRINITY_DN333077_c0_g2_i1

AQTRIMVSASHISSLALLAAVPLLLMLVGMAQGQNYHYSNGWHPGKRGGPRGSSSDGGLL

YGTLLNSGAGEEGSCRFETHVLTLVNRLVMRELSRLERTCHATDTINSLKVLLEPQSRSM

DLRSALGEEEKW*

>TRINITY_DN337273_c0_g2_i1

MKKVLSLAVCVLLHLTQVCGEDASSDKKTQTSSSSLLADASSPTELSRTKRAMDMRRLAR

GIQMLRLGKRSLPLLRLGKRYDPEDEEATLQDLLSFLDSQYYDGMDYPMYDEPLHSRYRR

SADSSSHEAASSQAEDSSSGTVKRSVDYLPSLQEDFVDEDGYYGGDQDFPEMVEKRPMSM

LRLGKRPMNMLRLGKRPMNMLRLGKRPMNMLRLGKRDGDDEELVYPDNVFEPLMEDEDDL

SA

>TRINITY_DN338314_c0_g1_i1

MAVLRSQSALLALCAAFMFISTALTTTAATAASTDEKKEEESGQEDKRAFGELANGLVGK

RGQKSKQYNYRFANKRPFGELASGLVGKRFGEIANGLVGKRPFGELASGLVGKRPFGELA

SGLVGKRPFGEL

>TRINITY_DN338825_c0_g1_i1

MAVTSSSTGLLLLLLSVILAASVVSARRIDCTRFVFAPRCRGVAAKRGGHSLSVEDTTEL

DDTFTDSVLGMGEANRDEDSEKFLRLLLDLSRAIRQPITPSARGKELLERLLRS*

>TRINITY_DN339852_c1_g1_i1

EGQSFLIFYICEDAPGTRGHNPTGGVQRLQGGNPVPAVREHNLVTTRRIVSCPPWCRAST

GTSTSPLITGIVAEDVTSPLFPKRPWGLQEGAAVVCWMVEVNTSMHITTTTAWTTVVCML

SGKAGGRDQAQWKENNQWRERAKMVLLVPALSSESVRSWP

>TRINITY_DN339852_c1_g2_i1

EGQSFLIFYICEDAPGTRGHNPTGGVQRLQGGNPVPAVREHNLVTSRRVISCLPWLRAST

GTSTSPLTAGIAAEDVTCPLFPKRPWGLQEGAAVVCWMVEVNTSMHITTTTAWTTVVCML

SGKARGERPTQWKENTRRERQQRRFWFLPCLPSQSEVGQLLLRLLGKAKSNVEVGVHILA

FKYTRVL*

>TRINITY_DN341012_c0_g2_i1

RLRDFVGKRSDEFEMSPYDKRLRYFVGKRTAEPAPVEFHKRLRDFVGKRDSFIPMGDGMA

DMTGMDVSGYYPYDVQKRLRYFVGKRLGASEIPLDNPDKRLRYLVGKRPSDVMDEFQNSP

QKRLRDFVGKRSGSGDSFSELEGSPDKRLRDFVGKRSIDVAKRIREFVGKRSGPFIPADP

ATLWTEIPAGTDLGEGGEVLDFNPVDVDEELAARVDRQLQDFVRKRSGVDKRLREFVGKR

NNMDKRLRYFVGKRMQPLTSQGGDSETFSPSKRLRDFVGK*

>TRINITY_DN341042_c0_g1_i1

MKTATGRMVLALLFTMATVTQAMNYLALPRMGRSAYIAFPRLGRGYLAFPRLGRSQGGSD

ANKDGTDCCMTGLNKEWMPQEGGTTTTRNICPAESCCQGLREILAQKPDGVFYSMCIPAC

ASEGDSKATETSRNVLRKLKDILQN*

>TRINITY_DN341578_c0_g1_i1

MKSAAFLAVMVTISLLCIPDVTLAFIRKSRAFKNDNFERLPYKRSGDDYQEDAMRLSAAN

LLPPLLNGGSEGYITVEDMADRLTQNRHLALDFILTFMDTDGDGLISAAEILPAIRRR*

>TRINITY_DN341974_c1_g1_i1

MWTARELFGMSQLFLILLSCLLTSGESENASPSDLVKRGIPLFGSHFLYKRLLRLPYQRR

QFRSRFEHSFVGKRSDAEDLDDLVDLYDDADSLEDPEVDKRYAYFTHRFVGKRVPDGLDV

RAPGFTHRFVGKRPAGFTHRFVGKRIPDGLEERAPAFTHRFVGKRIPTGLQNRARVFTHR

FVGKRVPDGFDLRAPGFSHRFVGKRIPDGIDLRAPSFTHRFVGKRVPDGIDLRAPSFTHR

FVGKRVPDGFDIRAPSFTHRFVGKRVPDGIDLRAPSFIHRFVGKRDEDDLEDFEDSDYPH

SISKRFAENSGDDTDLEKRASAFSHRFVGKRVPDGIEERAPRYGHRFVGKRIPDGLEARA

PAYSHRFVGKRQPSFAANMFSEGQDKRGPAFAHRFVGKRVPDGIDERATAFRHQFVGKRI

PDGLDKKAPGFAHRFVGKRVPDGIDERAPGFAHRFVGKRVPDGINERAPAFAHRFVGKRS

DETAQKLKSSETEVAVPSPEEMTSSPSKE*

>TRINITY_DN342115_c0_g1_i1

SSTLTSPSVSSSSSSSSAAVSVPAAKSVTLDKLPVQNNFVSSGASTHFLPVFASHAHKLP

LRAIFSNSPNHVPKNTHLPLSSTFFSDEGQDEIDLSPEKLEENSHGHHQQQNHRVKRGGY

NAPYFVGKRSLEESEDLEQEELQKRGLPNEDMVNNLPELQLQPDSVELEEEDPSVLMDKR

RAPMFVGKRRAPFFVGKRRAPFFVGKRSYIPAFGEQNPILFATRAAPMFVGRRQAKAPMF

IGRRAAAPMFVGKRAAAPMFVGKRAADDLNELLAALHTLQAARHYRRMIQADKRHNAPFF

VGRR

>TRINITY_DN344231_c0_g1_i1

MAKAAWISTLLRTALLLSILLAAVSLSCASETKSTSTSSEKPESSSHTSAKPENHKRVSM

DSNAFLGKLGKREKDASRQQGKVGNGGEPLTQQLEKRRMEQTDFEETPEKKDSVDTLGYL

GYLGKRDTTGPLSQAEQLEKSEGVDTHGQQEKRQEPYRLGKRGMDSLALMPQLGKRNMDN

LAFMPKLGKRGMDNLAFMPTLGKRGMDNMGFMPNLGKRDMDNLGFMANLGKRGMDNLGFM

ANLGKRGMDNLGFMANLGKRGMDNLGFMANLGK

>TRINITY_DN346805_c0_g1_i1

HRFVGKRIPDGIDERAPSFGHRFVGKRVPAGLDERAPSFGHRFVGKRIPDGFEERAPSFG

HRFVGKRTPDALDKRAPSFGHRFVGKRVPAGLEERAPSFGHRFVGKRIPDGIDERAPSFG

HRFVGKREQDDTDKRAPSFGHRFVGKRSEDESQENKISQTEVKVPSLAEVKPSPSEE*

>TRINITY_DN347006_c0_g1_i1

MKTDISYVMCVALISLFLLSSVVSIAEGKCAGRWAIHACWGGNGKRSGGVPAMDLESSDS

ASPFQKLLLRRPPADIPAPGSTVFVSLRPQDFLASLPTSASSSLSLSSLLSSSSSSSSRL

EKGLSGDKDDVLHPSPSDRLTSLLRMLRRLQQARDTLA*

>TRINITY_DN352406_c0_g1_i1

YKRAPSDVSAFYKRSPNDLSAFYKRAPSDVSAFYKRSPNDLSAFYKRAPSDVSGFYKRAQ

EDTSSFYKRAREENNAFYKRARDENNAFYKRGLNDLSGFYKRGREENSGFYKRAPSDLSG

FYKRARDESSSFYKRARGDTNAFYKRVHNDNDFYKRGLNDLSRFYKRARQDNSGFYKRAR

DDNSAFYKRAQDDNSAFYKRARNENEGFYKRARQENSGFYKRARNENSGFYKRSPKDISG

FYKRSPDSLSRFYKRAPQNNLSSFYKRSPGDVSAFYKRS

>TRINITY_DN352812_c0_g1_i2

MQMFTFLPLLSLFCPLILSACSRAAPTDVTKDDQSDVAGPVGQFDDVSGEGDLAKRLSSF

VRIGRPNSFVRIGRGSRFVRIGRPGNFVRIGRGYEGDLDDLGYDTEGDNYNNADKRASRF

VRIGKGSRFVRIGKSGQVDPQIKRMSSFVRIGKADPYSDLDSDDLSKRASSFVRIGRIPS

SAFVRIGRASSEDSGEAGDFGTFDRIARMGQSSFVRIGKREADPEKLAAQAHNLKQ*

>TRINITY_DN354458_c0_g1_i1

MDASACRMLLALLFVTATVTEAMNYLALPRMGRSGYIAFPRLGRGYLAFPRMGRSQGASV

DAERGASCCALGLKTEWLLAEDGKTTTQNICEAKSCCQGLQEVMDQKPDGAFYTLCIPTC

SSEETKPEAANENMLLKLKGLLRN*

>TRINITY_DN354458_c0_g1_i2

MDASACRMLLALLFVTATVTEAMNYLALPRMGRSNYFGHPFAMPRKRPSNSEVAEEKRGG

YIAFPRLGRGYLAFPRMGRSQGASVDAERGASCCALGLKTEWLLAEDGKTTTQNICEAKS

CCQGLQEVMDQKPDGAFYTLCIPTCSSEETKPEAANENMLLKLKGLLRN*

>TRINITY_DN355237_c1_g1_i1

MTSLCPSLLALAVVIATMKLLLADGLDFGDGATFELDDFDTMEDAFPKRGFGDKRGFGDK

RGFGDKRGFADKRGFGDKRGFADKRGFGDKRAQQDLYPSSALLSALFSGYHGQPLPGNSA

LKRLLEKEGVWQ*

>TRINITY_DN356503_c1_g1_i1

MDICFKMAQRDSGYAMAQRYSRVLTMVAALVVCLIVCTRVQRCDASTQASLRGHSWQSPG

CHLVGHTRVVKIPDCVPFQVTTNACRGFCISYAIPSPSRTLAYNPNHIITSRAECCGIID

THDIPVQVRCVDGVKEIVFKSARSCACSICRRE*

>TRINITY_DN357679_c0_g2_i1

AGLIKKDLDDVGAGLIKKDLDGVGAGLIKKDLDGVGAGLIKKDLDDTDKEILRKKLDSWG

PVHKKRSEDSPEHPDVKSAFNRKEPNDEFAELANKFSDDLNSKSKEKEPDDVNMAVTKKG

SDDAGTGLVKKELDDVGSGDRKSVV*

>TRINITY_DN357945_c0_g1_i1

MNLFIIPAATSLLVLLSSLSRAAPPDVTKPSDADVSRVAEPYDDVSNDSELAKRLSSFVR

IGRPSSFVRIGRGSRFVRIGRPGNFVRIGRGYEGDLDDLGYDMDGEDYDGTDKRASRFVR

IGKASRFVRIGKAGATEPQSKRMSSFVRIGKADPYGNLNSDDQSKRASSFVRIGRIPSSA

FVRIGRDPSDSSAEAAGDFGTFDRIARMGQSSFVRIGKREAEHIVHKEEEQ*

>TRINITY_DN358090_c0_g2_i1

MQVRRQTMGPWSCLWLLAVVMLETTAAYSDFCQKPANMEICYGSQSLSKRFLRFGRALTD

DPFLRFGRNMMDKRFLRFGRSDADTNLDNMIRLAIARADAEDVPLYLRKKRSTTETSPDS

PKAVASSQHPEGMAKREADDSASPIEEDKRFMRFGRSSSDNDDDMDVDKRFMRFGKRFMR

FGRGDEDDEDPMQEDKRFMRFGKRFMRFGRGGE

>TRINITY_DN360889_c0_g1_i1

MMRTSLVLLALLVIALGHALPTKNLSRQKRGFRVNSASRVAHGYGKRQFNTWDDNPLKSQ

SSSELMTVSELAQLAAENPSLTEALIEKFIDVDGDGIVSSQELFGLDAQ*

>TRINITY_DN363365_c1_g1_i1

LVGKRPFGELASGLVGKRPFGELASGLVGKRPFGELASGLVGKRPFGELASGLVGKRPFD

EVATGLVGKRPFGELASGLVGKRPFDEVATGLVGKRPFDDVANGLVGKRPFGEVASGLVG

KRPFGELANGLVGKRQFDDLASGLVGKRPFDEVATGLVGKRHFGDLANGLVGKRPFGELA

SGLVGKRPFDEVATGLVGKRPFGELASGLVGKREDSSN*

>TRINITY_DN364155_c0_g1_i1

MQMFTFLPLLSLFCPLILSACSRAAPTDVTKDDQSDVAGPVGQFDDVSGEGDLAKRLSSF

VRIGRPNSFVRIGRGSRFVRIGRPGNFVRIGRGYEGDLDDLGYDTEGDNYNNADKRASRF

VRIGKGSRFVRIGKSGQVDPQIKRMSSFVRIGKADPYSDLDSDDLSKRASSFVRIGRIPS

SAFVRIGRASSEDSGEAGDFGTFDRIARMGQSSFVRIGKREADPEKLAAQAHNLKQ*

>TRINITY_DN365731_c1_g1_i1

MRGKGHLLISTFLLFFLGVLAQNTDNQPTEFRSTEDQVDGRSPWRDVTADSMSTVSKRFN

EFVGKRAEEAGKRSYEFLGKRNRYEFIGKRPYEFLGKRNVYEFLGKRNPYEILGKRNPYE

FLGKRNPYEFLSKRNPYEFIGKRNPYEFIGKRNPYEFIGKRNPYEFIGKRNPAYEFLGKR

NMYEFLGKRDNDLAQRPFSATNKRALVDIHGKRVELPYEFVGKRVTGSAWESVEEQSLLK

DQLAAGMGQNVDKRMGQAEVDDIKSRSKRYSEWLGKRGVDTPKQLFQELANKGLSPMVLD

YAQNGVTPDFRSHNRRGLRKRYTEFVGK*

>TRINITY_DN365731_c1_g1_i2

MSTVSKRFNEFVGKRAEEAGKRSYEFLGKRNRYEFIGKRPYEFLGKRNVYEFLGKRNPYE

ILGKRNPYEFLGKRNPYEFLSKRNPYEFIGKRNPYEFIGKRNPYEFIGKRNPYEFIGKRN

PAYEFLGKRNMYEFLGKRDNDLAQRPFSATNKRALVDIHGKRVELPYEFVGKRVTGSAWE

SVEEQSLLKDQLAAGMGQNVDKRMGQAEVDDIKSRSKRYSEWLGKRGVDTPKQLFQELAN

KGLSPMVLDYAQNGVTPDFRSHNRRGLRKRYTEFVGK*

>TRINITY_DN365731_c1_g1_i4

MSTVSKRFNEFVGKRAEEAGKRSYEFLGKRNRYEFIGKRPYEFLGKRNVYEFLGKRNPYE

ILGKRNPYEFLGKRNPYEFLSKRNPYEFIGKRNPYEFIGKRNPYEFIGKRNPAYEFLGKR

NMYEFLGKRDNDLAQRPFSATNKRALVDIHGKRVELPYEFVGKRVTGSAWESVEEQSLLK

DQLAAGMGQNVDKRMGQAEVDDIKSRSKRYSEWLGKRGVDTPKQLFQELANKGLSPMVLD

YAQNGVTPDFRSHNRRGLRKRYTEFVGK*

>TRINITY_DN366555_c0_g1_i1

MAVTWQTYPVLLVLGGLLALHVCAVYGSYEHTCTLSTRSRGAHRNGICGDNLARIVSLLC

SPRGYVSNWFNKRSAPNRPDDSSVEHNLRGILLNKKEALSYLHKRVPRGTRGRSYGSQGI

TCECCYNRCTYYELLQYCN*

>TRINITY_DN367608_c0_g1_i1

MEHSQHVLMTLIVSLFCIVCVAVALPAARPEVMQGDLTHLVKLIGKLKTLDTQRHGDTAD

NWAVLSEAGNPNAGQSLSTGSASTSGSDKLSALKRGFLGFNKRQGAWSYDYGLGGGRFGK

RYYGDYGIGGGRFGRDVDHVDIADTSDATM*

>TRINITY_DN368146_c0_g1_i1

MWTARELFGMSQLFLILLSCLLTSGESENASPSDLVKRGIPLFGSHFLYKRLLRLPYQRR

QFRSRFEHSFVGKRSDAEDLDDLVDLYDDADSLEDPEVDKRYAYFTHRFVGKRVPDGLDV

RAPGFTHRFVGKRPAGFTHRFVGKRIPDGLEERAPAFTHRFVGKRIPTGLQNRARVFTHR

FVGKRVPDGFDLRAPGFSHRFVGKRIPDGIDLRAPSFTHRFVGKRVPDGIDLRAPSFTHR

FVGKRVPDGFDIRAPSFTHRFVGKRVPDGIDLRAPSFIHRFVGKRDEDDLEDFEDSDYPH

SISKRFAENSGDDTDLEKRASAFSHRFVGKRVPDGIEERAPRYGHRFVGKRIPDGLEARA

PAYSHRFVGKRQPSFAANMFSEGQDKRGPAFAHRFVGKRVPDGIDERATAFRHQFVGKRI

PDGLDKKAPGFAHRFVGKRVPDGIDERAPGFAHRFVGKRVPDGINERAPAFAHRFVGKRS

DETAQKLKSSETEVAVPSPEEMTSSPSKE*

>TRINITY_DN368481_c0_g1_i2

MAPFARFLLLALLGLCTCLSITAAAAADTEAASSSLTKTSSEHKAEKREKRSFDELASGL

VGKRGRWRKRPYYYRYSYKRPFGELASGLVGKRRPFGDLANGLIGKRAPFGDLANGLIGK

RRPFGDLANGLIGKRAPFDDLASGLIGKRRPFGDLANGLIGKRAPFDDLASGLIGKRAPF

GDLANGLIGKRRPFGDLANGLVGKRLHEIASGLIGKRPFDDLASGLVGKRPFDDLASGLV

GKRPFDDLASGLVGKRPFDDLASGLVGKRPFDDLASGLVGKRPFDDLASGLVGKRPFDDL

ASGLVGKRPFDDLASGLVGKRSFDDIANGLVGKRFGEIASGLIGKRPFDDIASGLVGKRS

FDNIASGLVGKRFGEIASGLIGKRPFDNIASGLVGKRSFDDIASGLVGKRPFDSIATGLV

GKRSFDDIASGLVGKRRFDDIASGLVGKRFHEIASGLIGKRSFDDIASGLVGKRPFDDLA

NGLVGKRPFDDLASGLVGKRQLDDTSSQLLDKGSEEEED*

>TRINITY_DN368481_c0_g1_i4

MAPFARFLLLALLGLCTCLSITAAAAADTEAASSSLTKTSSEHKAEKREKRSFDELASGL

VGKRGRWRKRPYYYRYSYKRPFGELASGLVGKRRPFGDLANGLIGKRAPFGDLANGLIGK

RRPFGDLANGLIGKRAPFDDLASGLIGKRRPFGDLANGLIGKRAPFDDLASGLIGKRAPF

GDLANGLIGKRRPFGDLANGLVGKRLHEIASGLIGKRPFDDLASGLVGKRPFDDLASGLV

GKRPFDDLASGLVGKRPFDDLASGLVGKRPFDDLASGLVGKRSFDDIANGLVGKRFGEIA

SGLIGKRPFDDIASGLVGKRSFDNIASGLVGKRFGEIASGLIGKRPFDNIASGLVGKRSF

DDIASGLVGKRPFDSIATGLVGKRSFDDIASGLVGKRRFDDIASGLVGKRFHEIASGLIG

KRSFDDIASGLVGKRPFDDLANGLVGKRPFDDLASGLVGKRQLDDTSSQLLDKGSEEEED

*

>TRINITY_DN368806_c0_g1_i2

IDHIGAGLVKRPIDHIGAGLVKRPIDHIGAGLVKKGIDSIGAGLIKREDYREAMDMIKSG

LEALEEDLEKRSLDSVGMGLIRRSIHNALESDNDVDLDLIKRGLDRLGSGLIKRGLDTVG

MGLIKRGIDQVGAGLIKRNSDKRLDTVGMGLIKKGLDHVGAGLVKKGLDTVGMGLIKRGL

DRLGSGLIKKSIDRIGAGLIKKGIDHIGAGLIKKDSDLAMDKKALDYIGAGLIKRDQDSD

KNEDVVNVMDDEKRGLDYVGSGLIKKDDNNNDDGKRALDYIGAGLIKRDAETAESESA*

>TRINITY_DN368820_c2_g1_i1

MDTLQQVFVVLLVAQLAFFACTPGVQAEEESSTSDLTITDQAAAHRQKRAPGWGKRSDND

NNDDELDNFEGDELLNNLEESELAEDKRAPGWGKRDFDTDKRAPGWGKRAPGWGKRAPGW

GKRAPGWGKRAPGWGKRAPGWGKRSVLAEDSEDKRAPGWGKRAPGWGKRAPGWGKRAPGW

GKRSVGVASGACQVLKEELDAYISKAVEAETQLEAFCGSNPAAFDLLSRK*

>TRINITY_DN371198_c2_g1_i1

MEMSQVIQMLTVVMTCLLVAAVWCEEQQGSQLEDMAASQPQKRSVGLPADDEAYPAVYTG

DDDMDKRSSLFRFGKRGSLFRFGKRGSLFRFGKRGSLFRFGKRGSLFRFGKRADDVADEL

EMYPDMYVSDDDLKRVVKSFHWGRETEE*

>TRINITY_DN371680_c13_g16_i1

MMRTSLVLIGLVVIALGQALPTHSNSRQKRGFRINSSSRVAHGYGKRTFNTMDDSLFPSP

SSNGLMTVQELAQLAAENPSLSEALIQKFIDADGDGIVSTQELFGVAVE*

>TRINITY_DN371683_c0_g1_i5

MEQSMMAMSAVLLAVLVSCASALPTGGSVLQSSGLSAEKRPKYMDTRDLDIFKYMLMASI

RDLVDEGQINSAVLGQGSEEDQQSEMKAVAKRMQYMGICMRRRQNTFIPYPCLRSGGR*

>TRINITY_DN371898_c0_g2_i1

MTFCCQGASPRAGGPLVTPRCVHSLSSSWRLRVTSFWFLLAAIVFVLASFTELAAAQDEL

VPPRCGGCTTVGNCRCIGEKGSRGIPGMIGPEGPQGNQGFPGPEGLPGPKGLKGEPGLPG

PLGEKGSRGKMGAPGFPGINGIPGIPGGPGPRGPPGLDGCNGTQGDPGAPGLPGLPGLEG

QPGLPGLKGSKGDAALGAPLVKGQKGEPGPPGFPGLPGAPGEDGPLGPKGESGLPGIDGI

PGLPGLKGDKGSNGVSLRGGQGPRGDKGEPGPPGLPGPFVLEPPELIQGPPGLPGKPGDK

GEPGMKGSPGQPGYPGEEGRRGLPGPKGMLGEKGVPGIAGPRGKQGFPGNPGLPGLKGDR

GMDGLPGLPGLIGMKGERGLPGLDGPSGPQGPPGRPGGKGTVGEPGPRGFPGPGGLPGDP

GLPGLPGQDGPRGLPGSEGGRGVPGPPGPQGRDGPKGERGDSGLPGLPGLDGRPGLPGRS

GPKGEPGFGGEPGRSIPGARGPPGLPGMDGIPGQKGERGPTGLPGPPGNASKGLPGLPGP

KGEKGQQGFAGLPGRDGQPGNPGGKGASGGPCDPCLPGFKGEKGSQGLDGLPGLSGERGL

PGLPGTKGEPGDDGASGLPGLPGLPGNEGLPGLPGERGLKGEPARPDVIQGPEGLKGDLG

SPGLPGEPGRSGRTGPQGPPGAPGLPGGKGEGGEKGLPGLDGRPGPSGLPGNPGESGLPG

LGLAGPPGERGDPGMNGLPGLPGLPGQKGEPGRVEGGNFKGEKGERGFDGVPGKEGIPGL

PGLPGPKGEAGLPGLPGMKGDSGEPGLSGVPGPRGLPGPRGEKGLPGLPGAPGLSGPKGD

SGLPGLPGIDGEPGLSGLPGEALQGLPGPRGNPGSPGLPGRDGEPGAPGPDGLPGLPGLK

GELGEDGLPGLPGRDGSKGDAGLPGRDGAPGEDGLPGLPGIPGEPGAKGDGGEPGFPGLS

GPKGEPGLNGRPGEPGLPGLDGRPGEPGVPGQDGLPGLPGLKGDIGVMGFPGEPGRDGES

GLPGLPGASGQKGERGFPGEPGLPGLRGPKGLDGLPGLPGMDGESGQPGLPGLPGLKGET

GLDGLPGRPGEAGRDGLPGLKGELGEPGIGEPGLPGLPGEPGRDGEPGRPGLPGIIGEKG

LPGFPGERGLKGDGGEPGSPGLPGLDGLPGMKGNSGLPGLPGTSGAKG

>TRINITY_DN375773_c0_g1_i1

MAVWGKRSTQDTHPNKKWSDMAAWGKRSTQDTHPDKKWSDMAAWGKRNAQPDKKWSDMAV

WGKRSAQDAGSEKKWSHMSTWGKRWGNVATWGKRDQSDSPSPDKKWGNMAVWGKREAPVT

KKWKNMAAWGKREHSHSDSADKKWAGLTTWGKRDSDTDTQNAEKKWSNLATWGKRDHADT

PHADTPHADDKKWSNMAVWGKRQDAEDSVQAGNRWNAVSSWGKRSRDWNTMATWGKRGSE

GFDNEASVPTPKDFLFRLIDSDGNHIVDEQELADLLAWLKSEQEHAHAQ*

>TRINITY_DN375773_c0_g2_i2

MEVRHFLYLLISAMAAMYHLTFVEASEESALDEETDSSSSSQDSQNLLENMLEKRWPQFR

SWGKKWDWKRWQALNTWGKRWNNKFATWGKRGWSQKPFVTWGKRDWSDQSFVPWGKRGWN

DQSFATWGKRGWNDKSFATWGKRGWNDKSFATWGKRGWSDRSFAPWGKRGWDDQSFATWG

KRGWGDQSFAPWGKRGWDDQSFATWGKRGWNDKSFATWGKRDWNDNSFAPWGKRGWNDQS

FATWGKRPAGDTSLQTLWIKRPSSWNQLGVWGKRGWGNHQFATWGKRDSDEDTLTTPEVE

QEEETLSAETPEKRTWNQFVTWGKRDGLHHLHKRWANVQTWGKRTSEEEGNEEDTPSETH

ATNTQATTSTDSSKTDDSEAEETASEHSEENSDSKDNASNSSDSDSETEARTDSQTKDDT

KVTNNGDKRWNTMATWGKRSPESVDKKWSDMAVWGKRSTQDTHPNKKWSDMAVWGKRSTQ

DTHPNKKWSDMA

>TRINITY_DN376049_c1_g1_i1

MGVLRVLFQRVAMTEVTPVVIAMLCMLGQVTGLRNSCGVDSRPHVKGICGDALLRARENL

CFILYTEYPEHFGRSMNKRSVTDIDQYPVHAFANMNLPARDAEKERSGTPATAENVRKLY

VSLFPKVQTLLPEMDRSLLPQAPGSQQAKRGGELAQNVQKRGMVCDCCFNQCLPSVLARY

C*

>TRINITY_DN376181_c3_g2_i1

MCCWTGTLASQILWLLITMTTLTSPFLQVDTEAGKDLLLPPLPPHSLHELATSAANSIPR

AIAPDRYSRQATLMMAASSPRIGVRSSLIGSSDTCQVDPVRMSIRPPRKYAHLCAPTTEI

SFGCRGGCPSYSRIDARNTTKVLRSCSCCRPTGFGFRLVKMKCEGFSLRTTVKFALGCHC

RPCMASVQSVDIQRLRDLLRATSIANIG*

>TRINITY_DN379841_c0_g1_i1

IWMTLGQVLSKKDLDDVGAGLIKKDLDDVGAGLIKKDLDDVGAGLIKKNQDDTDKEILRK

KLDSWGPVHKKRSEDSPEHPDVKSAFNRKEPNDEVAELANKFSDDLNSKSKEKEPDDVNM

AVTKKGSDDAGTGLVKKELDDVGSGLLKKESENKGFDLDKNGPSALKPHHFDKNPPSALK

LHHFEEKPSKEKSHLQTQAPSDDKSKMALKEK*

>TRINITY_DN379841_c2_g1_i1

MNASNTCLTLAALLLCLSHGLAFRLDWTVPNAWLSPPARYSWGLPPPSEWQHTSPSKLPV

AMGRNAGNDVSPDKAVLSSVELSKKSADSDFKEWLDSAGTEQMKSGDVHGFDIPGELQED

DPKNQAKKAETELLKQTFDKADRSLVKKYLNNKDVKQRQVQPFEMDMVKQLFSEPGMGLD

KNPFPYSDADLQKHPVIKNEKGLVEVTEQNVNTGSIHNPNEKQAALELIKKGLDAIGTGL

IKKGLDTVGSGLIKRGLTQLADSLGNGDPTKEVDIDMFEGGLDSVGAGMFTQGLDTAGLK

LIKRGMDKIEAGLVGSSSDANRQLDSGGISLMKTGFDHLETGLVKHGVDMDGLGMITEGL

EKLGKWLIKQGSDHESYDVVKATGDKKTDTTKETFADYKLIKKGFDKLVAELNTKASDDG

EPGIVKTDFNQDEMEFIKKKLHDVGAGLIKKDLDDVGAGLIKKDLDDVGAGLI

>TRINITY_DN380234_c1_g1_i1

MQTKKMWRPVCWLWLLAMALMDWTAADDSIFCDRPANMELCWWNNVNKRYLRFGRGDPFL

RFGRSQIPASWNYNPFNHFGKRSGAAAQSDELEKYLENGGYLRFGRSVSSVPLLALLSSP

EAGASIRKRRDVSEVASPVLKRTARNVQDGGFLRFGRNHADQQLTGNEDLAASLTEGLPT

KKADPAKGCVSNADREYLRFGRARSDKRNYLRFGRSTAEGSLVR*

>TRINITY_DN380234_c1_g2_i1

MELCYSTGTMPKRFLRFGRALTDDPFLRFGRNSMVDKRFLRFGRSDVDSMNVEAMLKLAL

ARAQAEAQSPVYLRKKRSAPVEPSDTKQENQAASPNHPEGVAKRDTEGGAPLLEEDKRFM

RFGRGVEGDEAVTPEEKRFMRFGKRFMRFGRRDQDDNDEVLSNDKRFMRFGKRFMRFGRN

SDDLDEADLDSDDTEQKKRFMRFGKRFMRFGRSGPQDEDLSEDKRFMRFGKRFMRFGRDP

TEAEKRFMRFGRDPTEAEKRFMRFGRDPSEAEKRFM

>TRINITY_DN381008_c7_g1_i1

KRPMNMLRLGKRDGDDEELVYPDNVFEPLMEDEDDLSAVKRPMNMLRLGKRPMNMLRLGK

RPMNMLRLGKRPMNMLRLGKRPMNMLRLGKRPMNMLRLGKRDAE*

>TRINITY_DN381008_c7_g2_i1

KRPMNMLRLGKRDGDDEELVYPDNVFEPLMEDEDDLSAVKRPMNMLRLGKRPMNMLRLGK

RPMNMLRLGKRDGDDEELVYPDNVFEPLMEDEDDLSAVKRPMNMLRLGKRPMNMLRLGKR

PMNML

>TRINITY_DN48943_c0_g1_i1

MFYAMRKLMDKLIRLMTQRYLTVVLLVVCLGLTPTPTPTTTASLINPRTTLQCHVRSYSF

RATKPPIVNENGDLVTCEGDVHVNSCWGRCDSSEIGDFKMPFKVSHHPVCTYTGRERRIV

RLSDCAGYPDPTIQVFDAAGCACRLCNSDFTSCENLNG*

>TRINITY_DN493250_c0_g1_i1

MDSDEDDSSSNIINNDGNRDDNNDIVKRVFCNGFTGCGGRHRELSRRRRIFGKRLIPVLV

KRPFCNNFGCFNSKRNPLAAPSVEGFRARLLSQGFAPAAEGETPGGKQGGVYKGKRLFCN

GYGGCRGGKRSLFSPWMSKMSSVADSLR*

>TRINITY_DN576803_c0_g1_i1

HDLQPEVMSQWNVQSPDYGALDDGLYLIDNYDDDDDFHIDGPLGLNDCIGRICGTVNANF

PNGKLSQNREASVYLNTSPGLGDKKTNPANKRVWESVGKRPLVLPYSSHGWTDDDTF

>TRINITY_DN582618_c0_g1_i1

MEHSQHLFITIVAVLLCGCVTIALPTAHSDSVQDLSHLVNLIGKLKHLDTHINDDNWALI

SNDDDDDTSQTGISTGPASPSAADKVEALRKGFLGFNKRQGFWSYDYGLGGGRFGKRYYG

DYGIGGGRFGRDVDHVDVADSNDSTL*

>TRINITY_DN68933_c0_g1_i1

RKKEKKTTTPKQTNKISPFTFVINLLSAMQHHRFVVLTFALLATTLTLAAPTEKTNKAVK

SKVRDTRDVSSGDVARLLTQLTSSELSDKEEEKEGKGTSVSKRYLDTLGGFQVHGYKRNA

GSQQESQQEDHKVDKVSVVTLDDASHPDVKRGFDDLGGFNVHGYKRGFDDLGGFNVHGYK

RGFDDLGGFNVHGYKRGFDDLGGFNVHGYKRGFDDLGGFNVHGYKRGFDDLGGFNVHGYK

RGFDDLGGFNVHGYKRGF

>TRINITY_DN119509_c0_g1_i1

MDNYAFMARLGKRGLGDEGEELALEKREAADEDAEISQNRTKRGLDGIMYSPQLGKRRMD

SMLFGPQLGKRRMDSMLFGPQLGKRRMDSMLFGPQLGKRRM

>TRINITY_DN262493_c0_g1_i1

GKLGKRAGVDTYGFWGKLGKRAGVDTYGFWGKLGKRADVDAYGSGDEVDKRGMDNYAFMA

RLGKRGLGDEGEELALEKREAADEDAEISQNRTKRGLDGIMYSPQLGKRRMDSMLFGPQL

GKRRMDSMLFGPQL

>TRINITY_DN262493_c1_g1_i1

KRRMDSMLFGPQLGKRRMDSMLFGPQLGKRRMDSMMFGPQLGKRRMDSMLFGPQLGKRRM

DSMMFGPQLGKRRMDSMLFGPQLGKRQRMDNLMFGGRLGKRNVNWAEEPAANEVGEFEGS

T*

>TRINITY_DN361429_c1_g3_i1

RSEERQEGWTASQSVTQVKPVSPADCYLLSSLLLLSLAVSTVRMVLRGCLLFYVLVSLLA

QVAVAATRKESDDRMLEEITKGGMEDPDSILEDNNALPNPLPPSLLLQLLRSEAALRQQQ

QQQQ

>TRINITY_DN327429_c0_g1_i1

AGLVKRPIDHIGAGLVKRPIDHIGAGLVKRPIDHIGAGLVKRPIDHIGAGLVKRPIDHIG

AGLVKRPIDHIGAGLVKRPIDHIGAGLVKRPIDHIGAGLVKRP

>TRINITY_DN9908_c0_g1_i1

MAGRVILYSTLMVGVVSAHASAFFLPPPHPVLFHAPLHIAPIFHVFPLPPPLKKAAPQPR

AKPLSRPFTLADAELKVPEPFPMPGRIPGGDLFLDNNLDRFIINGGNTVGGSIAVATATN

NVNSLNLVGPAQPLAVGPGLGLGGLGGLGGVGGIGGLGGLGGVGGVGGPGGLGGLGGVGG

VGGPGGLGGLGGVGGVGGLGGLGDLGGVGDVGGLGGLGDLGGVGGVGGPGGLGGLGGVGG

V

>TRINITY_DN357503_c2_g2_i1

SKSTQLGNKSKSTHLGDKSKSTTWKQIKNPFNLETNQNPFNLETNQNPINLETNKNPINL

GTNQNPINLKTNQNPINLETYQNPLNLETNQNPINLETYQNPLNLETNQNPLNLETNQN

>TRINITY_DN453329_c0_g1_i1

MACAVTFLSTSAKQMTVLVGVVVGVLLSLSVAQVNTQRVLCGRTLADTLDLVCAGRGFYF

NKRSADSAPKETVAAKEAANRVKRSMGPGGVGPVRSVVDECCRRACTYSVLESYCAPADD

TFEITQEDLADSFVGRRHTTMASTLYTTTQSTAHYDPNSPSAEVFRGPSRGVFPQNRRKF

FYVQVGSRPTRNPLISE*

>TRINITY_DN16350_c0_g1_i1

MSVTHAMVVVCVAAVWCDGSADDASKRSADSDDSAYAVYVDDDDDKRSSRGKRGSRDKKRS

>TRINITY_DN363131_c2_g1_i1

FLSLSSSCWVFDVRSPAPPSPSREDPTLWPIVRFPRTEDFHTRTTHTHPTHKMHKALLSA

LLVISLVVVEVTSQEAMLAPPDRPQEFRSPGELRRYLKALNEYYAIVGRPRFGRSVNTNK

RSVEELGELKSDE*

>TRINITY_DN106674_c6_g3_i2

TTSHGSSAAADRATMMVTTSHGSSAAADRATMMVTTSHGSSAAADRATMMVTTSHGSSAAADRTTMMVATGSRS

>TRINITY_DN55742_c0_g1_i1

PSSSLAGFGKRRFDSIAPSSSLAGFGKRRFDSIAPSSSLAGFGKRRFDSIAPSSSLAGFG

KRRFDSIAPSSSLAGFGKRRFDSIAPSSSLAGFGKRRFD

>TRINITY_DN55742_c0_g2_i1

APSSSLAGFGKRRFDSIAPSSSLAGFGKRRFDSIAPSSSLAGFGKRGMDSMAAYSSFGFG

KRRFDSIAPSSSLAGFGKRRFDSIAPSSSLAGFGKRRFDSIAPSSSLAGFGKRRFDSIAP

SSSLA
